# Mendelian randomization analysis links HLA-DR+ CD14− CD16+ monocytes to CCL19-driven ankylosing spondylitis risk

**DOI:** 10.1097/MD.0000000000048687

**Published:** 2026-05-08

**Authors:** Liyuan Bei, Jing Liao, Chunlan Tan, Xian Li, Zhenhua Yang

**Affiliations:** a Nephrology Department, The First Affiliated Hospital of Guangxi Medical University, Nanning, Guangxi, China; b Intensive Care Unit, The Second Affiliated Hospital of Guangxi University of Chinese Medicine, Nanning, Guangxi, China.

**Keywords:** ankylosing spondylitis, circulating immune cells, genome-wide association studies, inflammatory proteins, Mendelian randomization

## Abstract

Ankylosing spondylitis (AS), strongly linked to human leukocyte antigen (HLA)-B27, lacks effective treatments for many patients despite biologics. This study investigated causal links between immune cells, inflammatory proteins, and AS to identify new therapeutic targets using genetic methods. Mendelian randomization (MR) analysis was applied using large-scale genetic data. Immune cell data came from Vuckovic et al and Orrù et al, AS data from FinnGen, and inflammatory protein data from Zhao et al. Two-sample MR explored causal relationships, and mediation analysis assessed whether proteins mediated immune cell effects on AS. MR analysis identified 26 immune cell phenotypes associated with AS, with HLA DR+ CD14− CD16+ monocytes showing the strongest protective effect (odds ratio = 0.6423). Among the 5 associated inflammatory proteins, C-C motif chemokine 19 (CCL19) had the most substantial effect on AS risk (odds ratio = 2.4234). Mediation analysis revealed that HLA DR+ CD14− CD16+ monocytes influence AS risk by reducing CCL19 levels, with the proportion of the mediated effect was 17.98% (95% confidence interval = 8.23%–27.72%). Our findings suggest that HLA DR+ CD14− CD16+ monocytes are associated with a reduced risk of AS, as indicated by a negative causal effect (β = −0.4426). This protective effect is partially mediated by a decrease in CCL19 levels, a chemokine that, when elevated, increases AS risk. These results identify CCL19 and this monocyte subset as potential key players in AS pathogenesis and highlight them as promising targets for more effective, personalized therapies.

## 1. Introduction

Ankylosing spondylitis (AS) is a chronic rheumatic disease with an unclear etiology, primarily affecting the spine and sacroiliac joints. It can lead to joint pain and stiffness and, in severe cases, cause the spine to bend and fuse.^[1]^ The incidence of AS varies across regions, ranging from 0.4 to 15.0 cases per 100,000 people per year.^[2,3]^ The pathogenesis of AS is complex, involving a combination of genetic susceptibility, environmental triggers, and immune dysregulation. Among the genetic factors, the human leukocyte antigen (HLA)-B27 allele has the strongest association, being present in over 90% of AS patients.^[1]^ Despite the strong genetic link between HLA-B27 and AS, the precise mechanisms by which HLA-B27 contributes to AS remain unclear, indicating the need for further exploration of immune-related pathways in the disease. Currently, there is a significant unmet clinical need for more effective and personalized treatments for AS. While biologic therapies targeting tumor necrosis factor-α (TNF-α) and interleukin (IL)-17 have improved patient outcomes,^[4,5]^ a substantial proportion of AS patients do not achieve complete remission. This underscores the necessity of identifying novel therapeutic targets and developing more precise treatment strategies. A deeper understanding of the interactions between immune cells and inflammatory proteins could be crucial for these advancements.

Spondyloarthritis, which includes AS, is described as a polygenic autoinflammatory disease where innate immune abnormalities may play a crucial role, characterized by the abnormal activation of innate and innate-like immune cells.^[6]^ With advances in high-throughput sequencing technology, the roles of key immune cell types in AS pathogenesis are increasingly understood.^[7]^ Compared to other systemic autoimmune diseases, the innate immune system plays a more significant role in AS, with disease-susceptible areas showing abnormal activity of cells such as γδ T cells, type 3 innate lymphoid cells, and mucosal-associated invariant T cells.^[8]^ In addition, adaptive immune cells, including T cells, B cells, and natural killer (TBNK) cells, as well as myeloid cells such as macrophages and monocytes, are also involved in the inflammatory process of AS.^[9–11]^ Among these, monocytes from AS patients have shown increased transcription and expression of inflammation-related genes, suggesting abnormal activation or functional defects.^[12]^ Recent research has proposed an integrated 3-axis model governing CD8+ T cell fate in AS, shaped by HLA-B27 and the gut-joint axis, involving intermittent T cell receptor stimulation, metabolic adaptation preserving mitochondrial fitness, and co-stimulatory inputs from IL-15 and CD28.^[13]^ These findings underscore the complex interplay between genetic susceptibility and immune cell dysfunction in AS pathogenesis. Cytokines, particularly TNF-α, interferon-γ, IL-23, IL-17, and IL-22, are significantly elevated and are closely linked to AS pathogenesis.^[14–17]^

Despite these advancements, the causal relationships between circulating immune cells, inflammatory proteins, and AS remain inadequately explored. Existing observational studies are limited by small sample sizes, confounding factors, and inherent biases.^[7,18]^ While interactions between immune cells and inflammatory proteins in AS are known, the direction and strength of these interactions are not yet fully understood. Previous research has predominantly examined individual immune cells or cytokines in isolation, often overlooking the intricate network of interactions that may drive disease pathogenesis. A comprehensive analysis integrating these components is crucial for a holistic understanding of AS mechanisms.

Emerging evidence has implicated the chemokine C-C motif chemokine 19 (CCL19) in AS pathogenesis.^[19]^ CCL19, together with its receptor C-C chemokine receptor type 7 (CCR7), facilitates the migration of monocytes, mature dendritic cells (DCs), and naïve T cells to lymph nodes, playing a crucial role in adaptive immune responses.^[20–23]^ In AS patients, serum CCL19 levels are significantly elevated compared to healthy controls, and increased CCL19 expression has been detected in hip ligament tissues of AS patients undergoing total hip arthroplasty.^[19]^ Functional studies have demonstrated that exogenous CCL19 treatment does not affect ligament fibroblast proliferation but significantly up-regulates the expression of osteogenic markers, including alkaline phosphatase, osteocalcin, and the key osteogenic transcription factors Runx-2 and Osterix.^[19]^ These findings suggest that CCL19 may promote heterotopic ossification – a hallmark of advanced AS – by driving fibroblast-to-osteoblast differentiation. Furthermore, CCR7 signaling via CCL19 has been shown to activate downstream extracellular regulated kinase 5 in T cells, a pathway that modulates chemotaxis and limits the extent of humoral immune responses.^[24]^ Dysregulation of this CCL19/CCR7/extracellular regulated kinase 5 signaling axis may contribute to the persistent inflammation and aberrant tissue remodeling characteristic of AS.

Mendelian randomization (MR) provides a robust framework to address these gaps by using genetic variants as instrumental variables (IVs) to infer causality between exposures and outcomes, minimizing confounding and reverse causation.^[25]^ In this study, we employed a bidirectional MR design combined with a 2-step mediation analysis using the latest genome-wide association study (GWAS) summary statistics for circulating immune cells, inflammatory proteins, and AS. Our primary aims were: to identify specific immune cell phenotypes and inflammatory proteins with causal effects on AS, and to explore whether inflammatory proteins mediate the pathway from immune cells to AS. We hypothesize that specific immune cell subsets influence AS risk through modulation of key inflammatory proteins. Elucidating these causal relationships may deepen our understanding of AS pathogenesis and identify potential therapeutic targets for more effective, personalized treatment strategies.

## 2. Materials and methods

### 2.1. Study design

**Figure 1.**
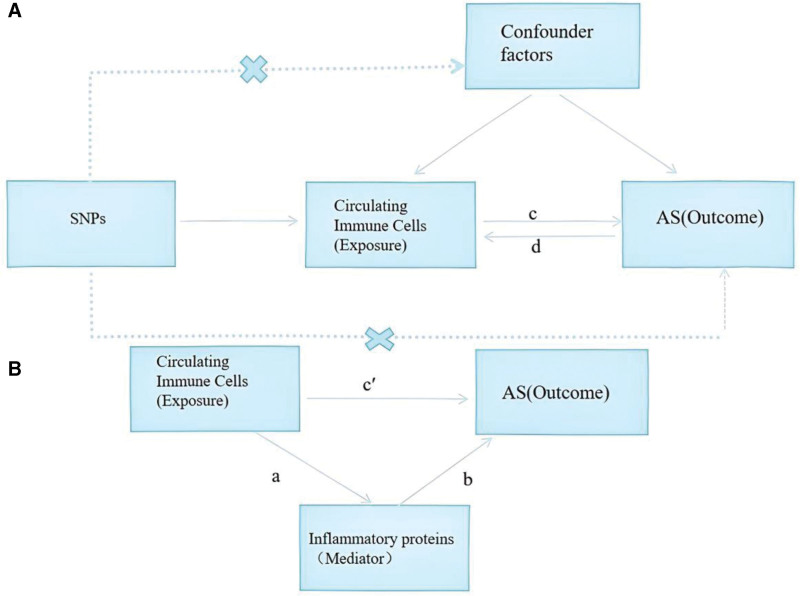
Study design of bidirectional and mediation MR analyses. This figure illustrates the analytical framework for investigating causal relationships among circulating immune cells, inflammatory proteins, and AS. Step A (bidirectional MR): assess the overall effect between circulating immune cells and AS. Path c: genetically predicted circulating immune cells serve as the exposure, and AS as the outcome, to estimate the total causal effect of immune cells on AS. Path d: genetically predicted AS serves as the exposure, and circulating immune cells as the outcome, to test for reverse causation. Step B (two-step mediation MR): indirect effect (a × b): path a represents the causal effect of circulating immune cells on inflammatory proteins (mediator). Path b represents the causal effect of the mediator on AS. The product a × b quantifies the indirect effect mediated by inflammatory proteins. Direct effect (c′): calculated as c′ = c − (a × b), representing the residual effect of immune cells on AS not mediated by the inflammatory protein. Proportion mediated: the ratio of the indirect effect to the total effect (a × b/c), expressed as a percentage, indicating the extent of mediation. All causal estimates are obtained using IVW as the primary MR method, with sensitivity analyses (MR-Egger, weighted median, etc) to assess robustness. AS = ankylosing spondylitis, IVW = inverse-variance weighting, MR = Mendelian randomization, SNP = single-nucleotide polymorphism.

In this study, we adhered to the 3 core principles of MR analysis^[18]^ to ensure the rigor of causal inference. The genetic IVs we selected, specifically single-nucleotide polymorphisms (SNPs), have a significant genetic association with circulating immune cells (exposure factors). The chosen IVs are not associated with other potential confounding factors that might influence both circulating immune cells and AS. The IVs influence AS indirectly through their effect on circulating immune cells rather than having a direct effect on AS. Based on these principles, we designed and executed the following MR analysis workflow (Fig. 1): bidirectional MR analysis: initially, we employed a 2-sample MR approach to explore the potential causal relationships between 731 phenotypes of circulating immune cells, 91 inflammatory proteins, and AS. Mediation MR analysis: furthermore, we investigated the role of inflammatory proteins as potential mediators in the relationship between circulating immune cells and AS by constructing mediation models.

### 2.2. Data sources

Our data on circulating immune cells were derived from a European population-based study comprising 6602 individuals native to the central east coast of Sardinia, Italy, with a sex distribution of 57% female and 43% male and ages ranging from 18 to 102 years.^[26]^ From this cohort, 3757 participants underwent detailed immune profiling, with a total of 731 immunophenotypes analyzed.^[26]^ These immunophenotypes include 118 absolute cell counts, 389 median fluorescence intensities reflecting surface antigen levels, 32 morphological parameters, and 192 relative cell counts.^[26]^ The features encompass B cells, DCs, T cell maturation stages, monocytes, myeloid cells, TBNK cells, and regulatory T cell panels.^[26]^ The study assessed approximately 22 million genetic variants and identified 122 significant independent association signals at 70 genetic loci.^[26]^ Detailed information on all 731 immune cell phenotypes is provided in Table S1, Supplemental Digital Content.

The data related to AS in this study were sourced from GWAS summary data available at GWAS catalog (https://gwas.mrcieu.ac.uk/), specifically the dataset ID “finngen-b-M13_ANKYLOSPON_STRICT.” The dataset includes 599 cases of AS and 217,431 control individuals, all of European descent, encompassing 16,380,466 SNPs. More detailed information about the dataset can be found at https://gwas.mrcieu.ac.uk/datasets/finn-b-M13_ANKYLOSPON_STRICT/.

Inflammatory proteins data were derived from the study by Zhao et al,^[27]^ which conducted a genome-wide protein quantitative trait locus analysis of 91 plasma inflammatory proteins in 14,824 European-ancestry participants (Table S2, Supplemental Digital Content). This study used the Olink Target platform to measure these proteins and identified 180 protein quantitative trait locus loci, including 59 cis-acting elements and 121 trans-acting elements.

This research is based on publicly available GWAS data. All involved studies have been approved by the respective institutional review boards, and informed consent was obtained from participants or their caregivers, legal guardians, or other authorized representatives.

### 2.3. Selection of IVs

In this study, to satisfy the relevance assumption of MR analysis (as illustrated in Fig. 1), we used R software to screen SNP data that are strongly associated with circulating immune cells and inflammatory proteins. The initial screening criterion was a *P* value <1 × 10E-05 to identify candidate IVs. This threshold, while more lenient than the genome-wide significance level (*P* < 5 × 10^−8^), is commonly used in MR studies of complex traits (e.g., immune cell phenotypes) where the number of genome-wide significant SNPs is often insufficient for analysis. The strength of the selected IVs was subsequently verified using the *F*-statistic. To avoid multicollinearity, SNPs within a 10,000 kb range that had an *R*^2^ value >0.001 with the most significant SNP were excluded. This clumping window of 10,000 kb with *r*^2^ < 0.001 is a standard parameter in the ieugwasr::ld_clump function (default in the TwoSampleMR package), ensuring that SNPs are in very low linkage disequilibrium across a broad genomic region, which is particularly important for trans-acting variants. *R*^2^ is an indicator of the proportion of variance in the exposure variable explained by the SNP, calculated as: *R*^2^ = 2 × (1 − MAF) × MAF × (β_1_/SD)^2^, where MAF is the minor allele frequency, β_1_ is the regression coefficient of the SNP on the exposure variable, and SD is the standard deviation of the exposure variable. We selected SNPs with an *F*-statistic >10 as valid IVs to ensure a sufficiently strong association with the exposure variable. The *F*-statistic is calculated as: *F* = (N − *k* − 1) × *R*^2^/(1 − *R*^2^), where N is the sample size and *k* is the number of SNPs in the model. To ensure each IV was associated with the same effect allele, we harmonized the summary statistics and excluded palindromic SNPs (SNPs with the same sequence in both forward and reverse directions). In the meta-analysis, if the β value of the combined effect of the SNP (indicating the estimated association between the genetic instrument and the outcome) was >0, the genetic variant was considered positively associated with the outcome; if the β value was <0, it was considered negatively associated with the outcome.

### 2.4. Causal associations between circulating immune cells, inflammatory proteins, and AS

To thoroughly investigate the potential causal relationships between circulating immune cells, inflammatory proteins, and AS, we conducted a series of bidirectional MR analyses. Utilizing R software (version 4.3.1) and its extensive packages (http://www.Rproject.org), we performed comprehensive statistical analyses. Specifically, we employed the 2-sample MR method and used the MR-PRESSO package to enhance the accuracy of our analyses.

During the traditional MR analysis, we primarily used the inverse-variance weighting (IVW) method to estimate effect sizes. To further verify the robustness of the IVW results, we also conducted supplementary analyses using MR-Egger regression, the weighted median method, the weighted mode method, and the simple model method.

The results of the analyses are presented in the form of β values and standard errors for continuous outcomes, while for binary outcomes, we report odds ratios (ORs) and 95% confidence intervals (CIs). Statistically, a *P* value <.05 was considered significant. In the IVW analysis, if no heterogeneity was detected – assessed using Cochran *Q* statistic test indicating no significant heterogeneity – we employed a fixed-effect model for the analysis. We did not apply formal multiple testing correction (e.g., FDR or Bonferroni) for the analysis of 731 immune cell phenotypes and 91 inflammatory proteins, as this study was primarily exploratory. However, we note that the key findings – HLA DR+ CD14− CD16+ monocytes (*P* = 8.44 × 10^−7^) and CCL19 (*P* = 1.75 × 10^−8^) – remain statistically significant even after applying the most stringent Bonferroni correction (thresholds: 0.05/731 = 6.84 × 10^−5^ for immune cells; 0.05/91 = 5.49 × 10^−4^ for proteins). The other 25 immune cell associations with *P* values between .05 and 6.84 × 10^−5^ are presented as exploratory findings that warrant further investigation.

### 2.5. Mediation analysis of the “circulating immune cells-inflammatory proteins-AS” association

To further investigate the role of inflammatory proteins in mediating the relationship between circulating immune cells and AS, we conducted a 2-step mediation analysis (as shown in Fig. 1). This approach decomposes the total effect into an indirect effect (mediated by the mediator) and a direct effect (not mediated by the mediator). Specifically, the overall impact of immune cells on AS is divided into: the direct effect of immune cells on AS (pathway c′ in Fig. 1); and the indirect effect mediated by inflammatory proteins (pathway a × b in Fig. 1). We calculated the percentage of the mediation effect, which is the ratio of the indirect effect to the total effect. This 2-step MR mediation analysis assumes that no unmeasured confounders affect the relationship between the mediator (inflammatory protein) and the outcome (AS). Since this assumption cannot be tested directly, our mediation results should be interpreted as suggestive evidence of a causal pathway rather than definitive proof. We have discussed this limitation in the Discussion section.

### 2.6. Sensitivity analysis

In this MR study, to validate the robustness of our results, we used the MR-PRESSO package to detect outliers in the data. After removing the outliers, we reanalyzed the data. We applied the leave-one-out method, sequentially excluding each SNP to assess the influence of each SNP on the overall causal estimate. In addition, we used the intercept of the MR-Egger regression model to statistically test for horizontal pleiotropy.^[28]^ Finally, we employed Cochran *Q* test to evaluate the presence of statistical heterogeneity among the estimates from different IVs. If the *P* value was <.05, it indicated the presence of heterogeneity, suggesting that the effects of different IVs on the outcome variable were inconsistent. We set the significance level α at 0.05. Results with *P* values <.05 were considered statistically significant.

## 3. Results

### 3.1. Causal relationship between circulating immune cells and AS

A total of 545 SNPs were used as IVs (Table S3, Supplemental Digital Content). The effect of each SNP on AS is detailed in Table S4 and Figure S1, Supplemental Digital Content. Importantly, all selected SNPs had *F*-statistics >10, indicating their suitability as robust instruments for MR analysis.

The IVW analysis revealed significant associations between 26 different immune cell phenotypes and AS. These phenotypes were categorized into 7 main groups: 2 monocyte-related, 4 TBNK cells-related, 6 B-cell-related, 6 regulatory T-cell-related, 4 T-cell maturation stage-related, 2 DC-related, and 2 myeloid cell-related phenotypes. Although no formal multiple testing correction was applied for the 731 immune cell phenotypes, the key association described below remains significant after Bonferroni correction (threshold: 0.05/731 = 6.84 × 10^−5^). The results from MR-Egger, weighted median, weighted mode, and simple mode methods are presented in Table S5, Supplemental Digital Content.

**Figure 2.**
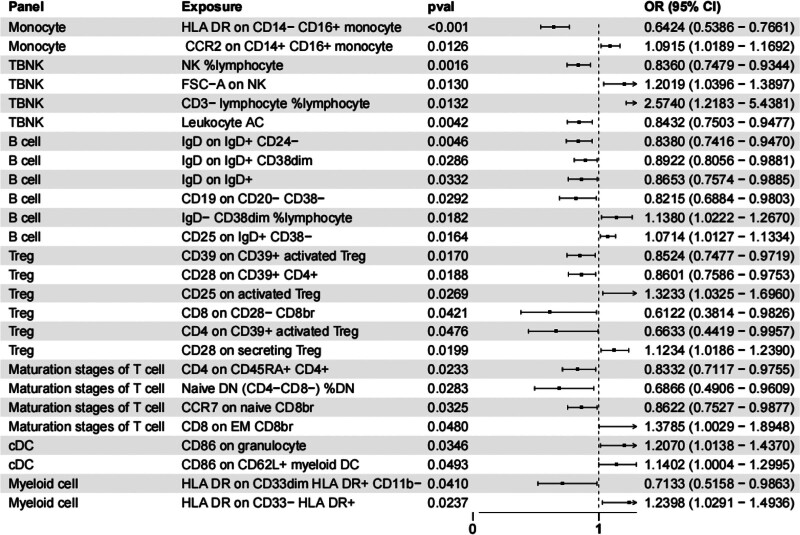
Forest plot of the causal effects of circulating immune cell phenotypes on AS risk using IVW method. Each row represents 1 immune cell phenotype that showed a significant association with AS (*P* < .05). The square indicates the point estimate of the OR, and the horizontal line represents the 95% CI. An OR < 1 (left of the vertical dashed line) indicates a protective effect, while an OR > 1 indicates a risk effect. The phenotype “HLA DR on CD14− CD16+ monocyte” shows the strongest protective effect (OR = 0.6423, 95% CI = 0.5386–0.7661; *P* = 8.44 × 10^−7^). AS = ankylosing spondylitis, CI = confidence interval, DC = dendritic cell, HLA = human leukocyte antigen, IVW = inverse-variance weighting, NK = natural killer, OR = odds ratio.

As shown in Figure 2, HLA DR on CD14− CD16+ monocyte had a particularly significant impact on AS risk, with an OR of 0.6423, a 95% CI of 0.5386 to 0.7661, and a *P* value of 8.44E-07. An OR <1 indicates a protective effect, meaning that genetically predicted higher levels of these monocytes are associated with a 35.77% reduction in the odds of developing AS. Among the 26 significant immune cell phenotypes, the majority exhibited protective effects (OR < 1), while only a few showed risk effects (OR > 1). The forest plot clearly demonstrates that “HLA DR on CD14− CD16+ monocyte” has the narrowest CI and the lowest OR, indicating the most robust protective association. The MR-Egger, weighted median, and weighted mode methods supported the results from the IVW method, and the consistency of effect directions across these methods further supports the reliability of this finding. This finding prompted further exploration of the potential role of this immune cell subset in the immune cell-inflammatory protein-AS pathway.

To visualize the overall distribution of causal effects, we generated a volcano plot (Fig. S4, Supplemental Digital Content), which highlights the strong protective effect of HLA DR on CD14− CD16+ monocytes against a background of 730 other immune cell phenotypes. The volcano plot reveals that most phenotypes cluster around the null effect (β = 0) with *P* > .05. However, “HLA DR on CD14− CD16+ monocyte” stands out as an extreme outlier in the protective direction (negative β) with a very low *P* value, confirming its unique and robust association with AS.

To verify reverse causation, we performed reverse MR analysis to investigate the impact of AS on HLA DR on CD14− CD16+ monocyte. The results indicated no significant association (*P* > .05).

### 3.2. Causal relationship between inflammatory proteins and AS

Through MR analysis, a total of 133 SNPs were used as IVs (Table S6, Supplemental Digital Content). The effect of each SNP on AS is detailed in Table S7 and Figure S2, Supplemental Digital Content. All selected SNPs had *F*-statistics >10.

**Figure 3.**
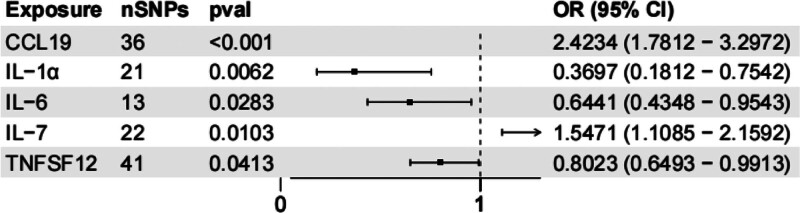
Forest plot of the causal effects of inflammatory proteins on AS risk using the IVW method. Each row represents 1 inflammatory protein that showed a significant association with AS (*P* < .05). The square indicates the OR point estimate, and the horizontal line represents the 95% CI. An OR > 1 indicates a risk effect. CCL19 shows the strongest risk effect (OR = 2.4234, 95% CI = 1.7812–3.2972; *P* = 1.75 × 10^−8^). AS = ankylosing spondylitis, CCL19 = C-C motif chemokine 19, CI = confidence interval, IL= interleukin, IVW = inverse-variance weighting, OR = odds ratio, SNP = single-nucleotide polymorphism, TNFSF12 = tumor necrosis factor ligand superfamily member 12.

We identified 5 inflammatory proteins associated with AS: CCL19, IL-1α, IL-6, IL-7, and tumor necrosis factor ligand superfamily member 12. Among these, CCL19 had the most significant effect on AS (OR = 2.4234, 95% CI = 1.7812–3.2972; *P* = 1.75E-08). An OR >1 indicates a risk effect; thus, elevated CCL19 levels are associated with a 142.34% increase in AS odds. The MR-Egger, weighted median, and weighted mode methods supported the IVW findings. Detailed results are presented in Figure 3 and Table S8, Supplemental Digital Content.

### 3.3. Mediation analysis of potential inflammatory proteins

We used a 2-step MR mediation analysis to explore whether CCL19 mediates the relationship between HLA DR on CD14− CD16+ monocyte and AS (Fig. 1). In the first step (pathway a), 17 SNPs were used as IVs (Table S9, Supplemental Digital Content), and the effect of each SNP on CCL19 levels is detailed in Table S10, Supplemental Digital Content. The IVW analysis showed an OR of 0.9108 (95% CI = 0.8312–0.9980; *P* = .0453), suggesting that this monocyte subset reduces CCL19 levels (βa = −0.0933). In the second step (pathway b), using genetic instruments associated with CCL19 levels, IVW analysis indicated a significant association between increased CCL19 levels and increased AS risk (OR = 2.3451, 95% CI = 1.5159–3.6279; *P* = 1.29 × 10^−4^; βb = 0.8523). Combining the 2 steps, the total effect (βc) was −0.4426, the mediated effect (βab) was −0.0795, and the direct effect (βc′) was −0.3630. The proportion of the mediated effect was 17.98% (95% CI = 8.23%–27.72%). This suggests that CCL19 plays a significant mediating role in the association between HLA DR on CD14− CD16+ monocyte and AS.

### 3.4. Sensitivity analysis

Cochran *Q* test for all immune cells and inflammatory proteins yielded *P* values >.05, indicating no heterogeneity. Funnel plots (Figs. S1–S3, Supplemental Digital Content) showed no asymmetry. MR‑Egger intercepts were near zero with *P* > .05, suggesting no horizontal pleiotropy. The MR‑PRESSO test also indicated no outliers (*P* > .05). Leave‑one‑out analyses demonstrated that no SNP unduly influenced the results (Figs. S1–S3, Supplemental Digital Content), confirming the robustness of our MR findings.

## 4. Discussion

This study conducted a comprehensive MR analysis to explore causal relationships between circulating immune cells, inflammatory proteins, and AS. Our key findings include a protective effect of HLA DR on CD14− CD16+ monocytes (OR = 0.6423) and a risk effect of CCL19 (OR = 2.4234). Two-step mediation analysis suggested that these monocytes may reduce AS risk partly by lowering CCL19 levels, with a mediation proportion of 17.98% (95% CI = 8.23%–27.72%).

In this study, HLA DR on CD14− CD16+ monocytes showed a significant negative causal effect on AS (β = −0.4426), indicating a protective direction: genetically predicted higher levels of these cells are associated with reduced AS risk. This finding aligns with a previous study showing that HLA-DR expression on CD14− CD16+ monocytes was negatively correlated with uveitis risk in AS (OR = 0.921).^[29]^ In addition, the HLA-DRB1*13 allele has been negatively associated with AS susceptibility.^[30,31]^ However, the literature on HLA-DR in AS is inconsistent: some studies report little impact on disease severity,^[32]^ while others suggest associations with more severe peripheral joint involvement^[25]^ or later age of onset (HLA-DR1 in Mexican patients).^[33]^ Monocytes are typically divided into classical (CD14hiCD16−), intermediate (CD14hiCD16+), and nonclassical (CD14dimCD16+) subsets.^[34]^ The specific subset we identified – HLA DR on CD14− CD16+ monocytes – is relatively rare and may represent a regulatory population. Our genetic prediction analysis suggests a protective role, which contrasts with some mouse models where nonclassical monocytes promoted joint destruction.^[35]^ Thus, the function of this subset in AS warrants further investigation.

This study also identified 5 inflammatory proteins associated with AS, with CCL19 showing the strongest risk effect. Of note, CCL19 is often described as a pro-inflammatory chemokine, but its role is context-dependent: it can also participate in immune homeostasis and tissue repair depending on the biological setting.^[36,37]^ In AS, elevated serum CCL19 levels have been reported,^[19]^ and increased CCL19 and CCL21 promote fibroblast ossification in hip ligament tissues.^[19]^ Consistent with our mediation finding, monocytes can differentiate into DCs and macrophages that secrete CCL19.^[38]^ Through its receptor CCR7, CCL19 facilitates migration of immune cells to lymph nodes.^[19,39]^ Given that existing biologic therapies (e.g., TNF-α and IL-17 inhibitors) do not induce complete remission in all patients, targeting the CCL19-CCR7 axis could represent an alternative or adjunctive strategy, though this remains speculative.

This study has several strengths. First, MR reduces confounding and reverse causation, and we applied multiple sensitivity analyses (MR-PRESSO, leave-one-out, MR-Egger, Cochran *Q*) to ensure robustness. Second, using large-scale GWAS summary data enhances statistical power and reproducibility. Third, we systematically assessed 731 immune cell phenotypes and 91 inflammatory proteins, providing a broad perspective.

The study also has several limitations. First, the study primarily includes participants of European ancestry, limiting generalizability to other populations. Second, the immune cell data are derived from a Sardinian cohort.^[26]^ Although Sardinians are of European descent, they may harbor population-specific genetic architectures, which could affect the generalizability of our findings to other European groups. Third, although MR mitigates measured confounding, unmeasured confounders (e.g., lifestyle, environmental factors) cannot be fully excluded. Fourth, the *P* value for the effect of HLA DR on CD14− CD16+ monocytes on CCL19 levels (*P* = .0453) is close to the significance threshold, warranting confirmation with larger samples. Fifth, the mediation proportion of CCL19 is only 17.98%, indicating that other unknown mediators or pathways exist. Sixth, we did not apply formal multiple testing correction for all 731 immune cell phenotypes, which may increase type I error risk for secondary findings. However, our main conclusions (HLA DR+ CD14− CD16+ monocytes and CCL19) survive Bonferroni correction. Seventh, our 2-step mediation MR analysis assumes no unmeasured confounding between the mediator (CCL19) and AS. This assumption is strong and may not hold; therefore, the mediation finding should be considered exploratory and requires experimental validation. Finally, while our findings suggest causal relationships, they are based on genetic instruments and require mechanistic and experimental studies to confirm.

## Acknowledgments

We sincerely appreciate the data contribution from the IEU Open GWAS project.

## Author contributions

**Writing – original draft:** Liyuan Bei.

**Project administration:** Jing Liao.

**Writing – review & editing:** Jing Liao.

**Data curation:** Chunlan Tan.

**Formal analysis:** Xian Li.

**Conceptualization:** Zhenhua Yang.


























