# Supplementary material for: Mendelian randomization analysis links HLA-DR+ CD14− CD16+ monocytes to CCL19-driven ankylosing spondylitis risk
Source: Medicine (Baltimore). 2026 May 8;105(19):e48687. doi: 10.1097/MD.0000000000048687 (PMC13166580; doi:10.1097/MD.0000000000048687)
Supplement: Supplementary file 5 [file medi-105-e48687-s005.doc]

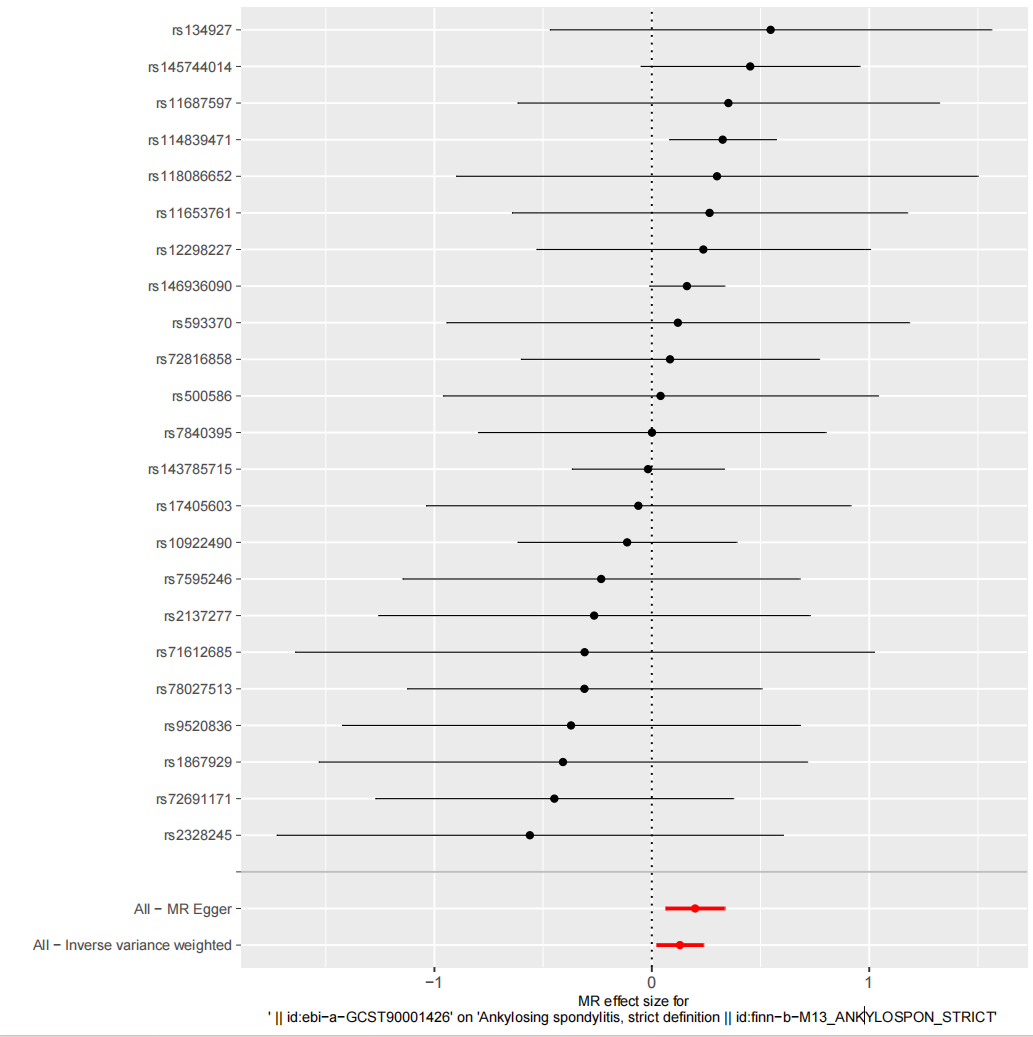

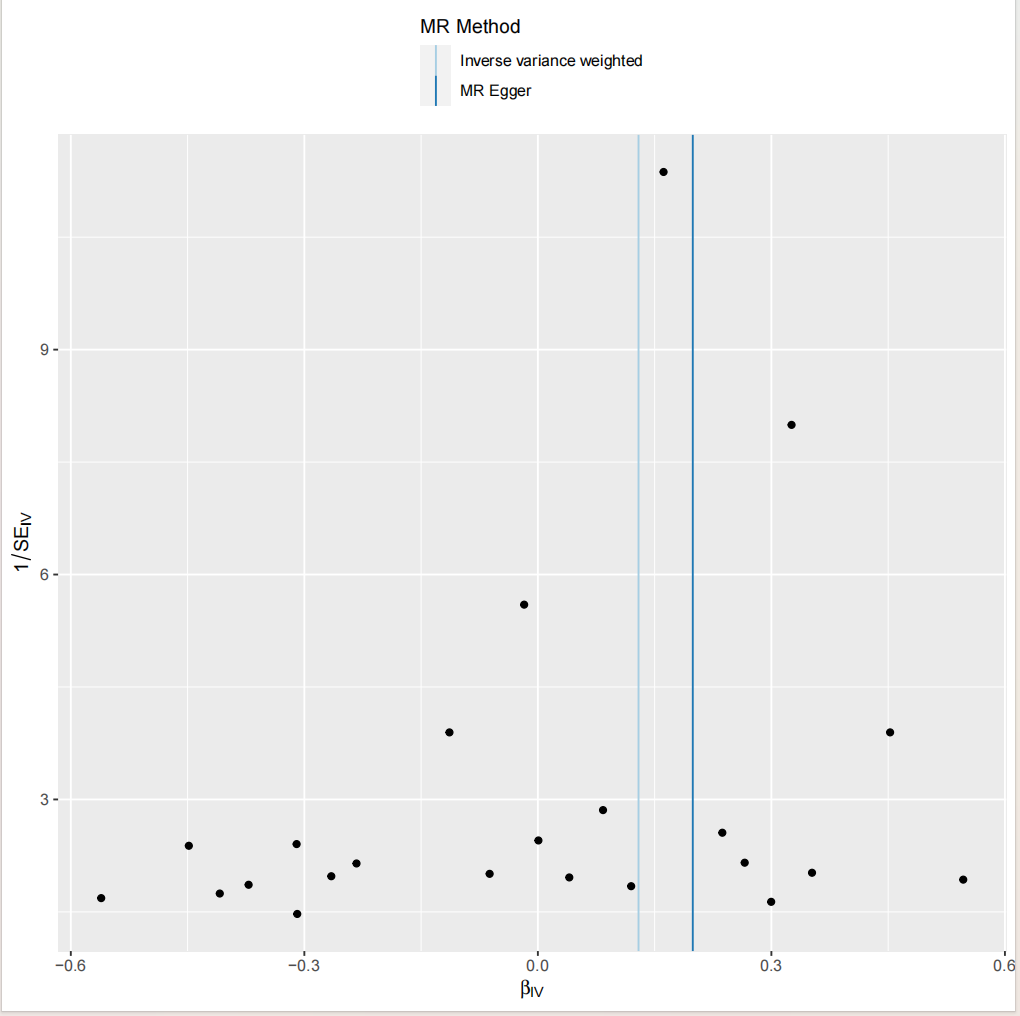

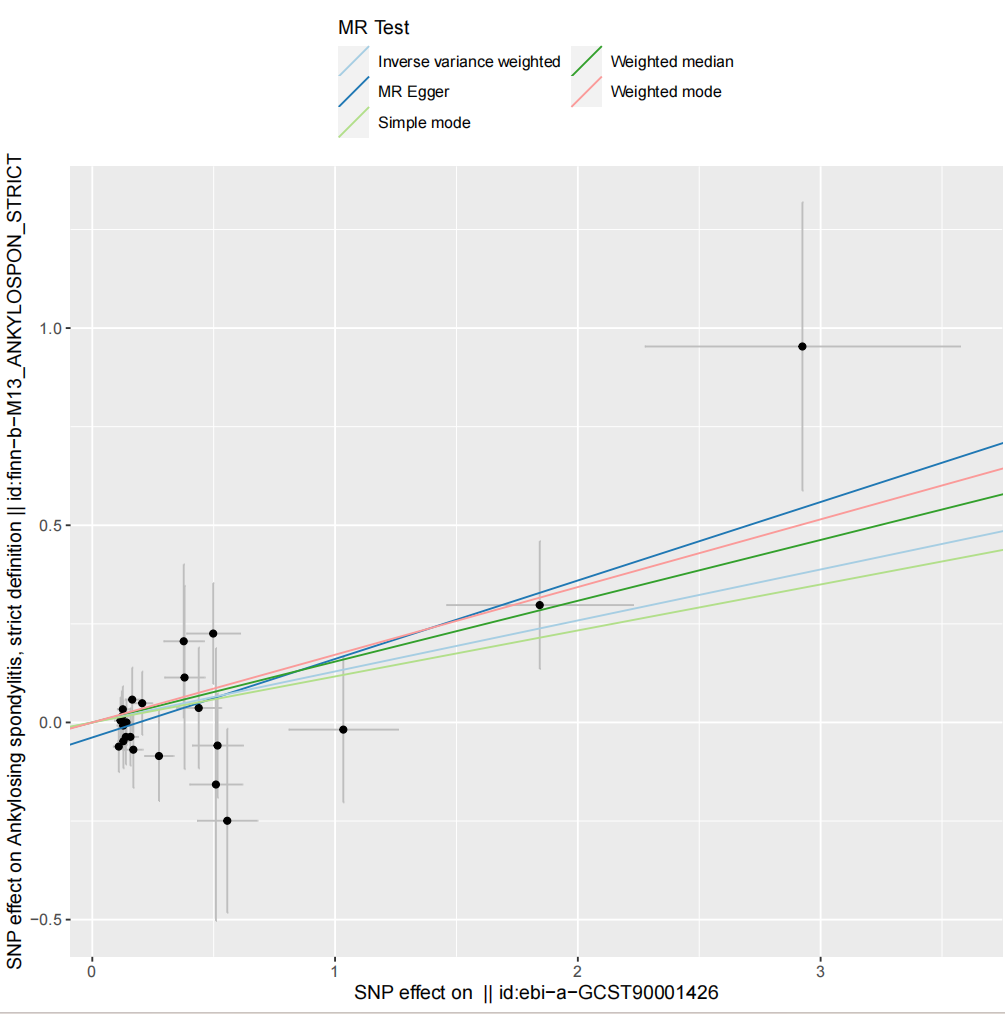

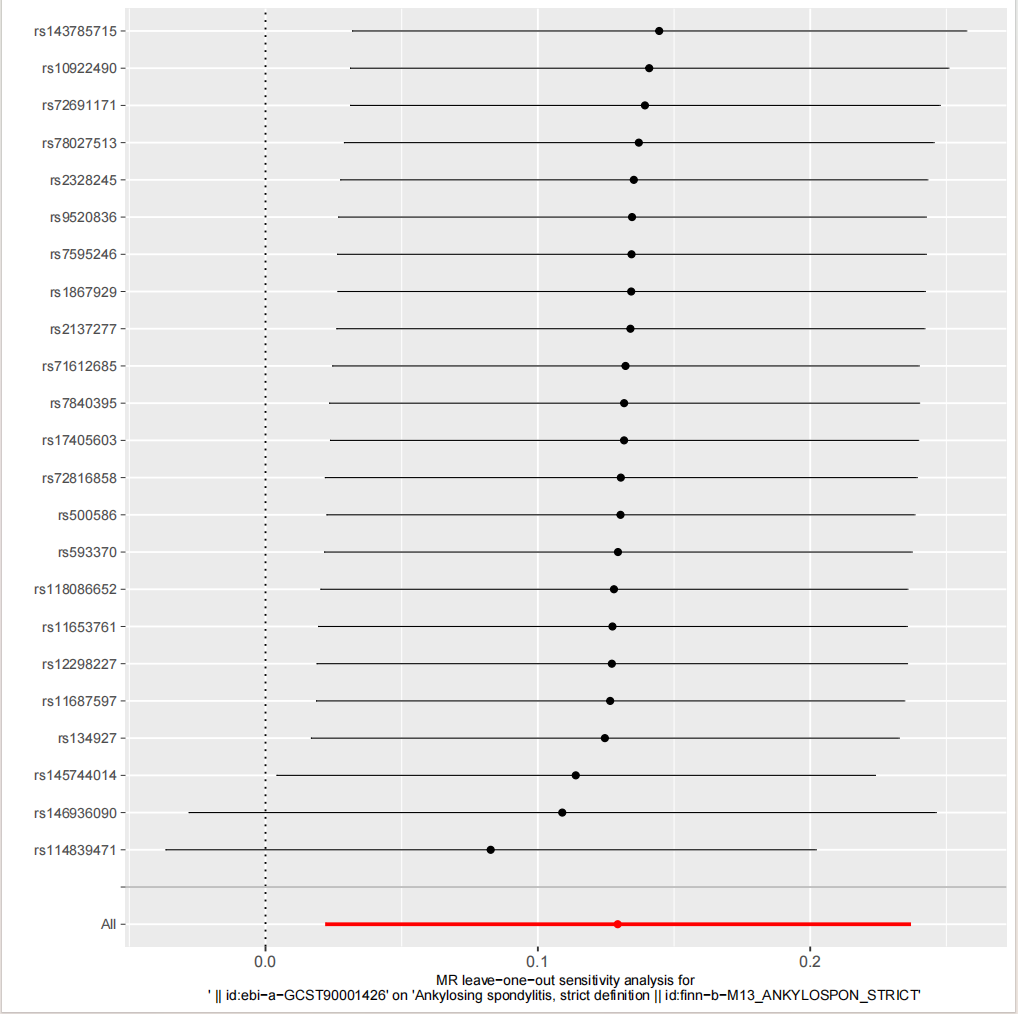


Supplementary Figure S1 Forest plot, funnel plot, scatter plot and sensitivity analysis of SNPs associated with IgD-CD38dim %lymphocyte on AS.


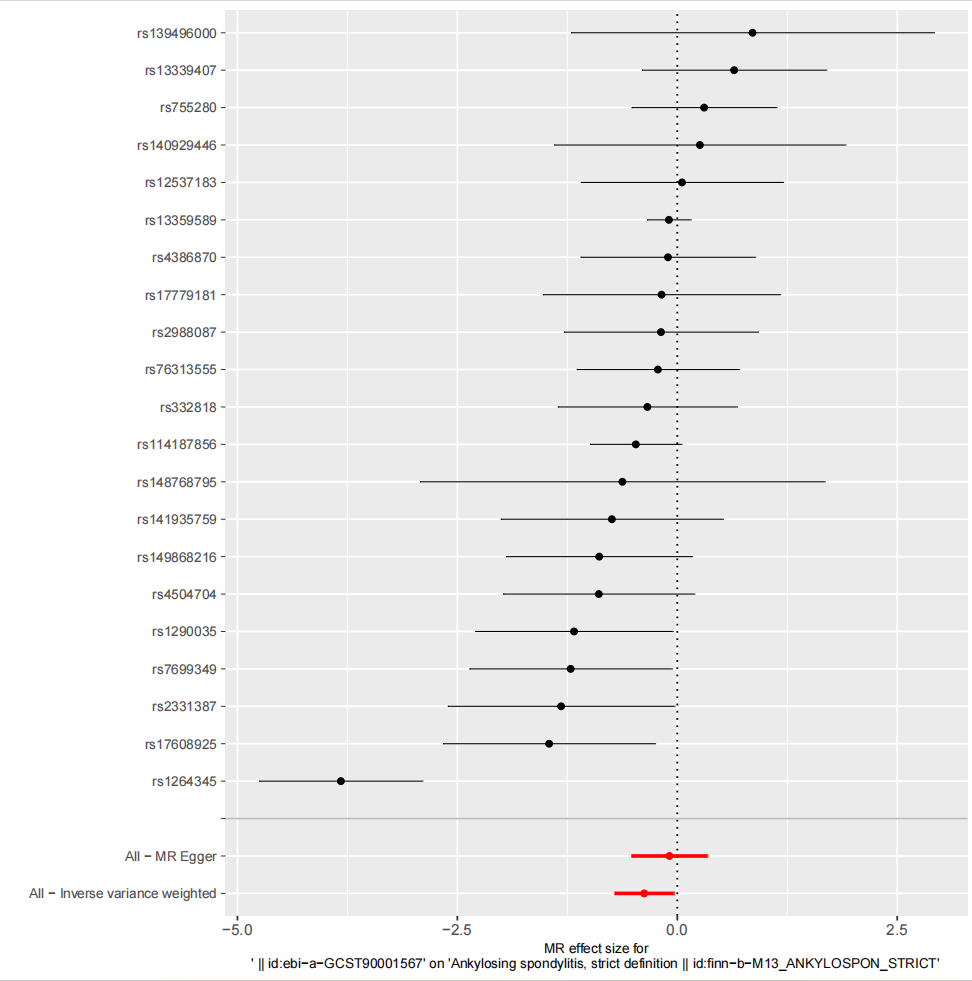

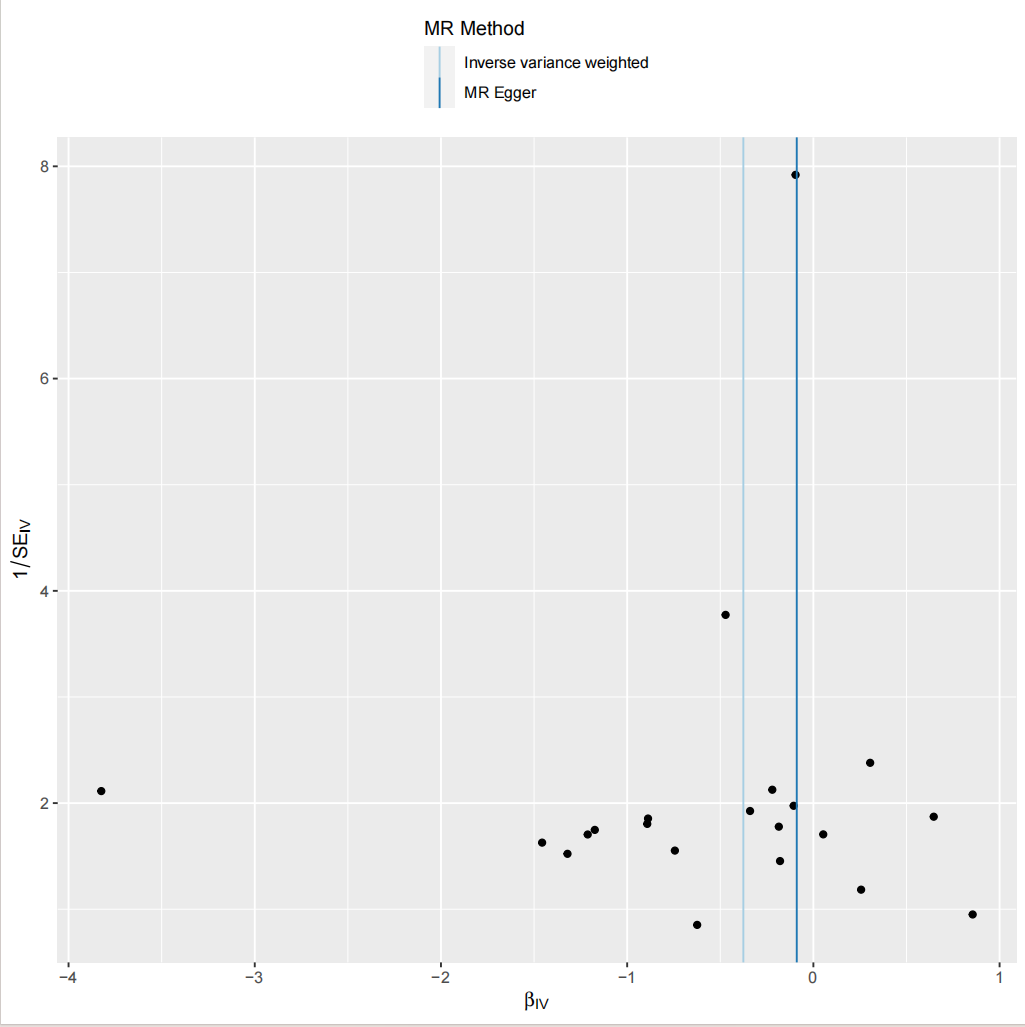

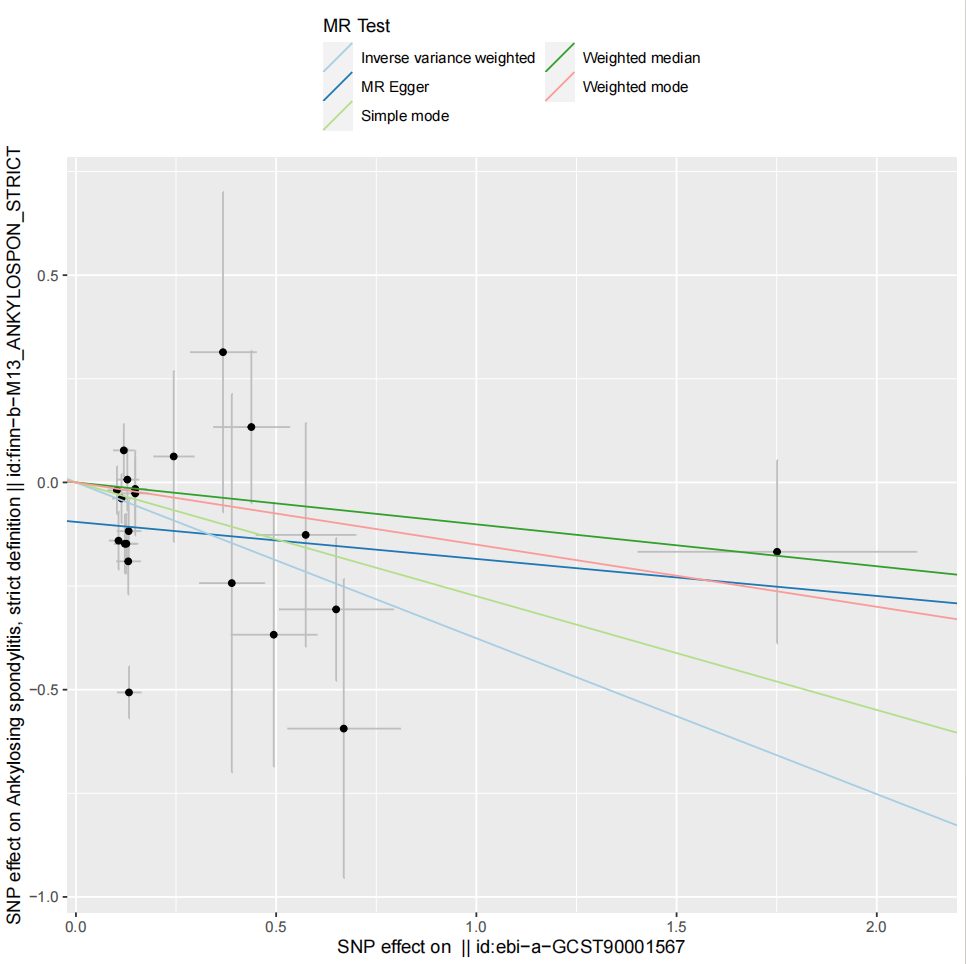

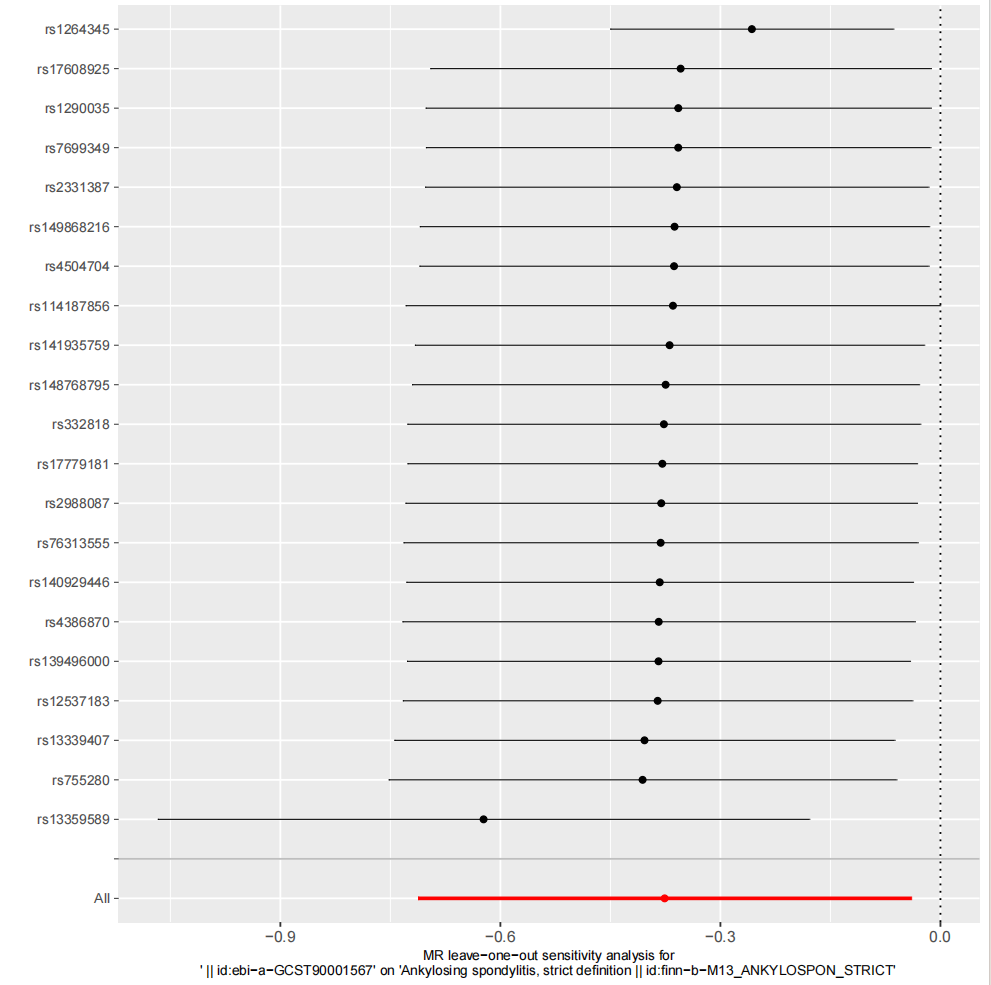


Supplementary Figure S1B Forest plot, funnel plot, scatter plot and sensitivity analysis of SNPs associated with Naive DN (CD4-CD8-) %DN on AS.


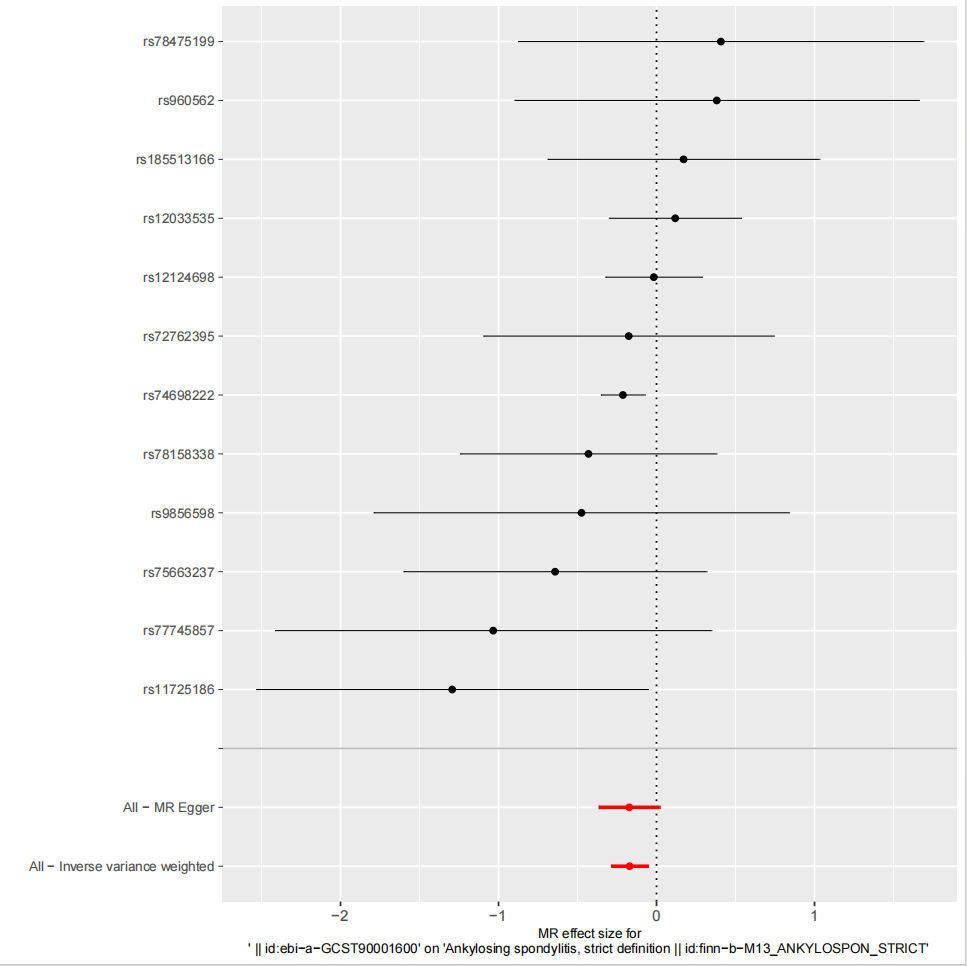

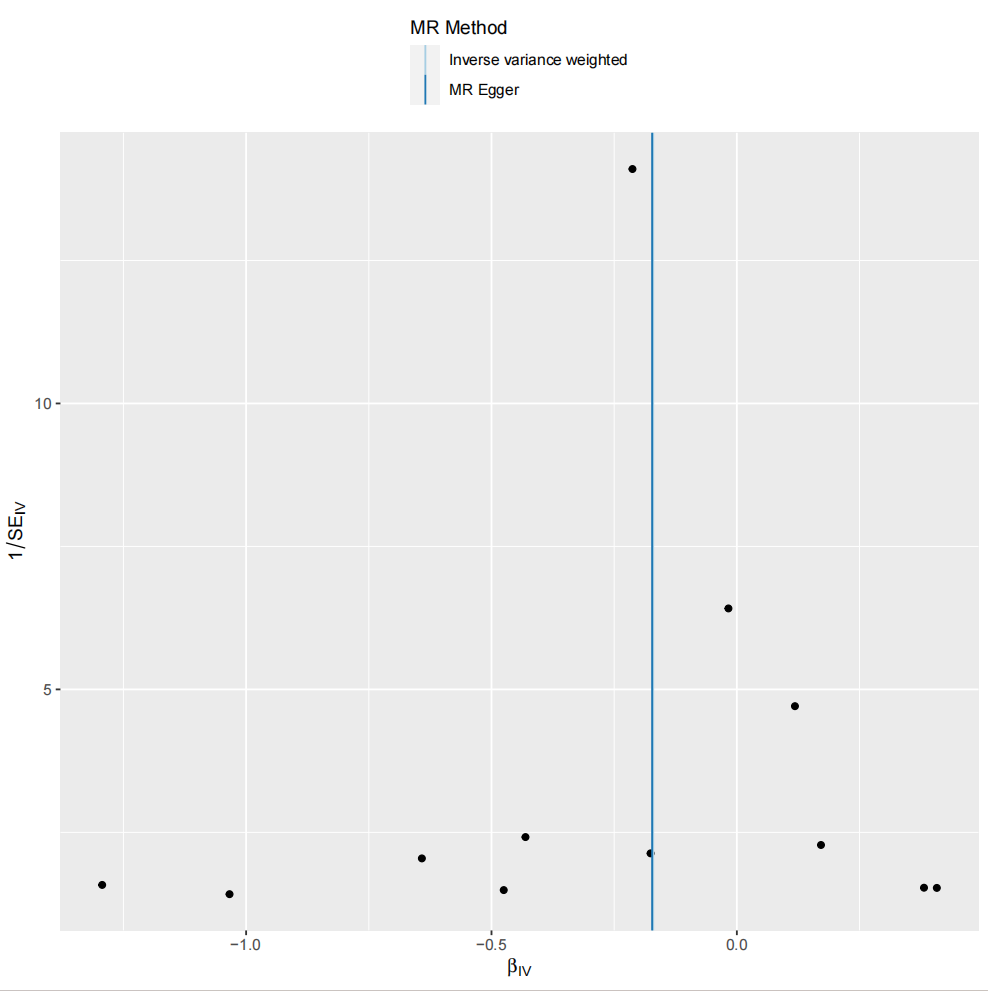


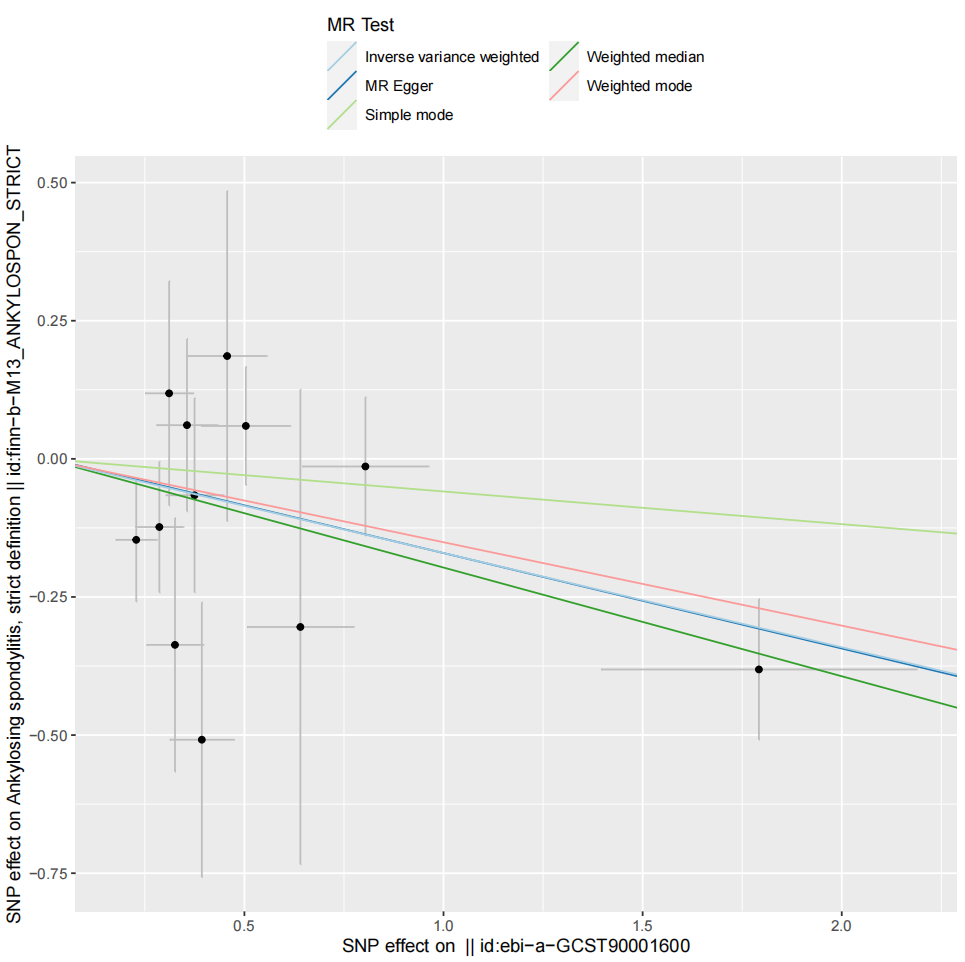

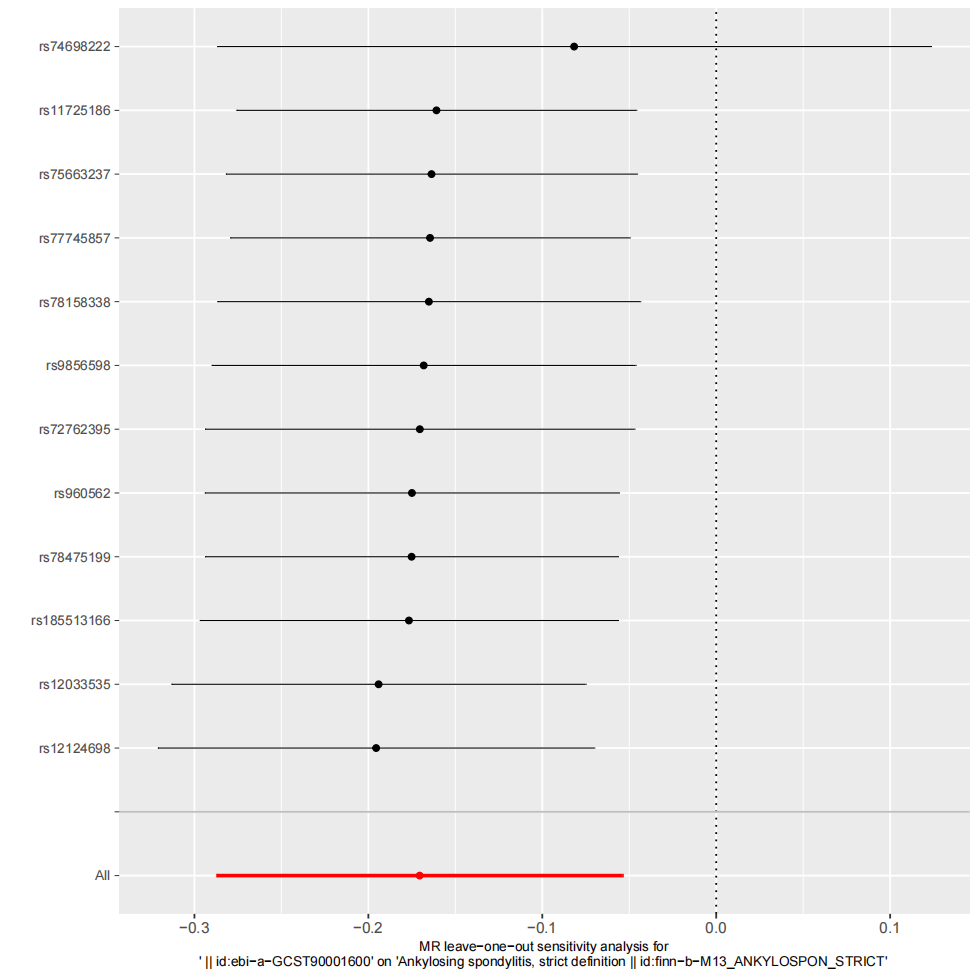


Supplementary Figure S1C Forest plot, funnel plot, scatter plot and sensitivity analysis of SNPs associated with Leukocyte AC on AS.


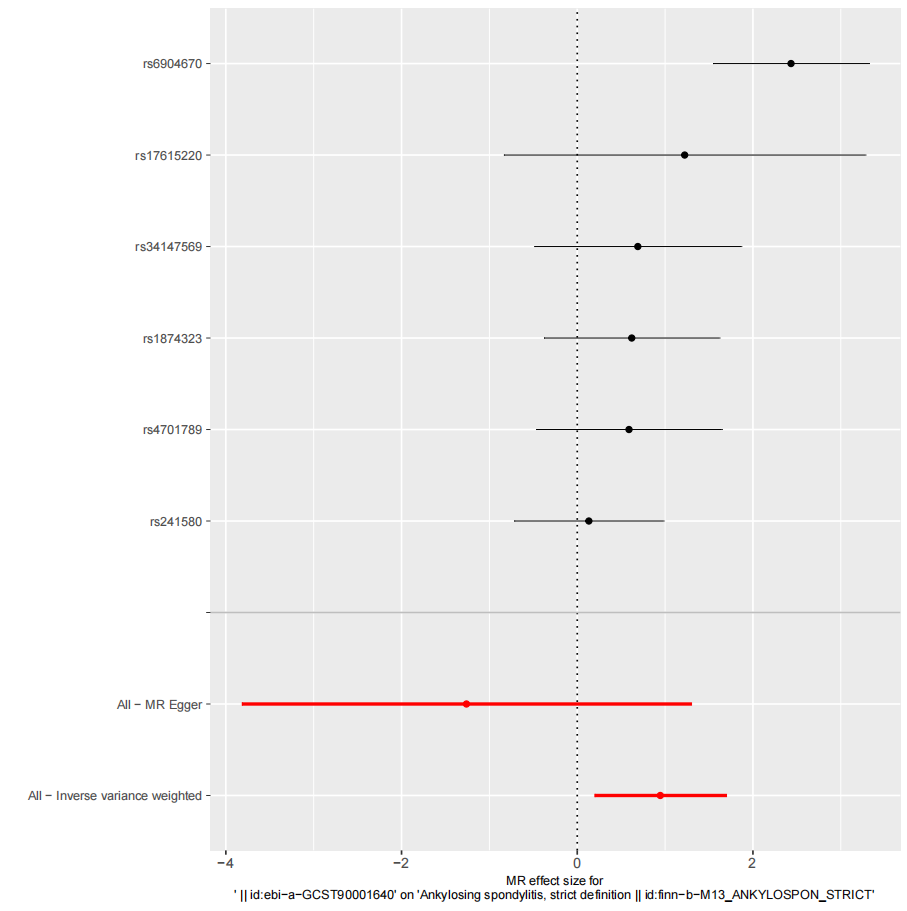

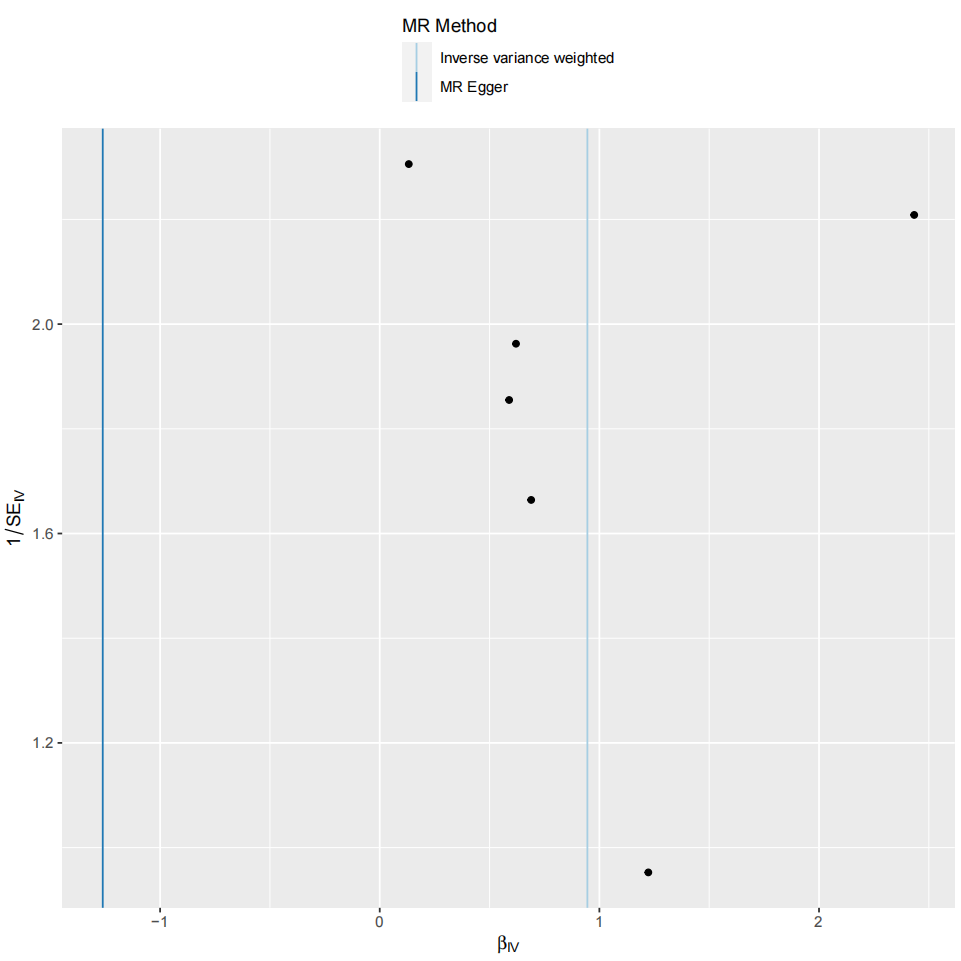

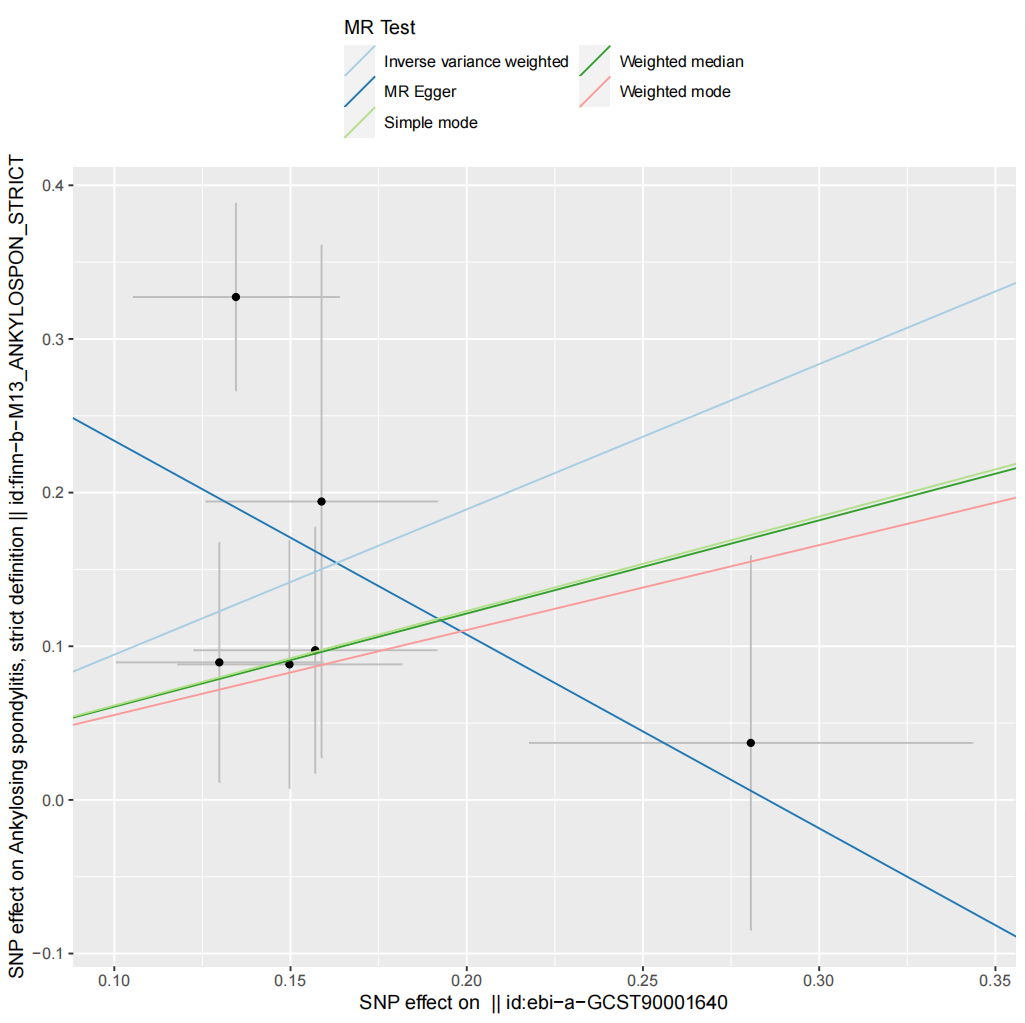

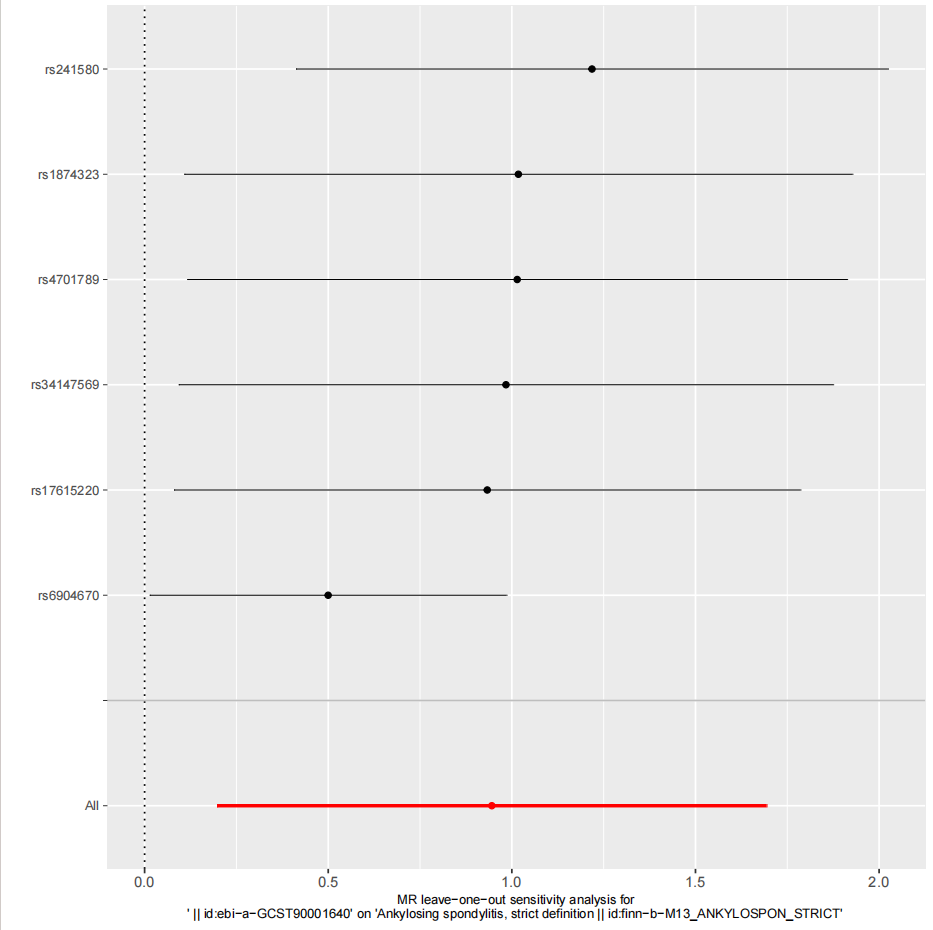


Supplementary Figure S1D Forest plot, funnel plot, scatter plot and sensitivity analysis of SNPs associated with CD3- lymphocyte %lymphocyte on AS.


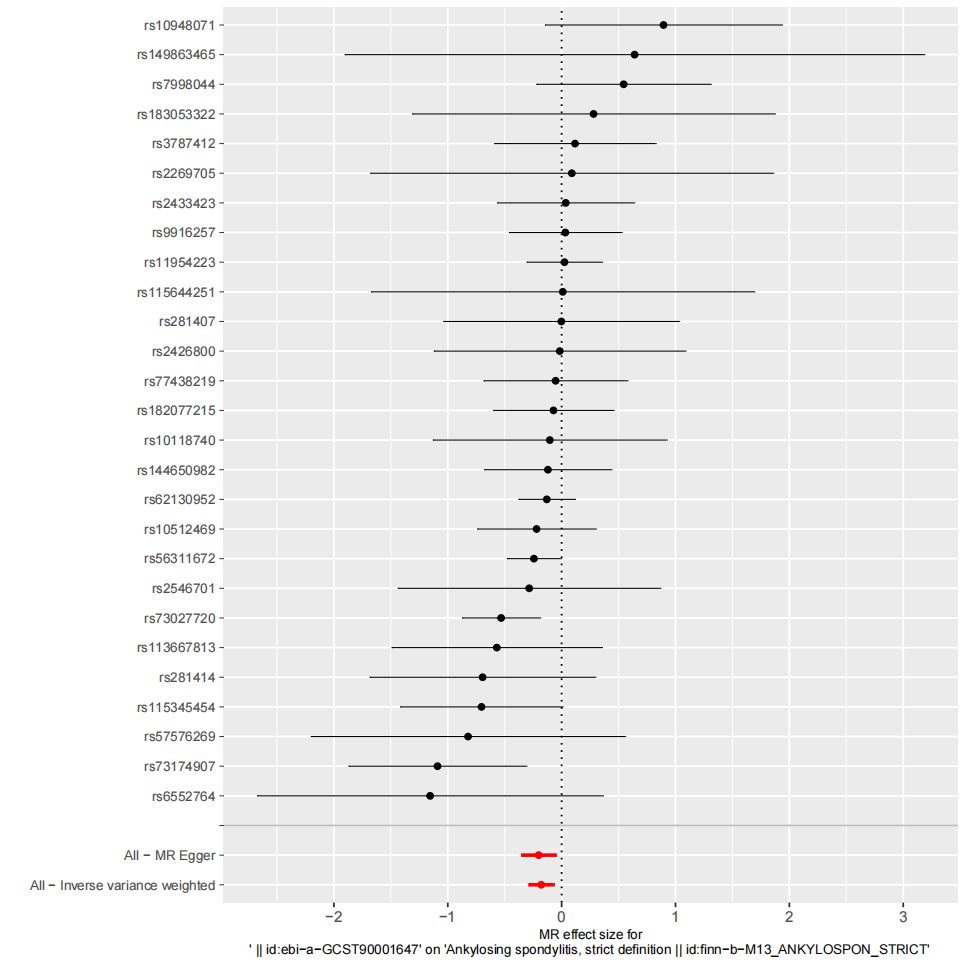

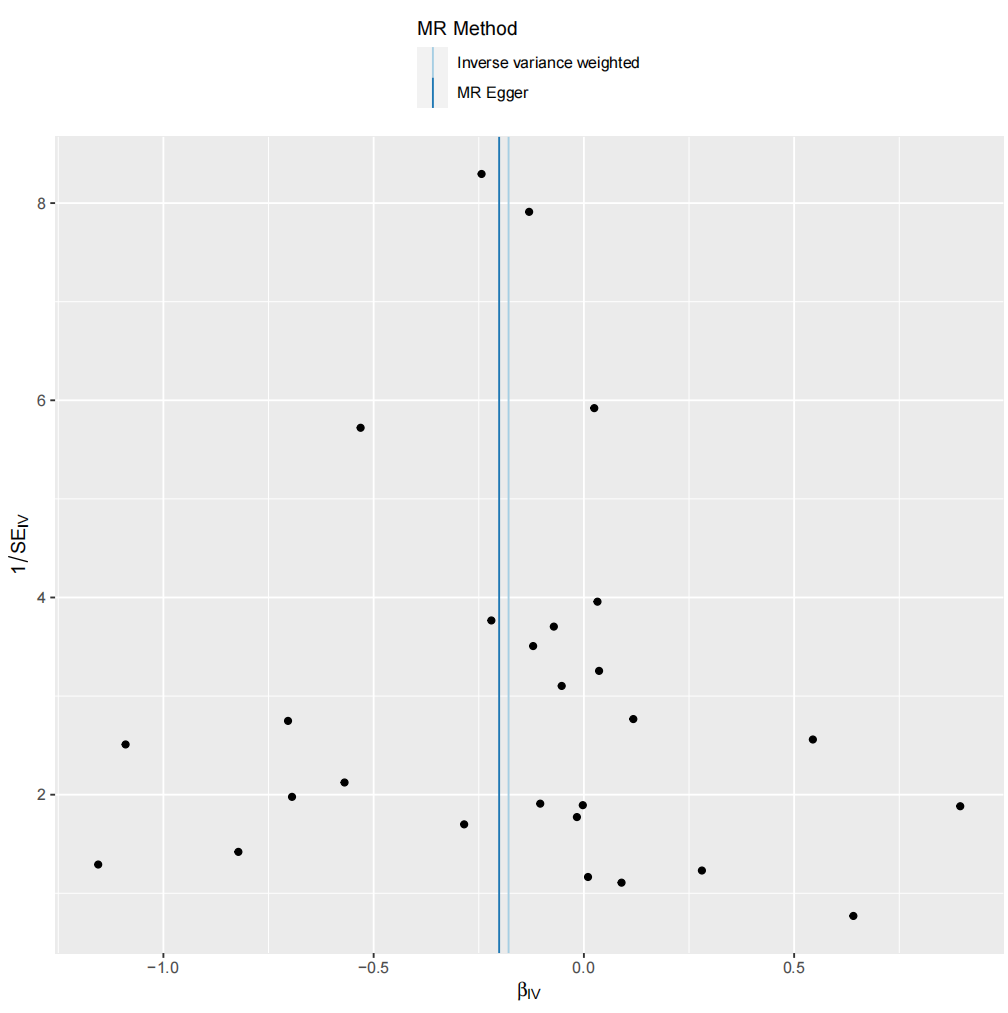

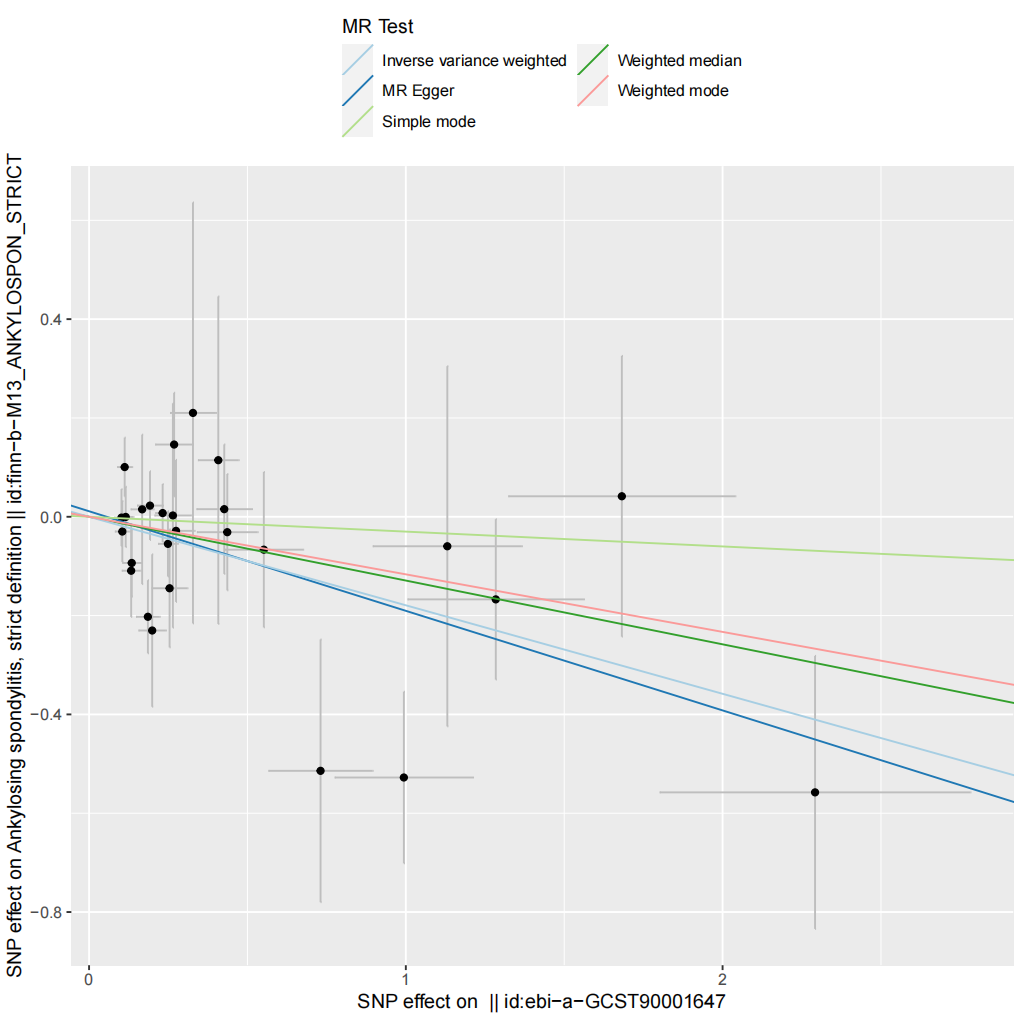

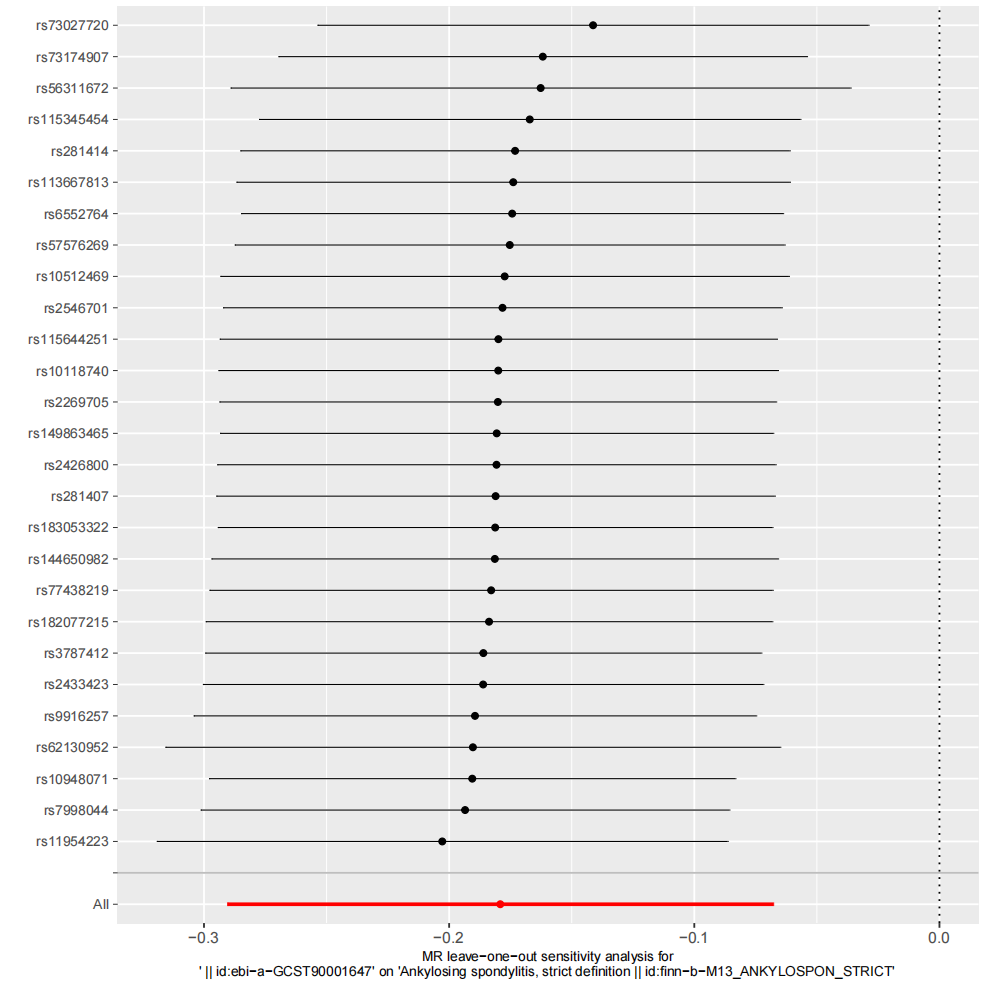


Supplementary Figure S1E Forest plot, funnel plot, scatter plot and sensitivity analysis of SNPs associated with NK %lymphocyte on AS.


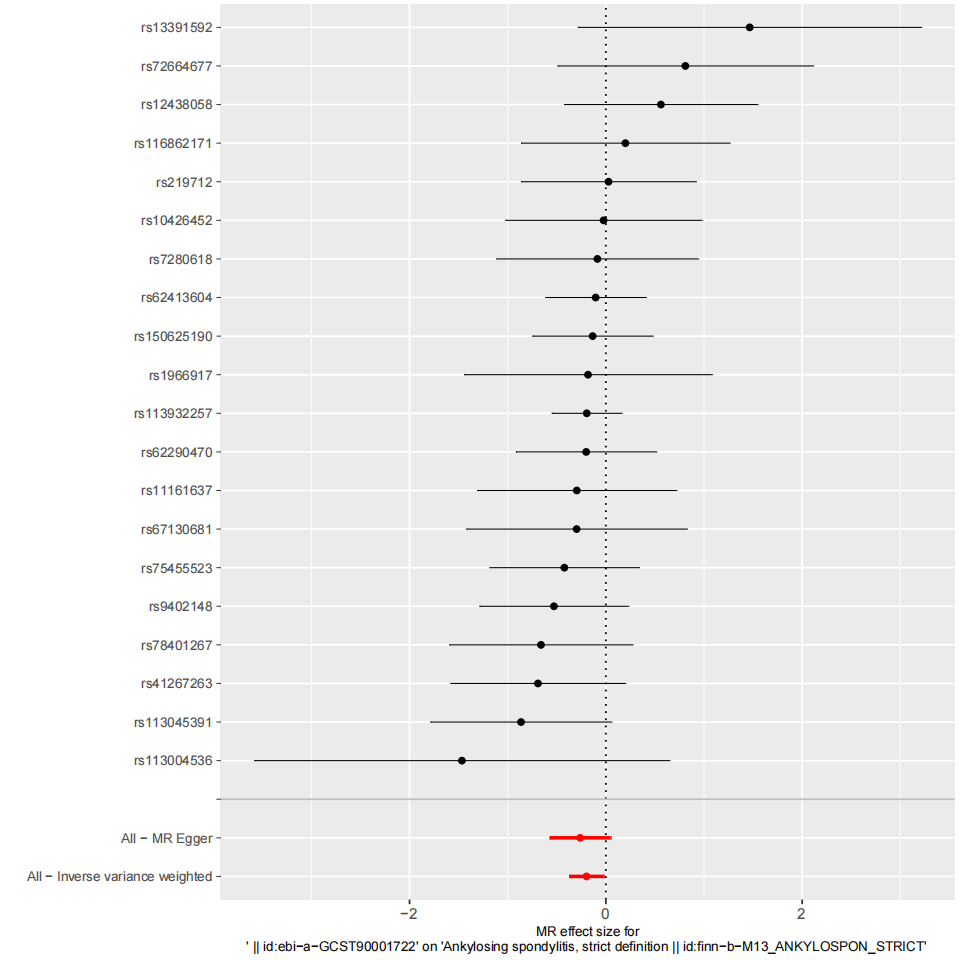

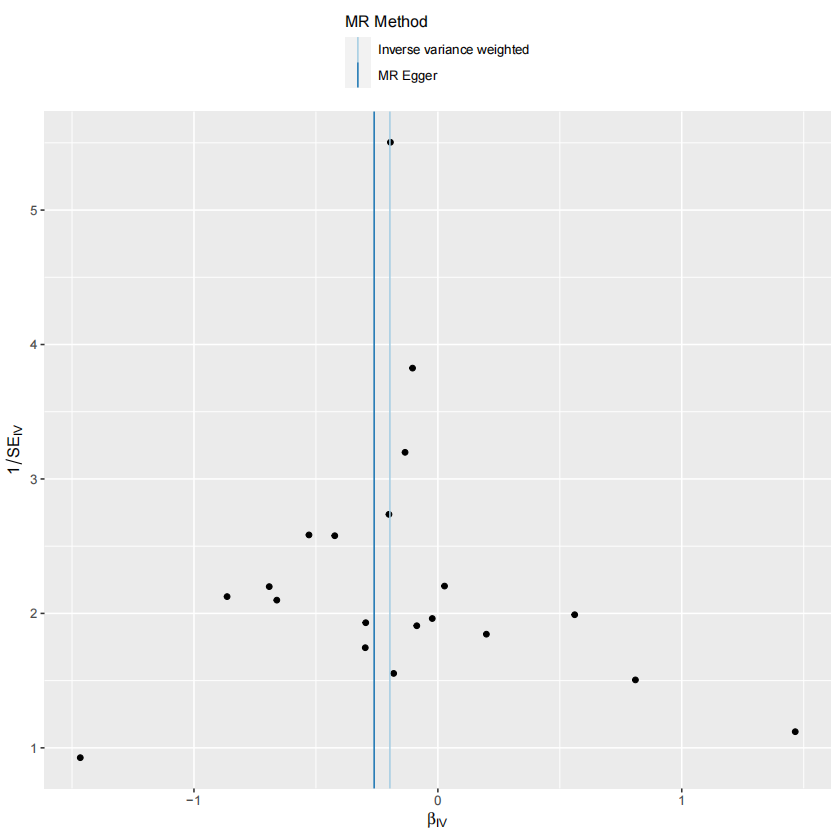

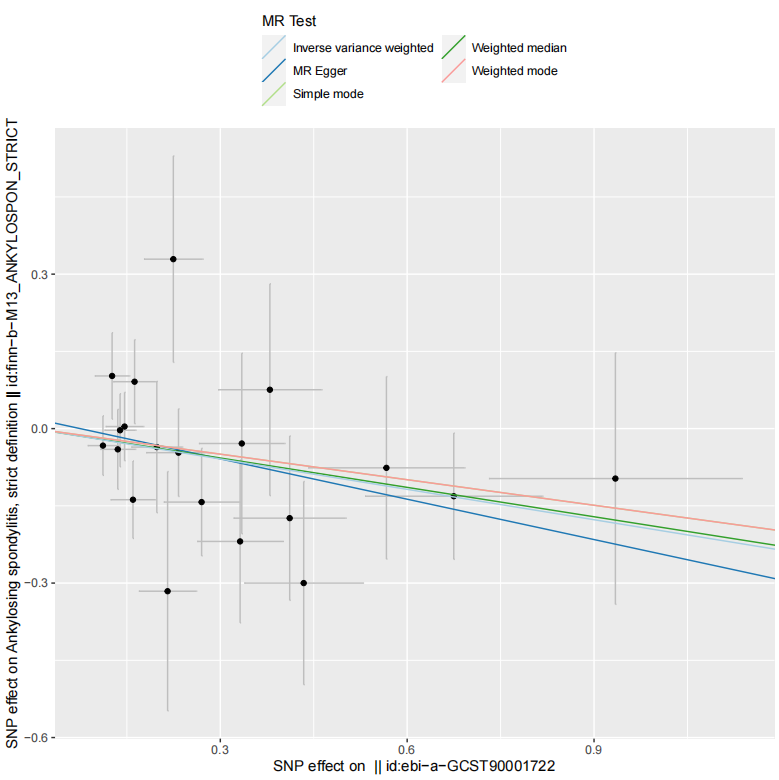

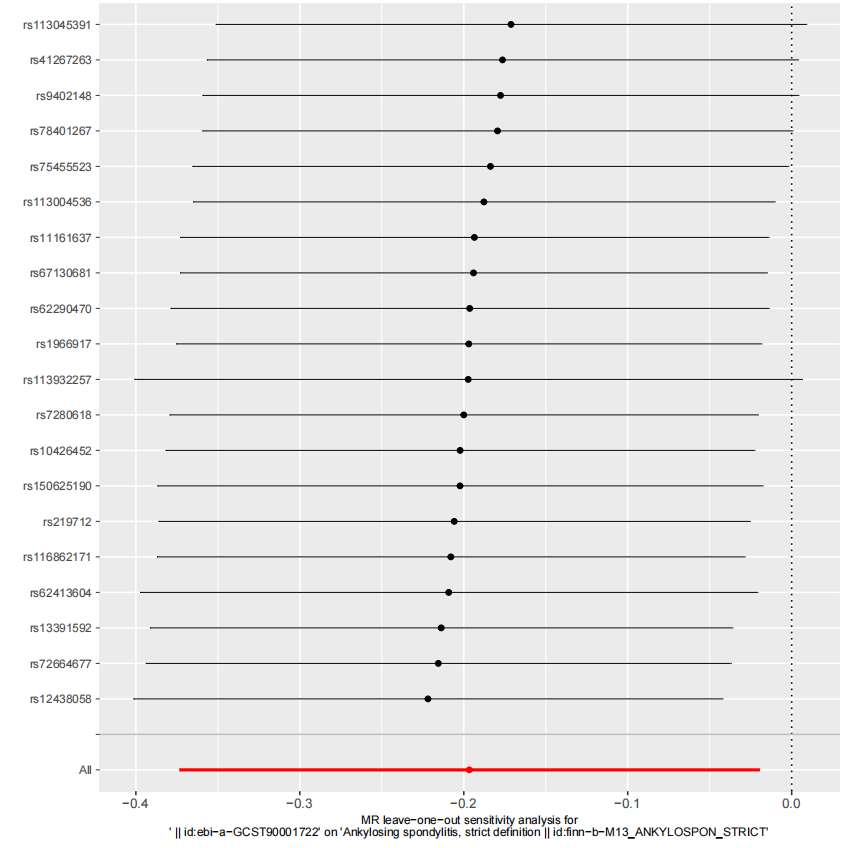


Supplementary Figure S1F Forest plot, funnel plot, scatter plot and sensitivity analysis of SNPs associated with CD19 on CD20- CD38- on AS.


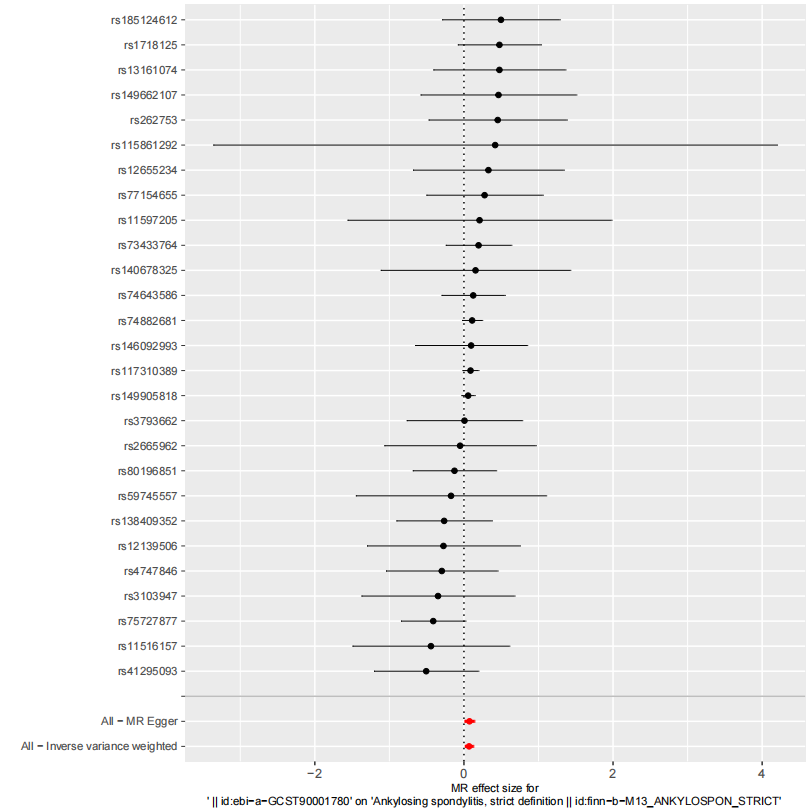

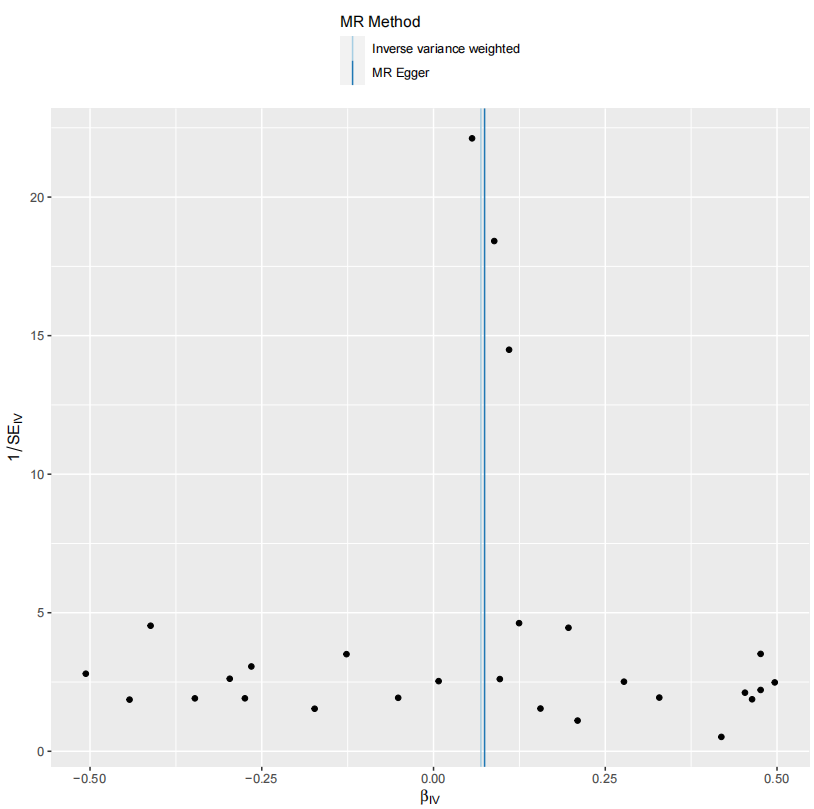

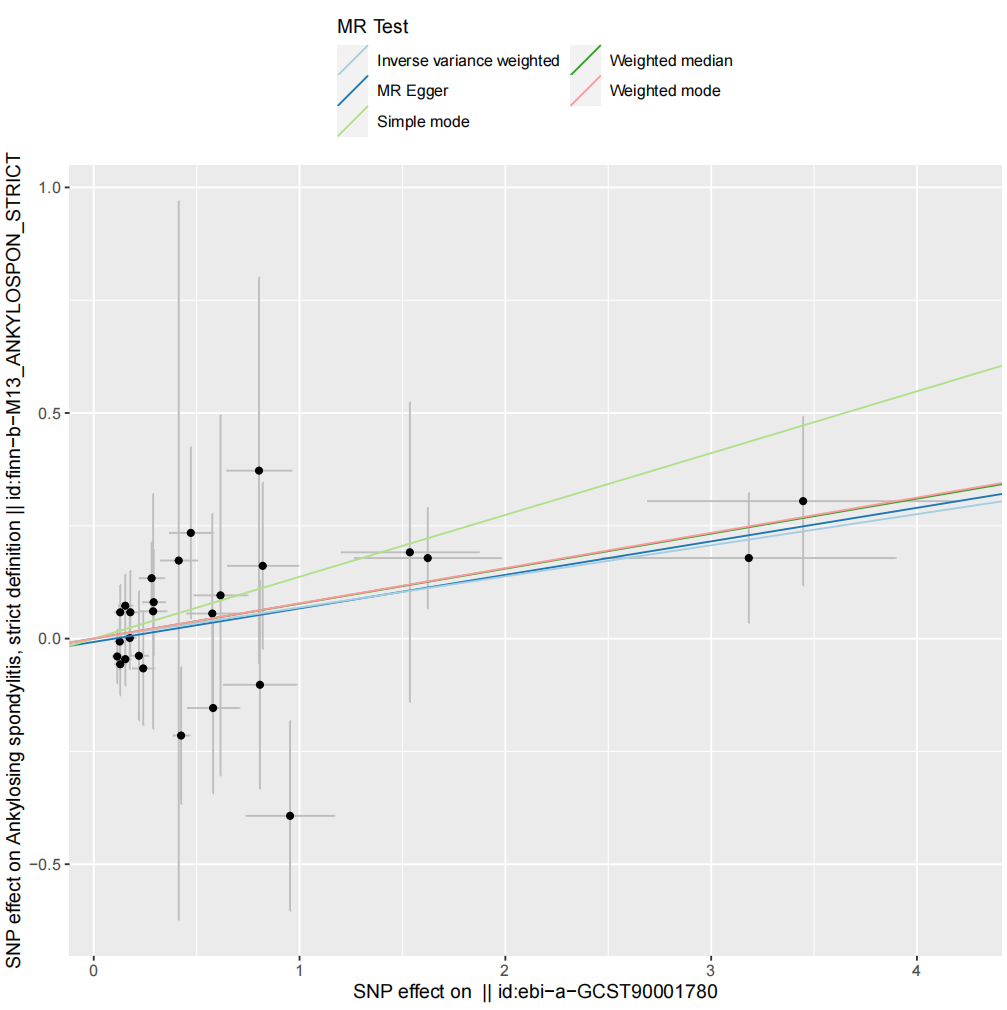

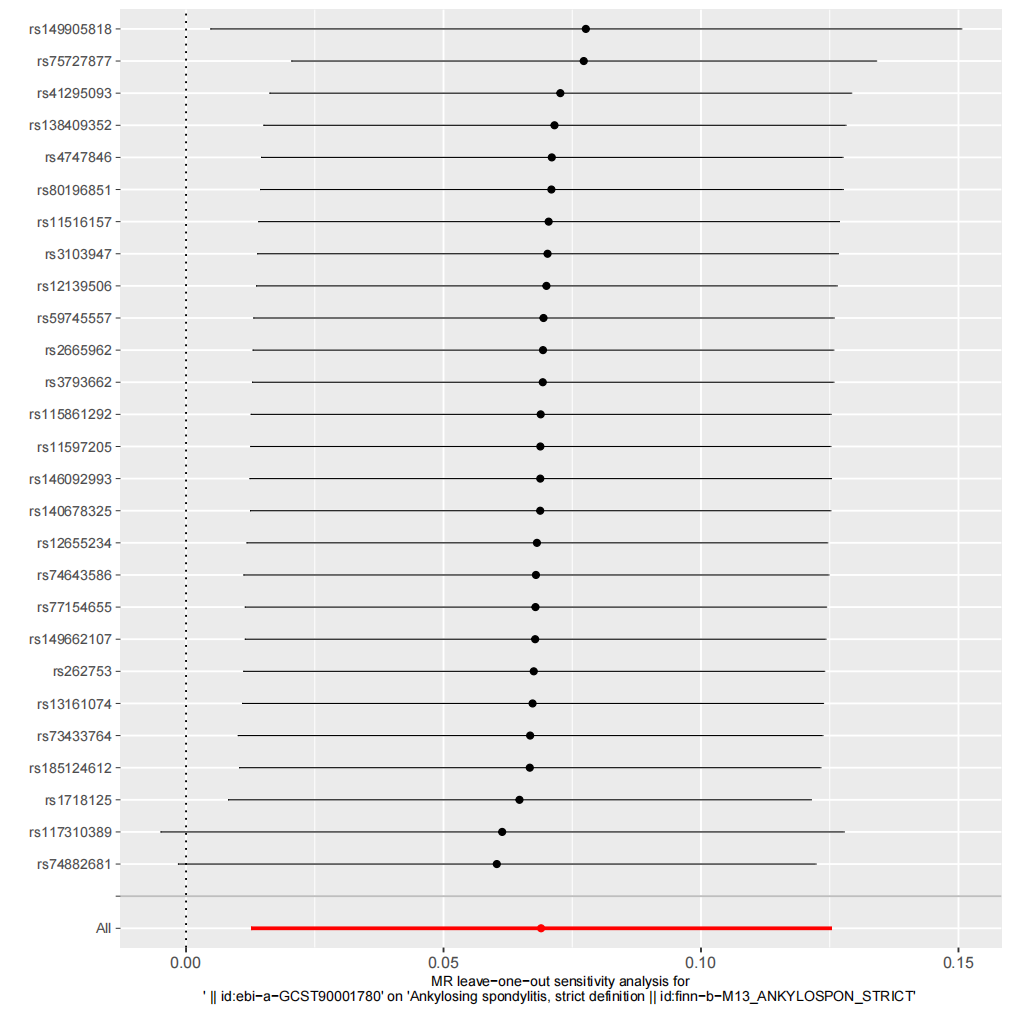


Supplementary Figure S1G Forest plot, funnel plot, scatter plot and sensitivity analysis of SNPs associated with CD25 on IgD+ CD38- on AS.


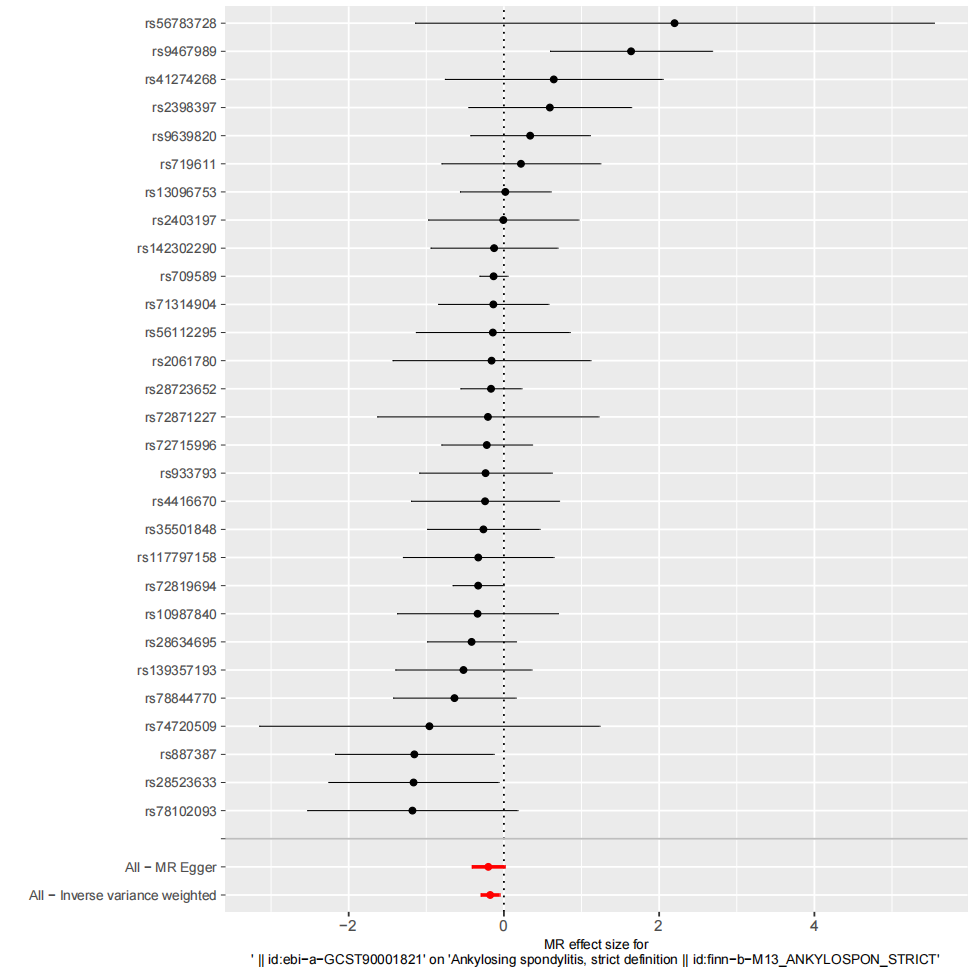

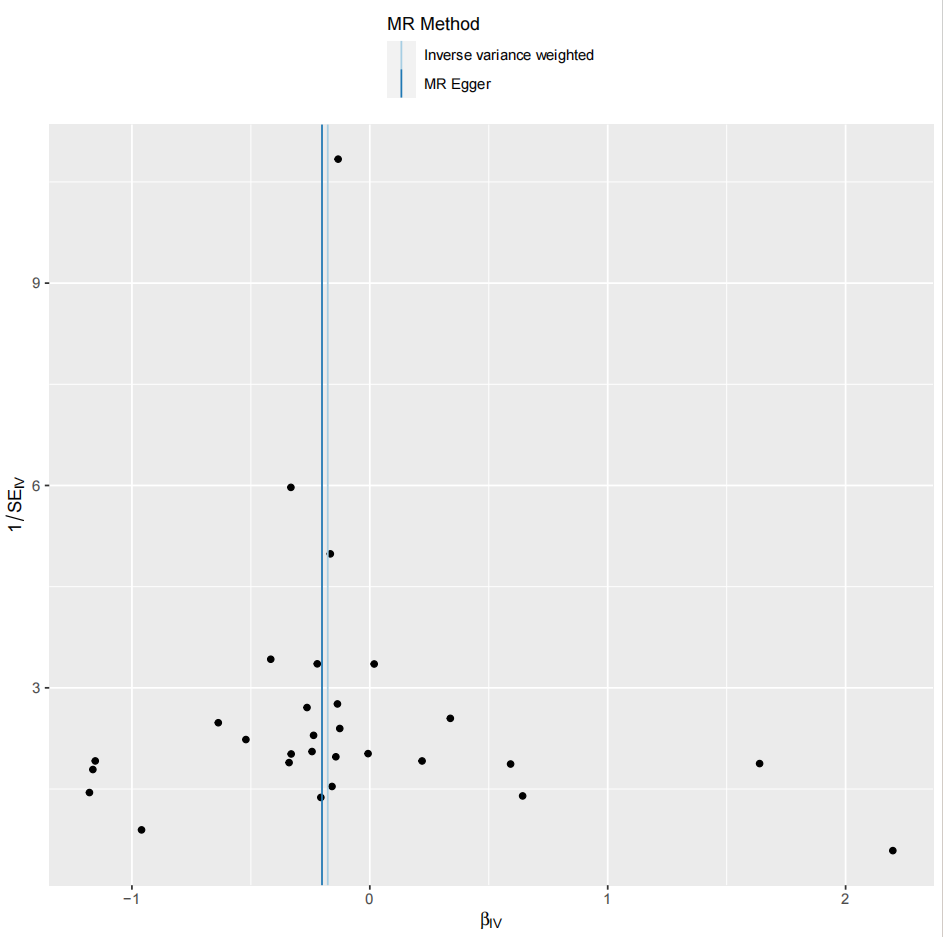

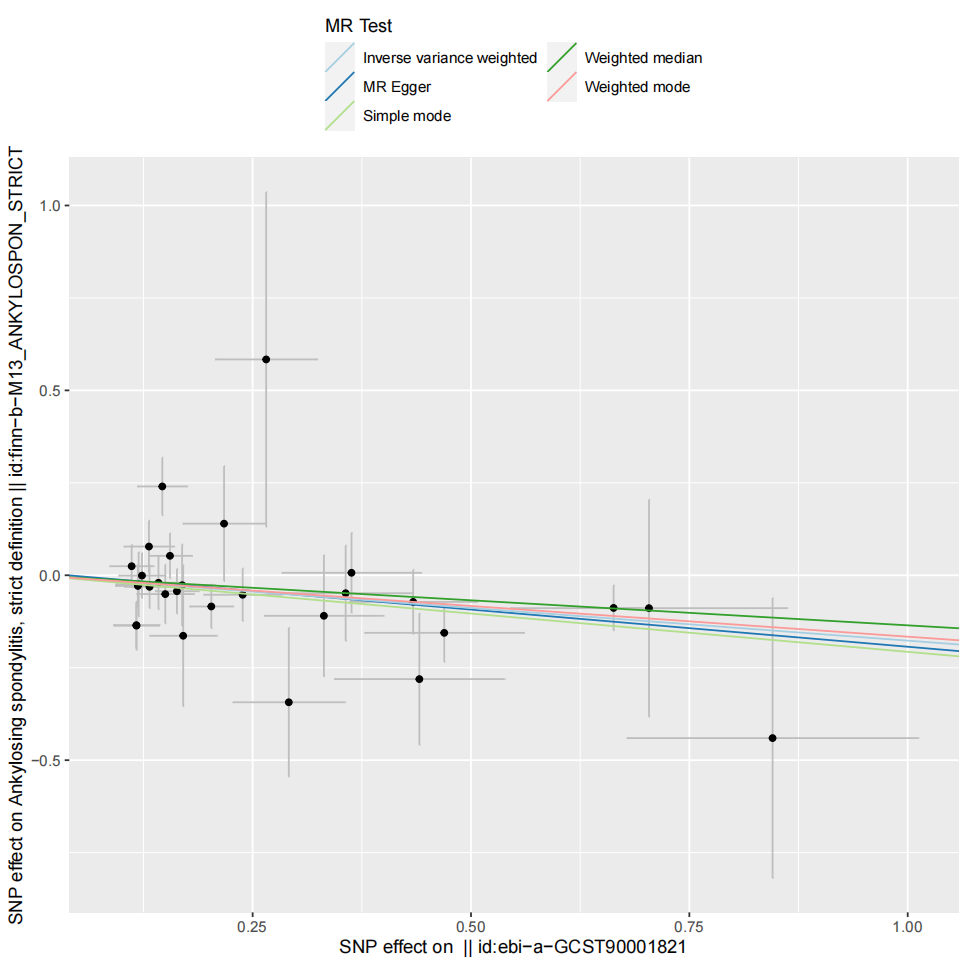

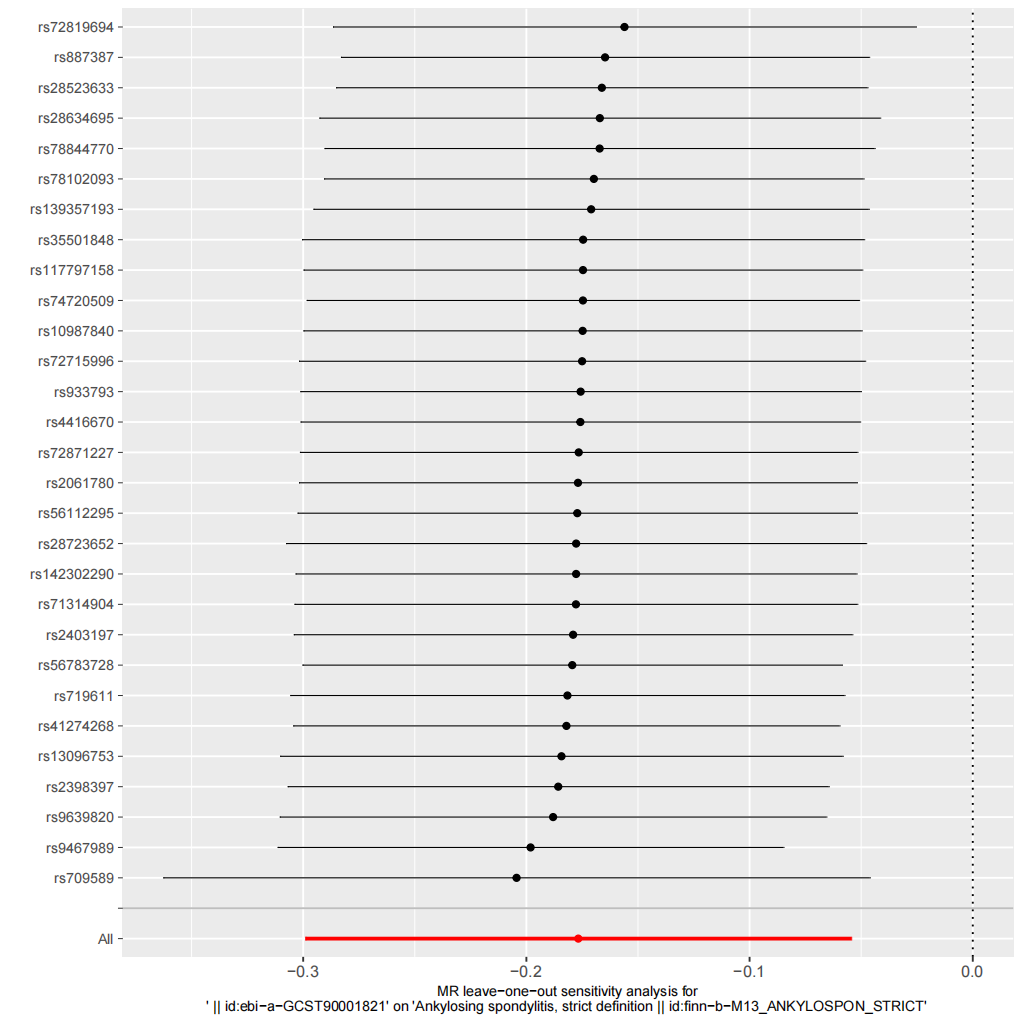


Supplementary Figure S1H Forest plot, funnel plot, scatter plot and sensitivity analysis of SNPs associated with IgD on IgD+ CD24- on AS.


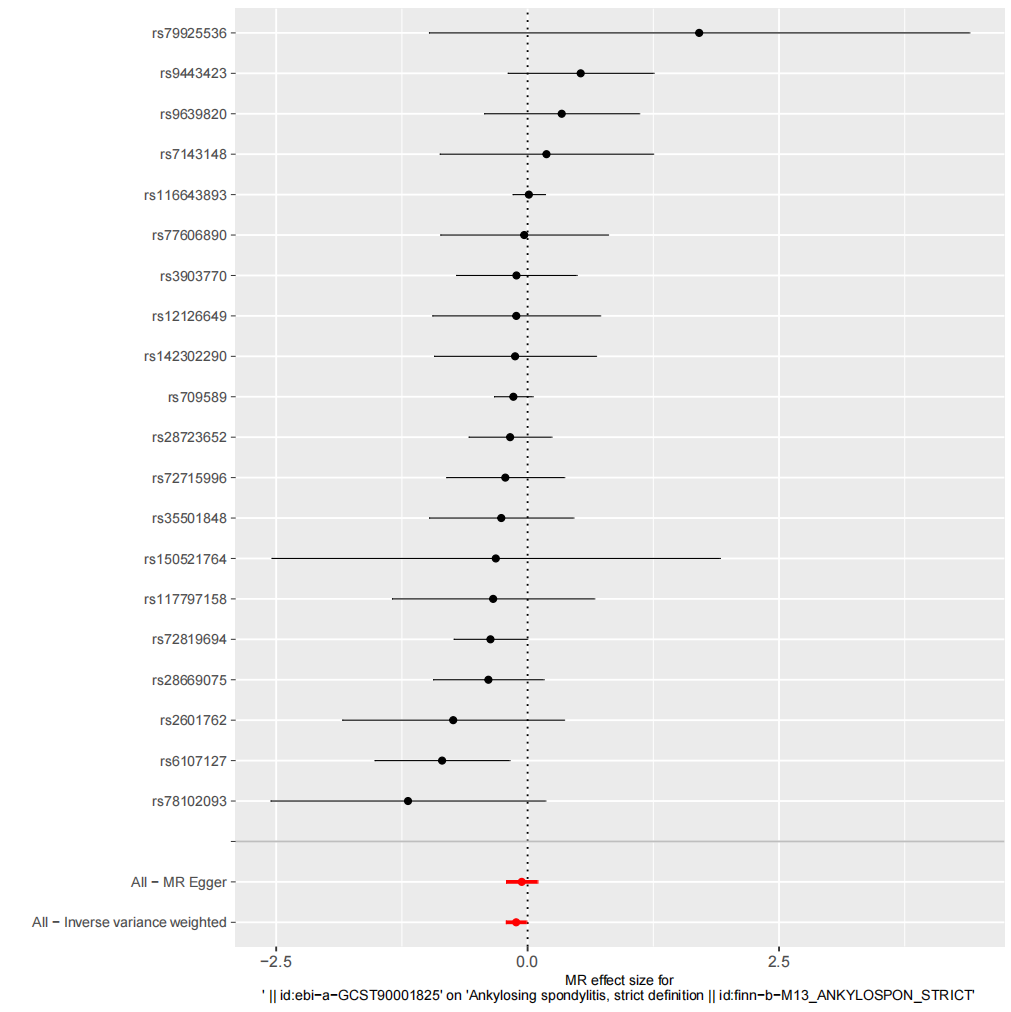

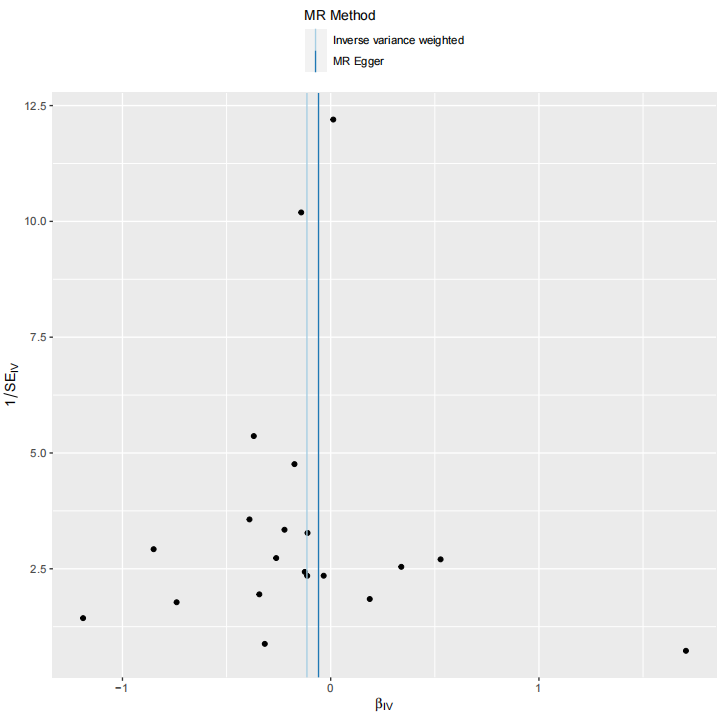

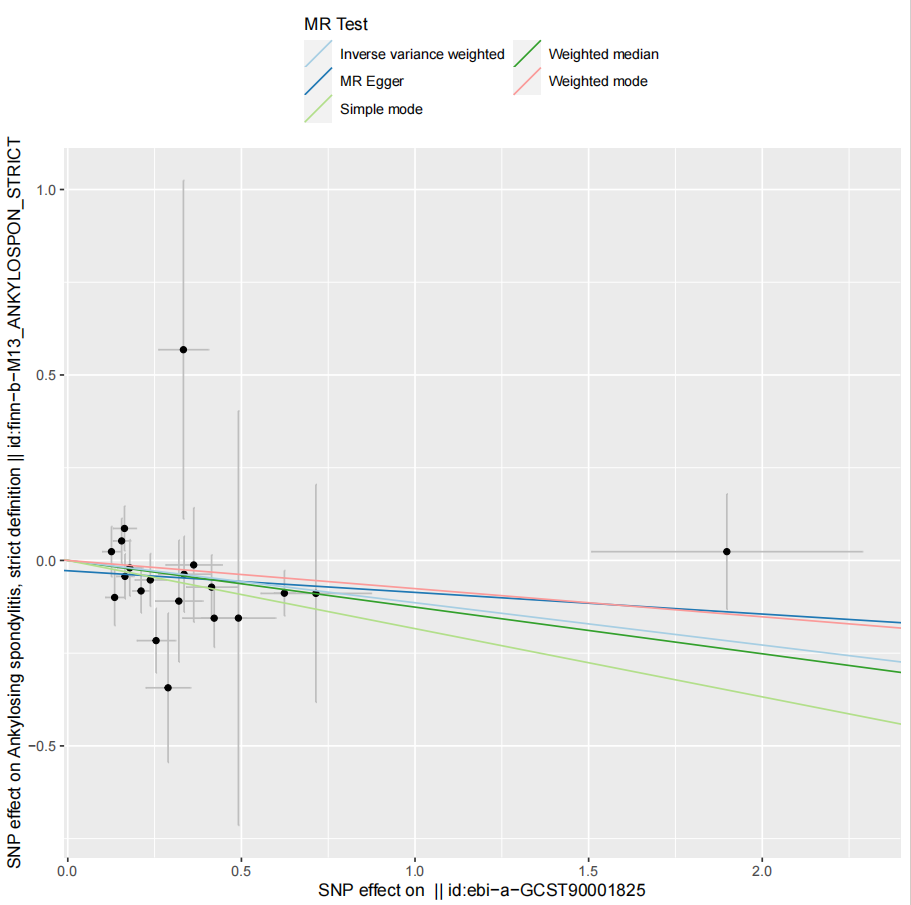

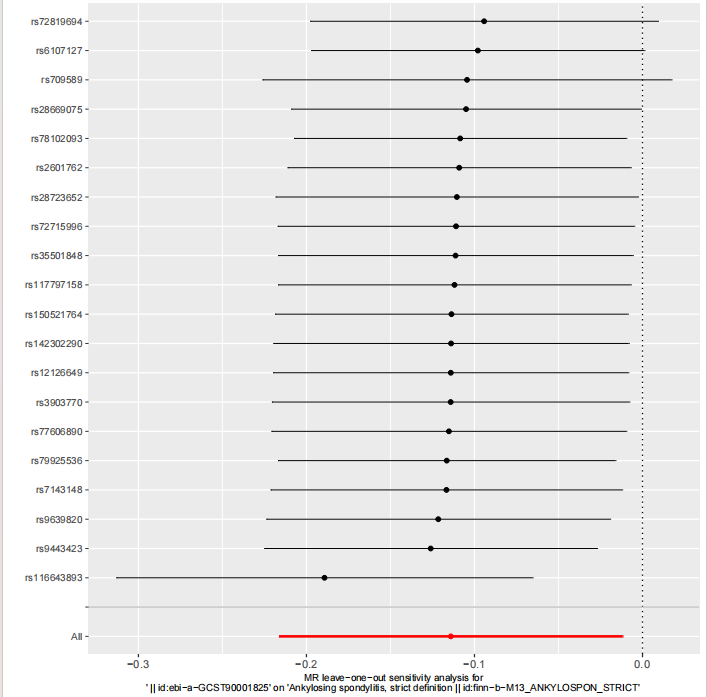


Supplementary Figure S1I Forest plot, funnel plot, scatter plot and sensitivity analysis of SNPs associated with IgD on IgD+ CD38dim on AS.


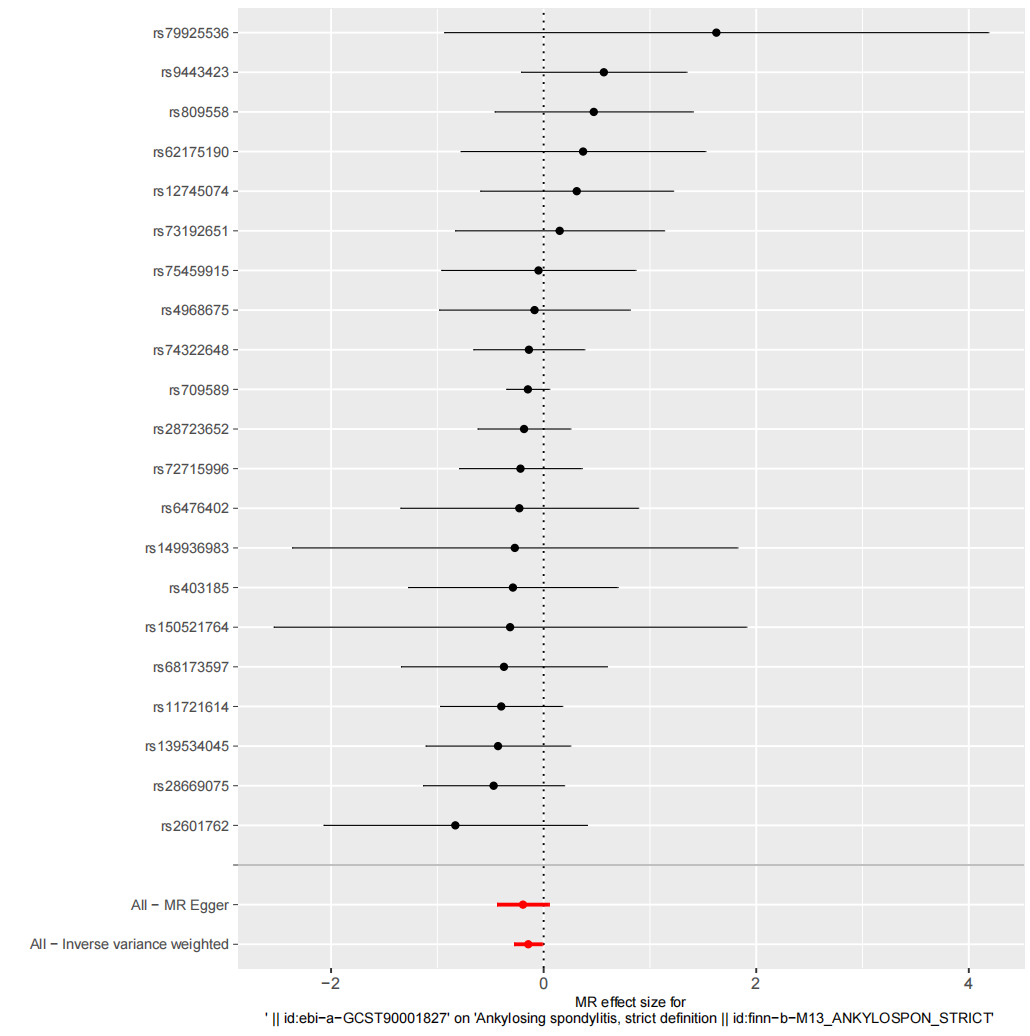

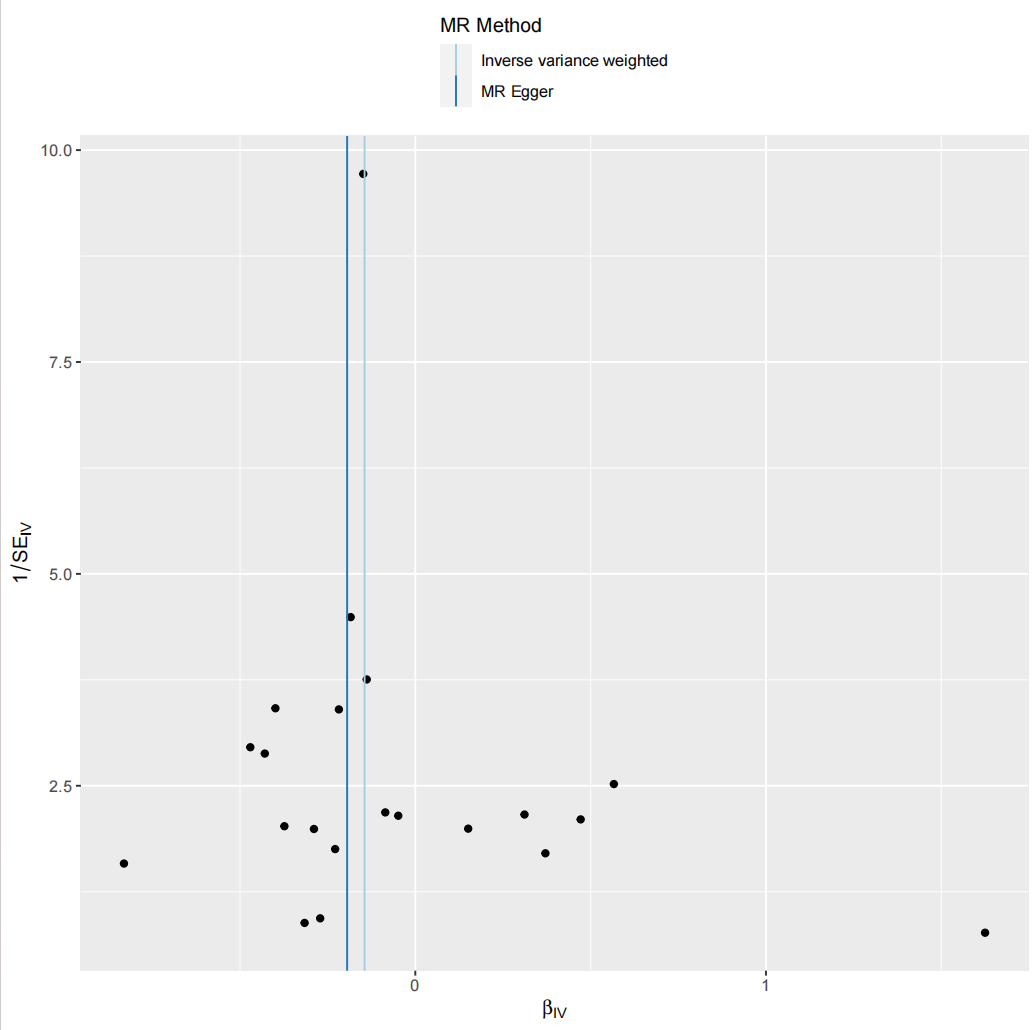

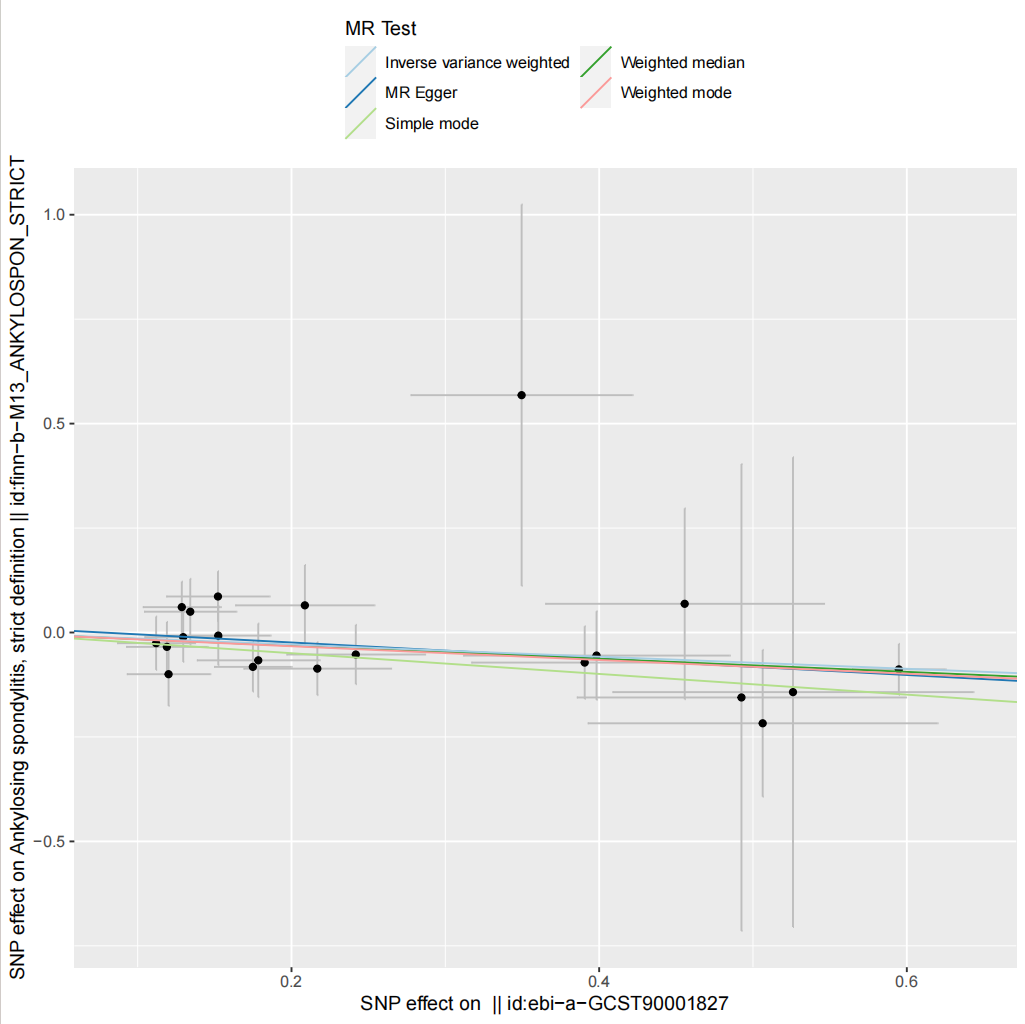

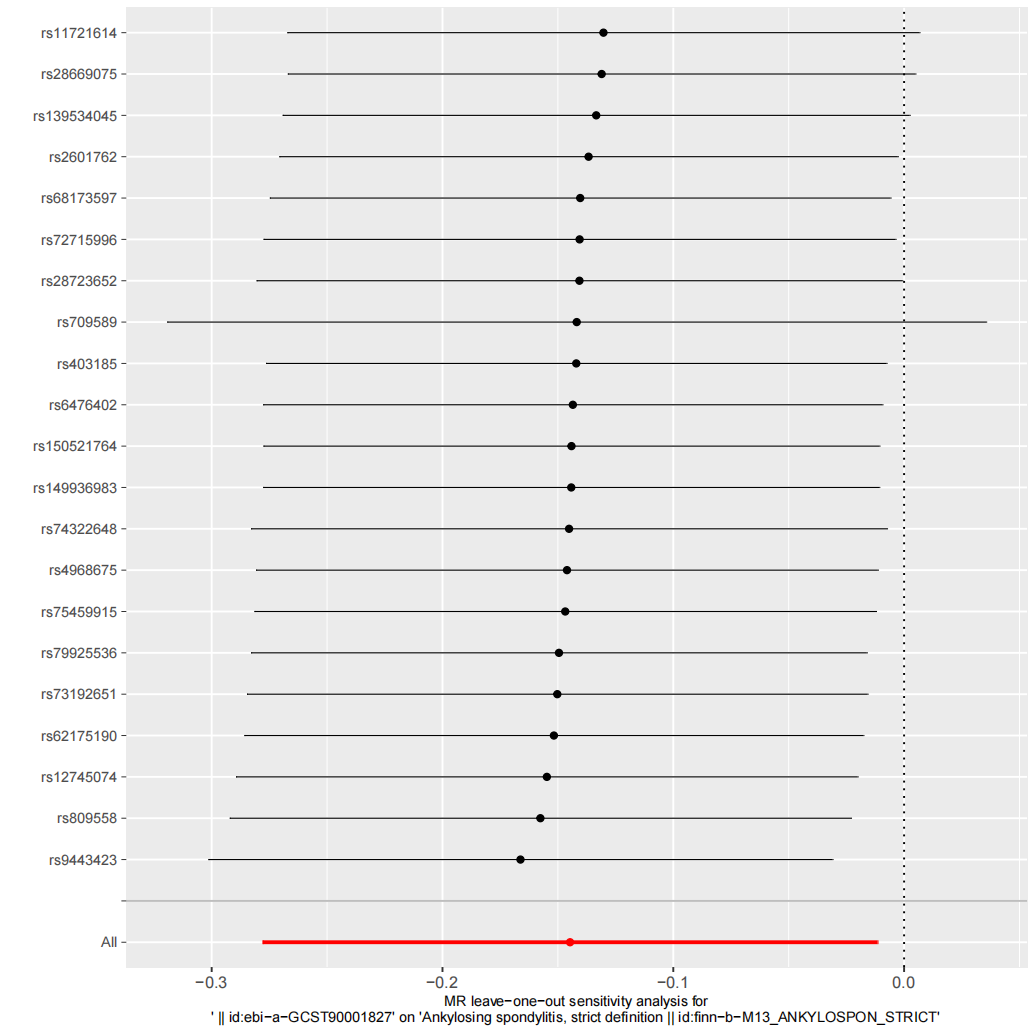


Supplementary Figure S1J Forest plot, funnel plot, scatter plot and sensitivity analysis of SNPs associated with IgD on IgD+ on AS.


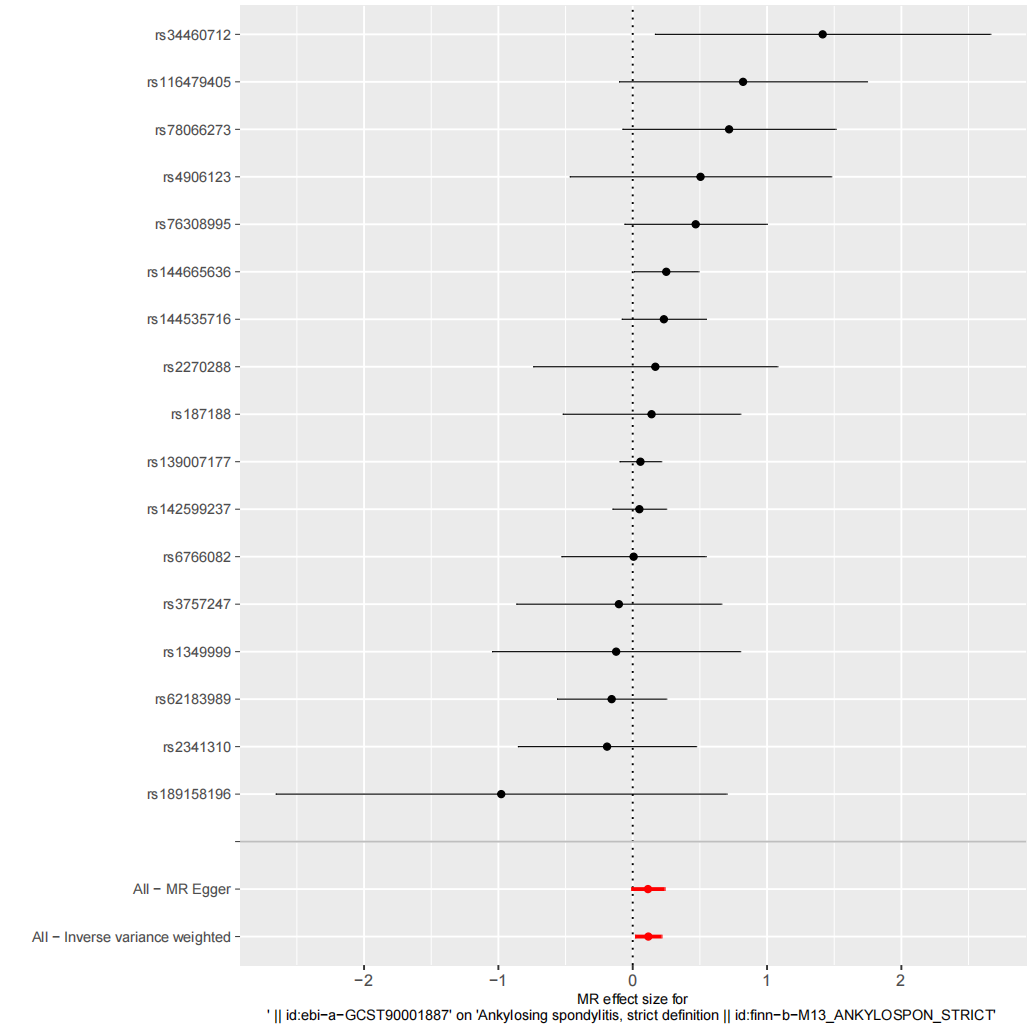

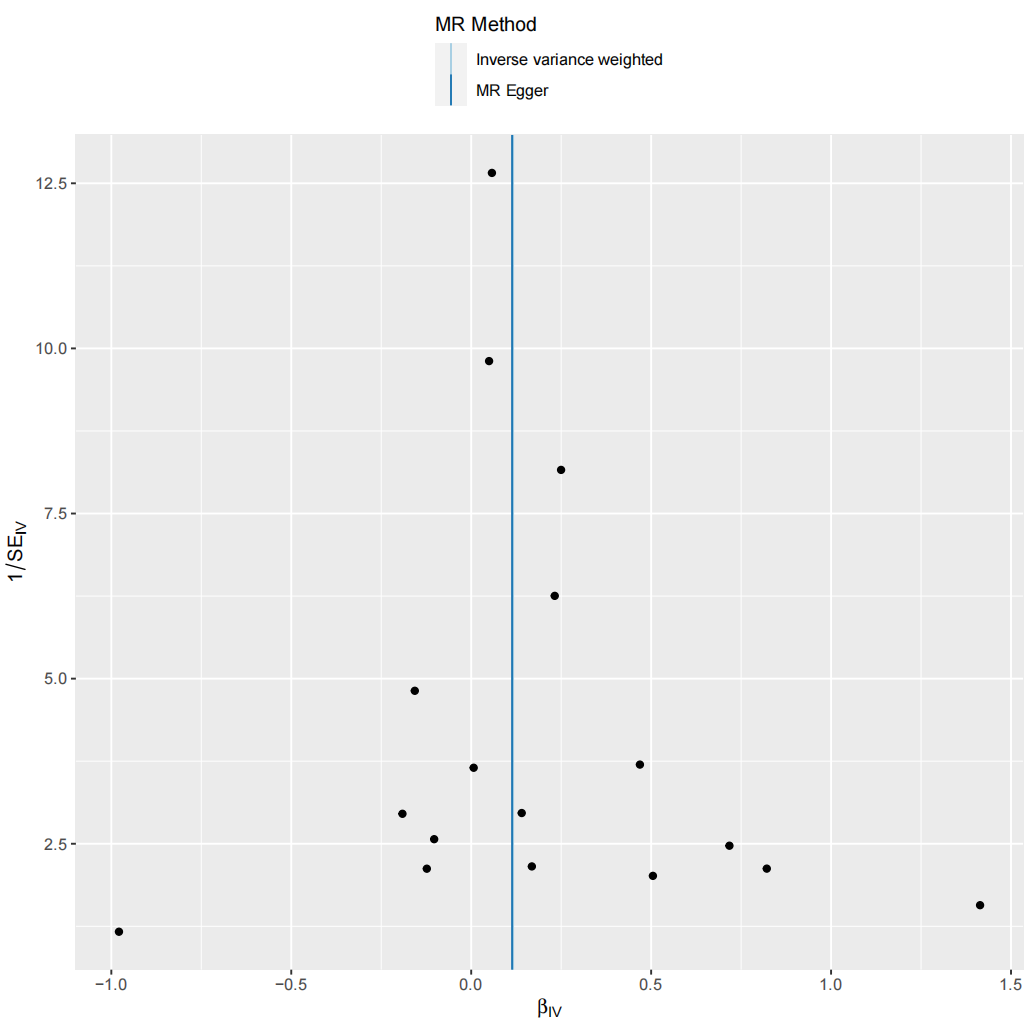


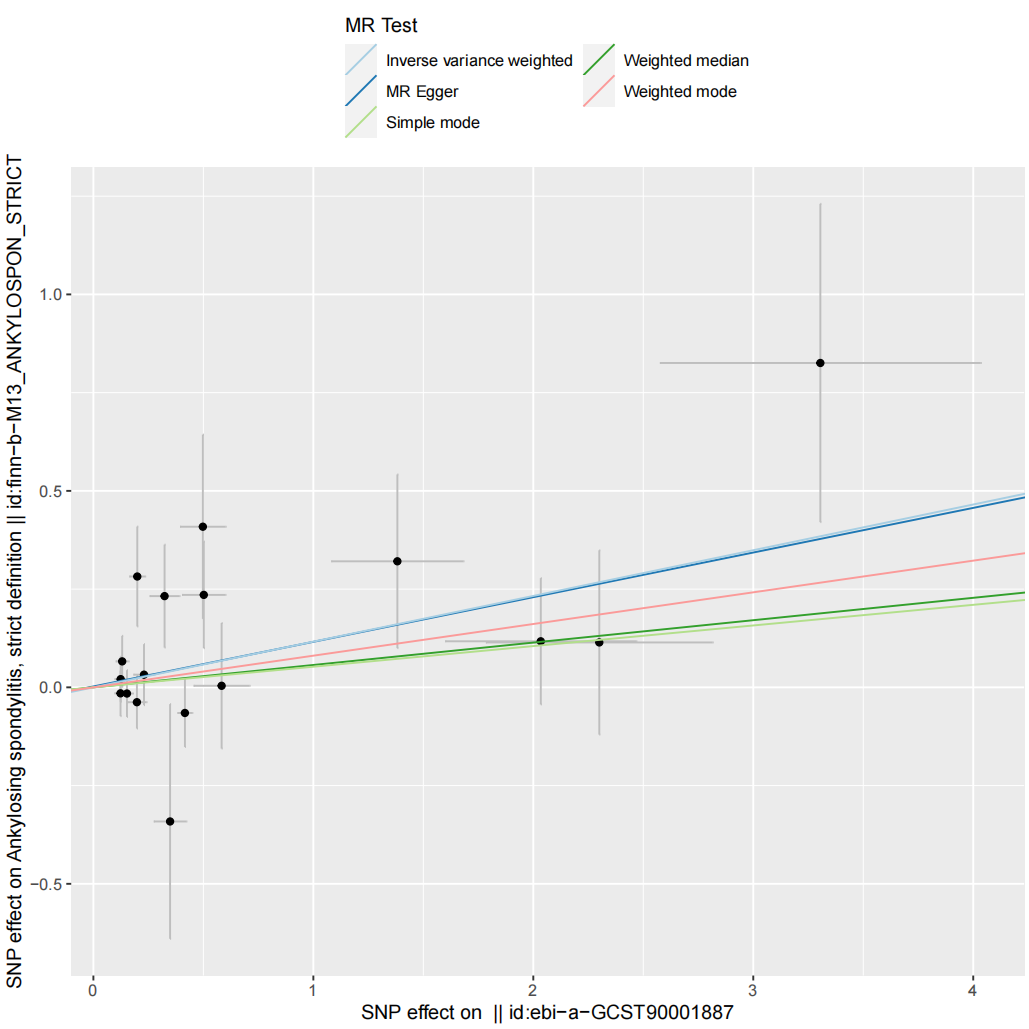

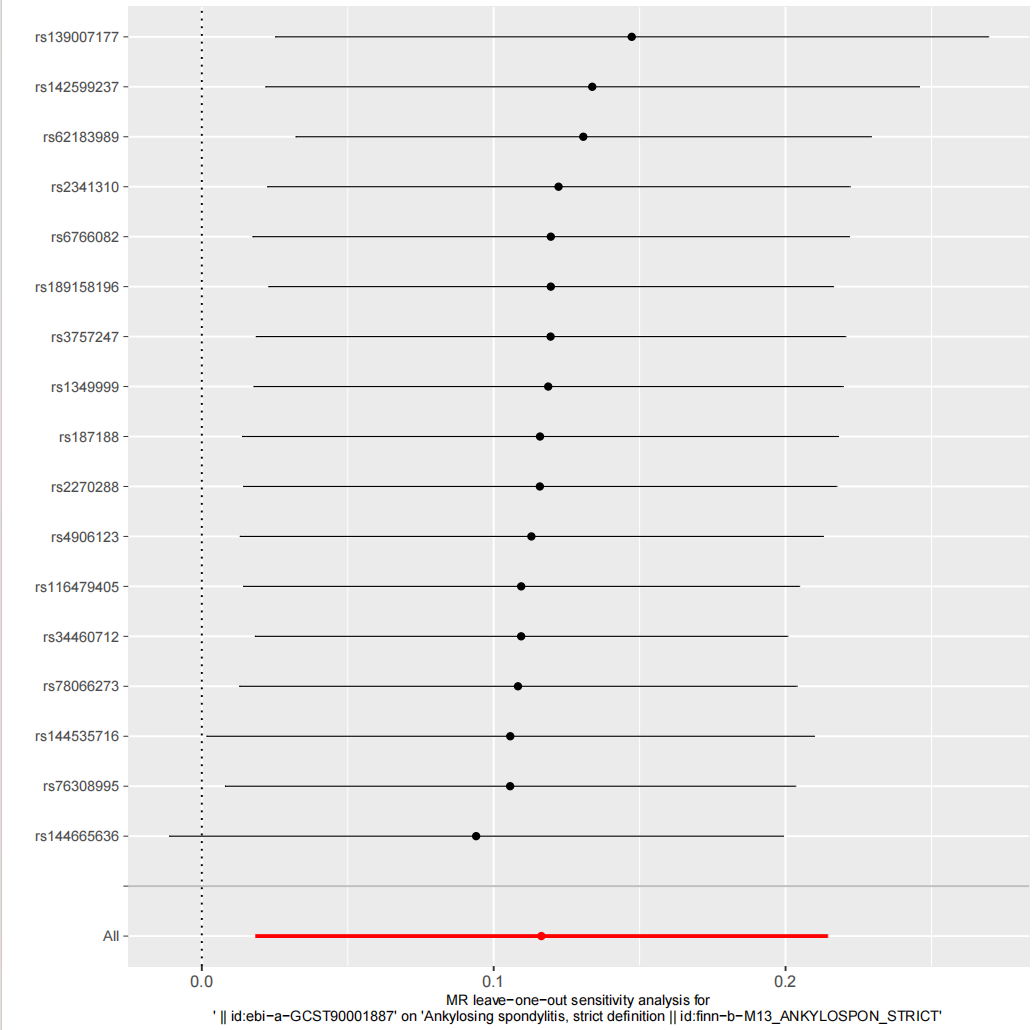


Supplementary Figure S1K Forest plot, funnel plot, scatter plot and sensitivity analysis of SNPs associated with CD28 on secreting Treg on AS.


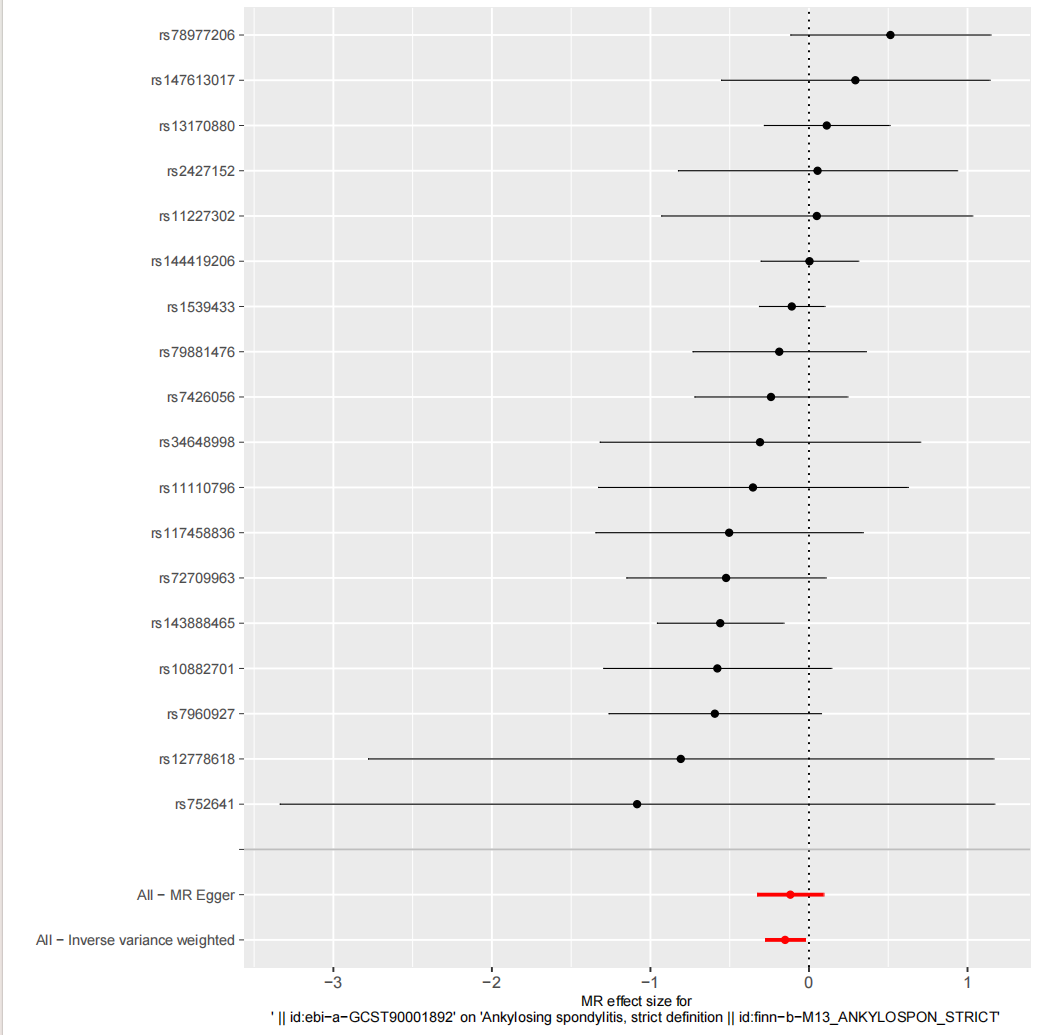

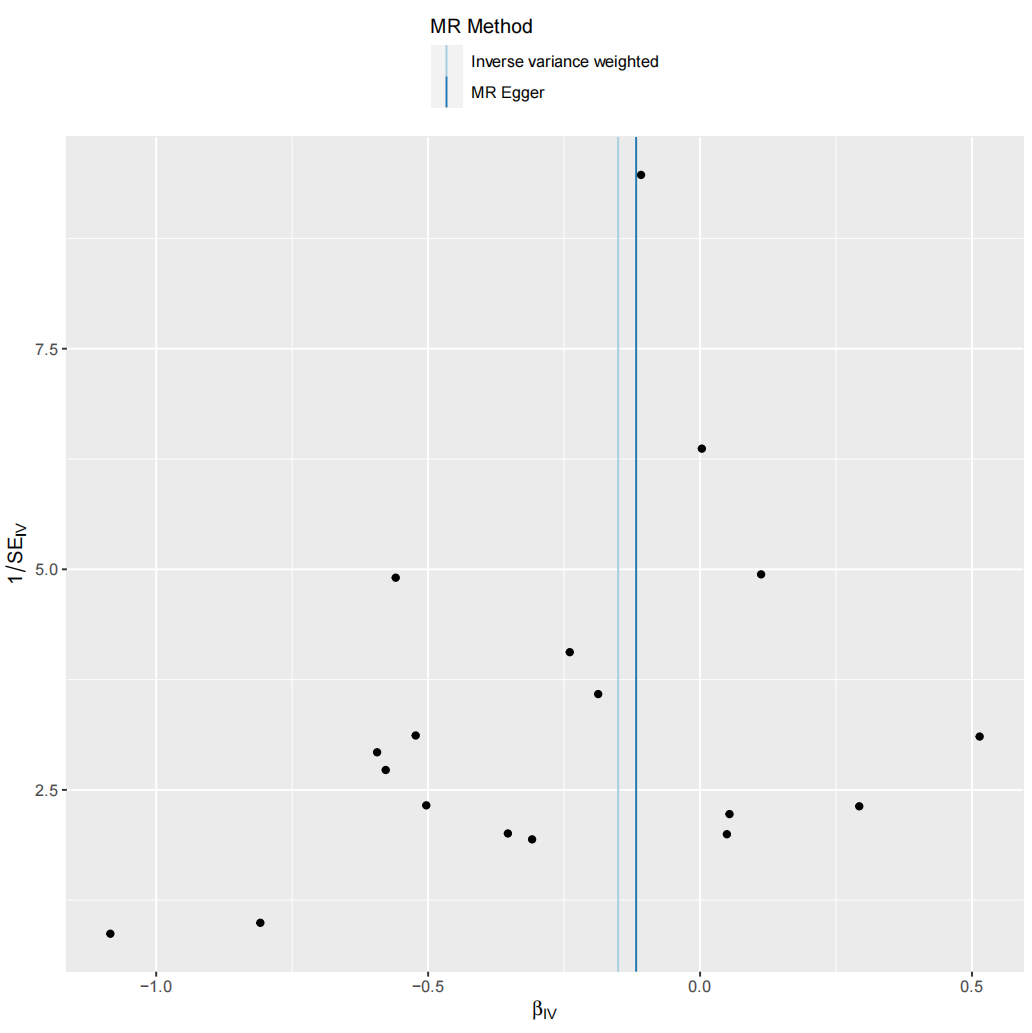

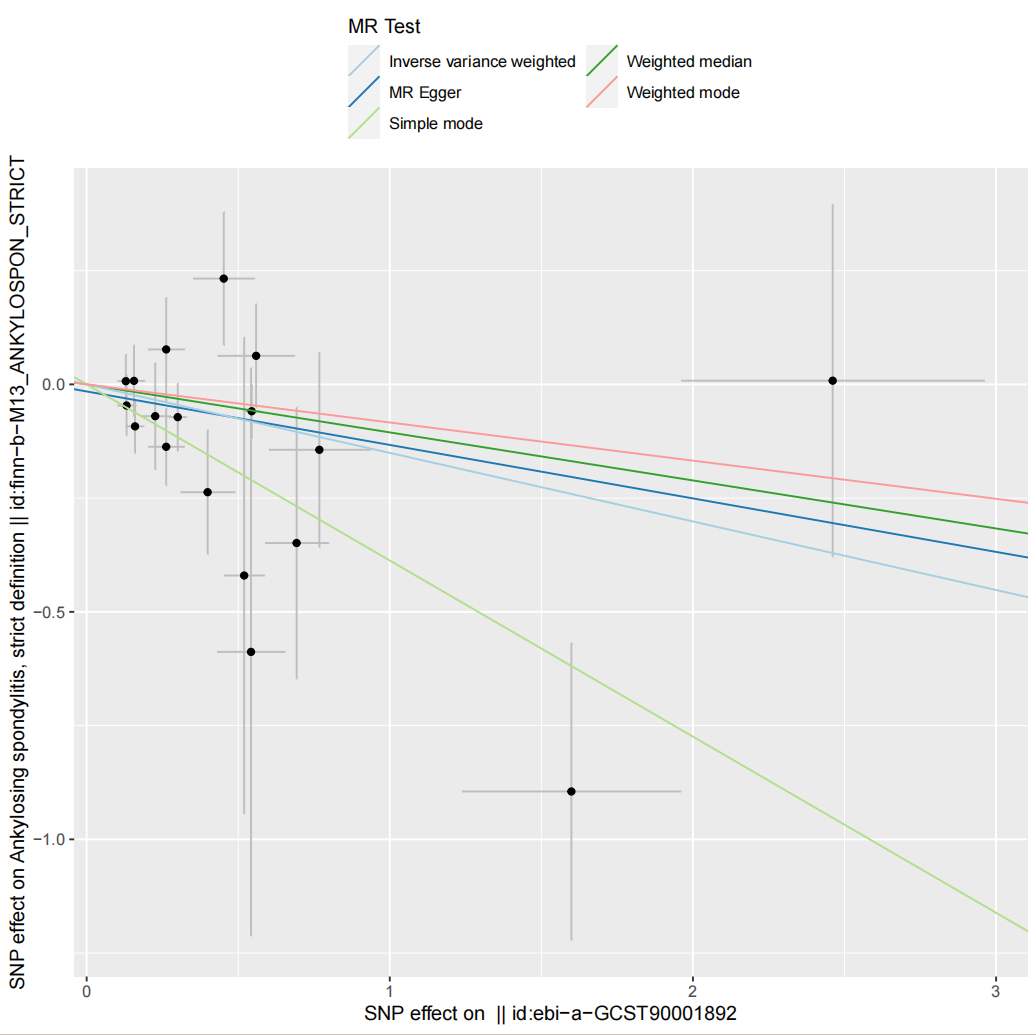

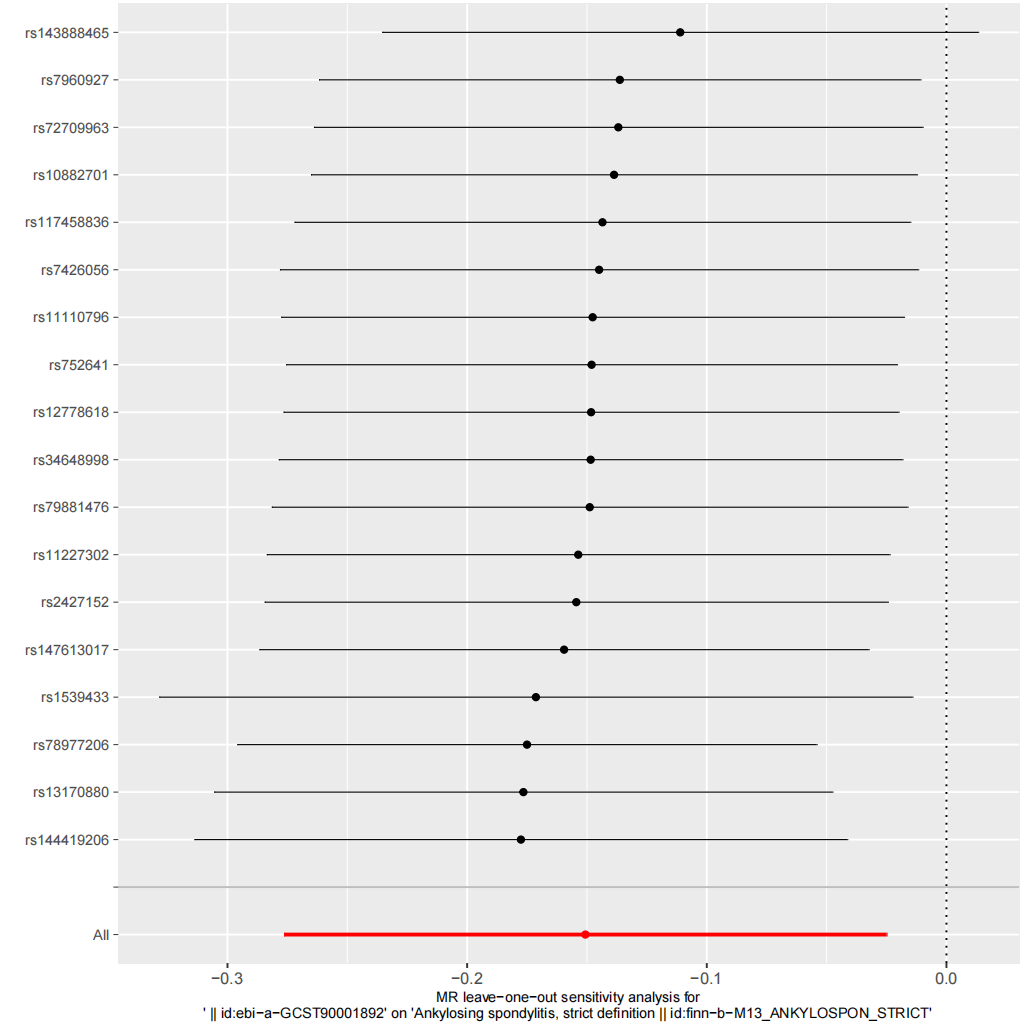
 Supplementary Figure S1L Forest plot, funnel plot, scatter plot and sensitivity analysis of SNPs associated withCD28 on CD39+ CD4+ on AS.


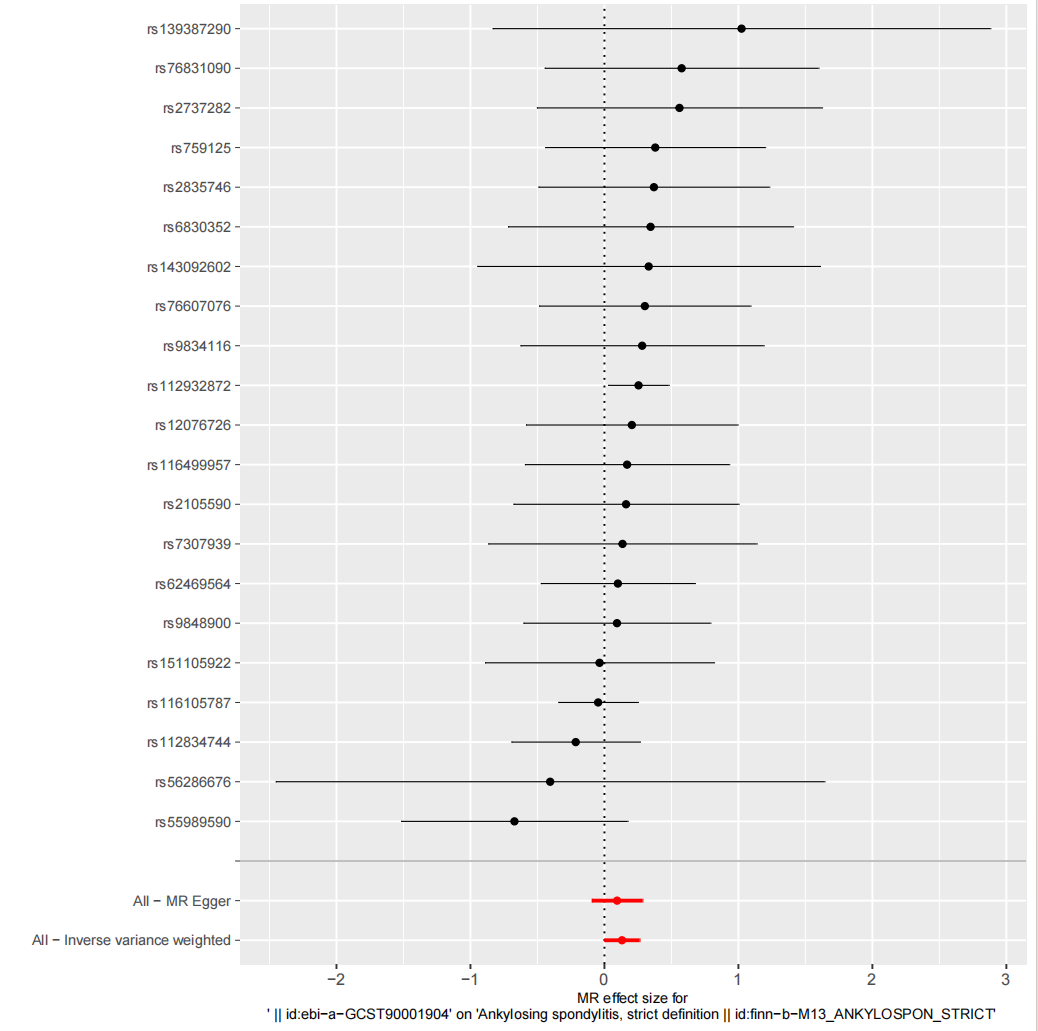

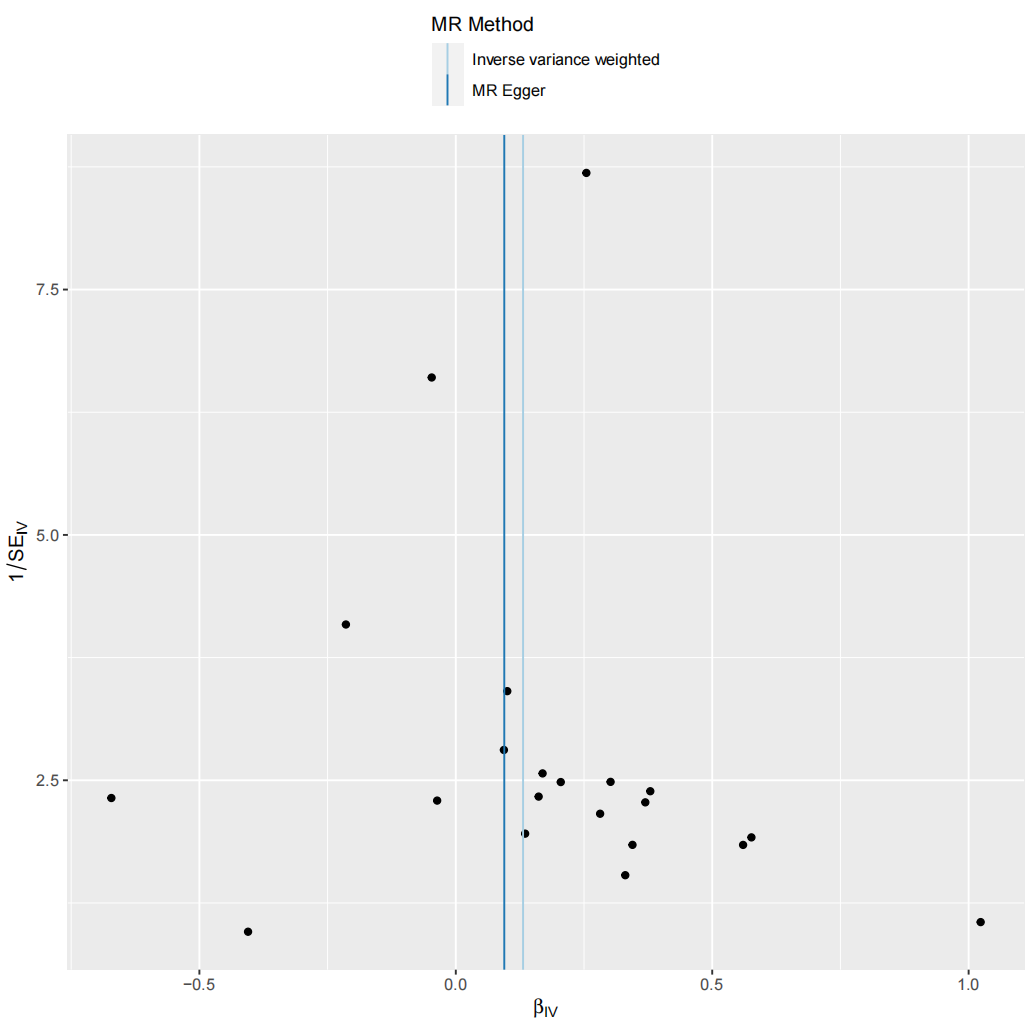


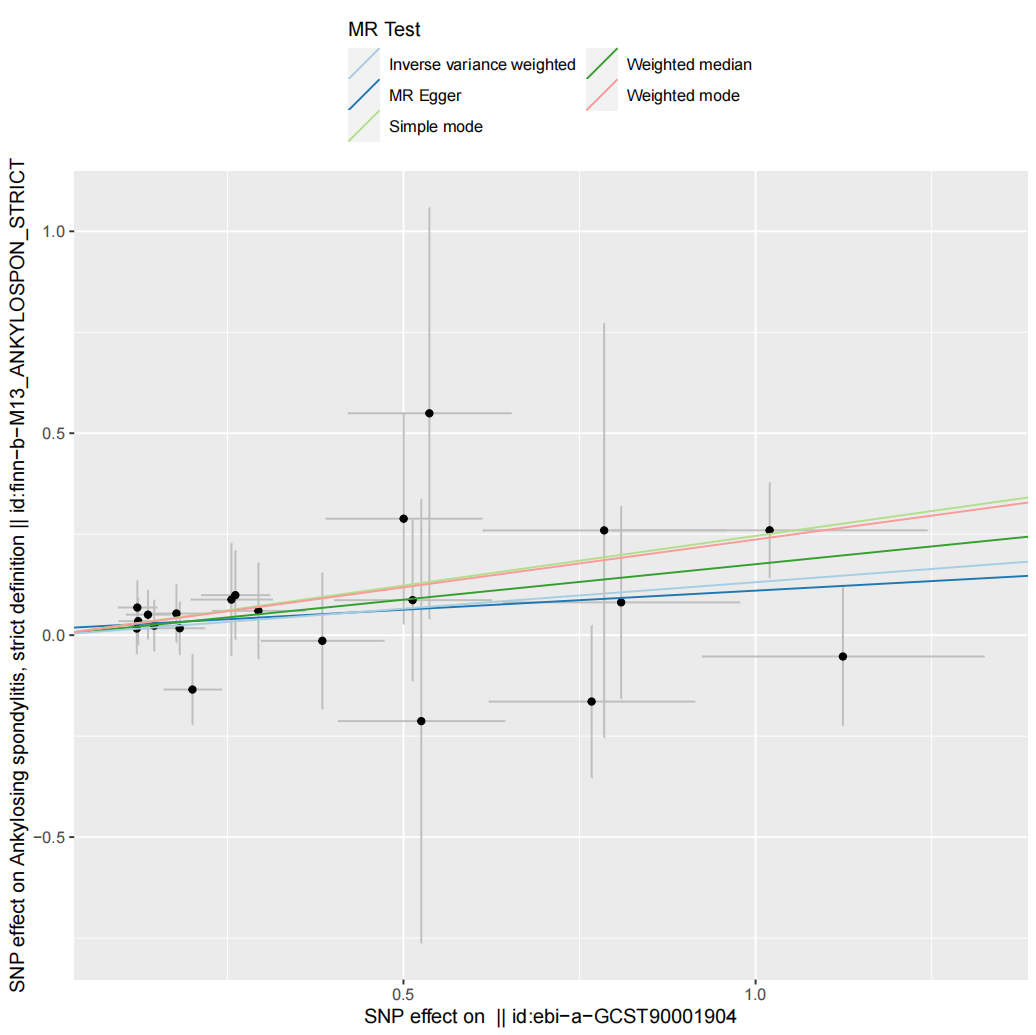

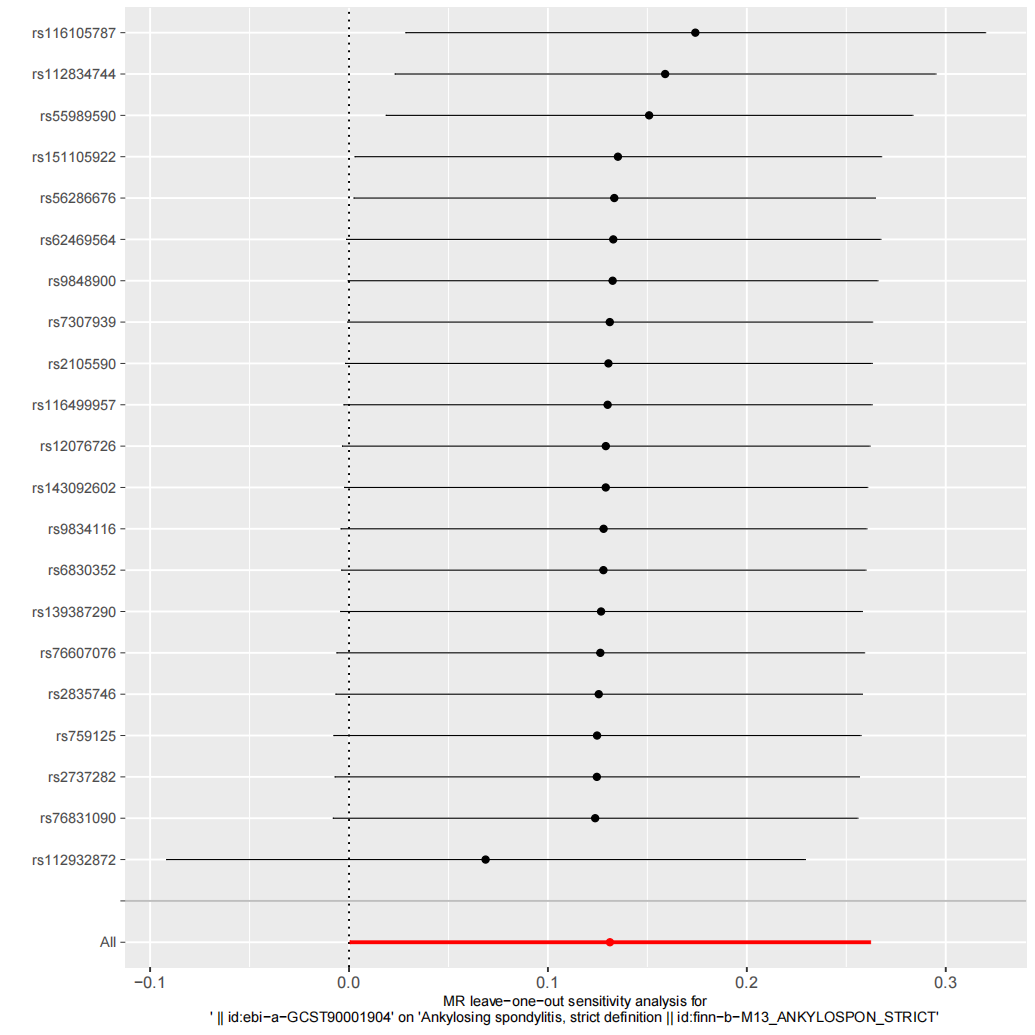
SSupplementary Figure S1M Forest plot, funnel plot, scatter plot and sensitivity analysis of SNPs associated with CD86 on CD62L+ myeloid DC on AS.


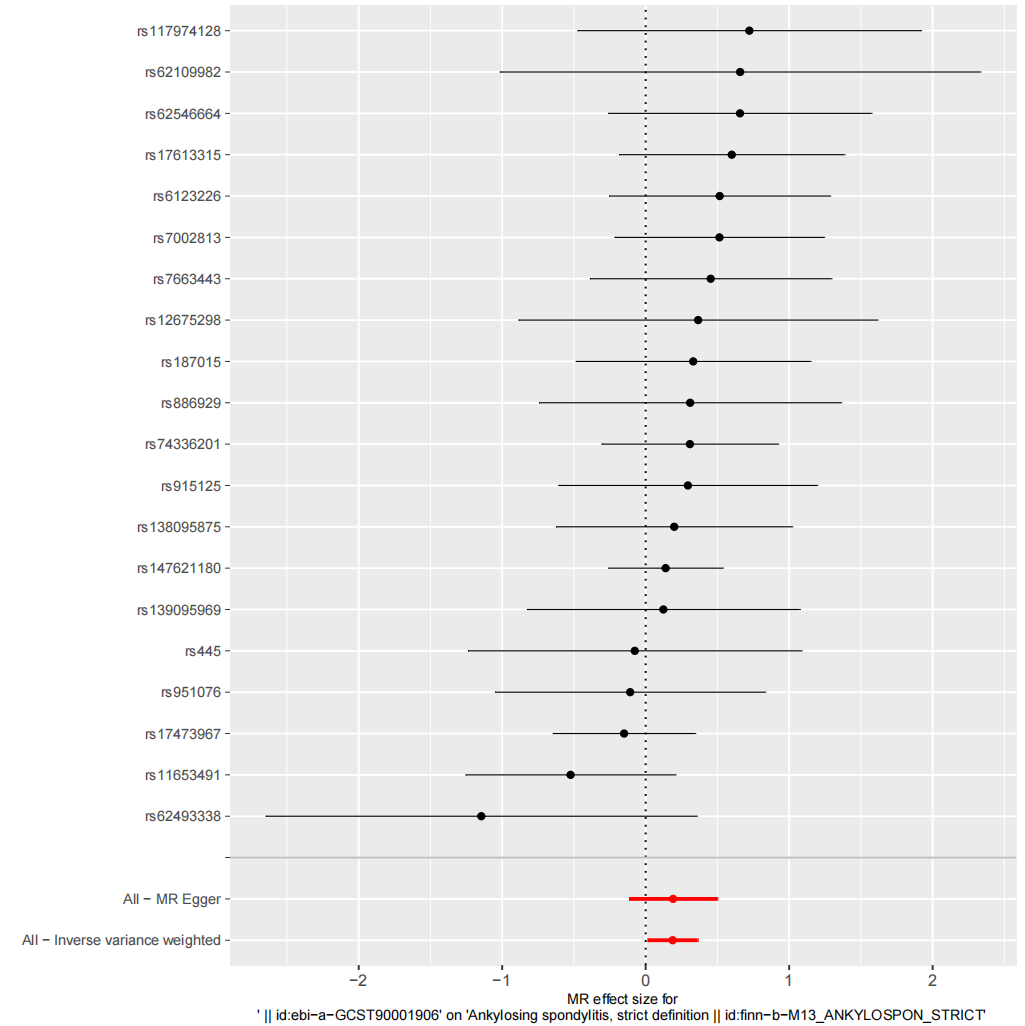

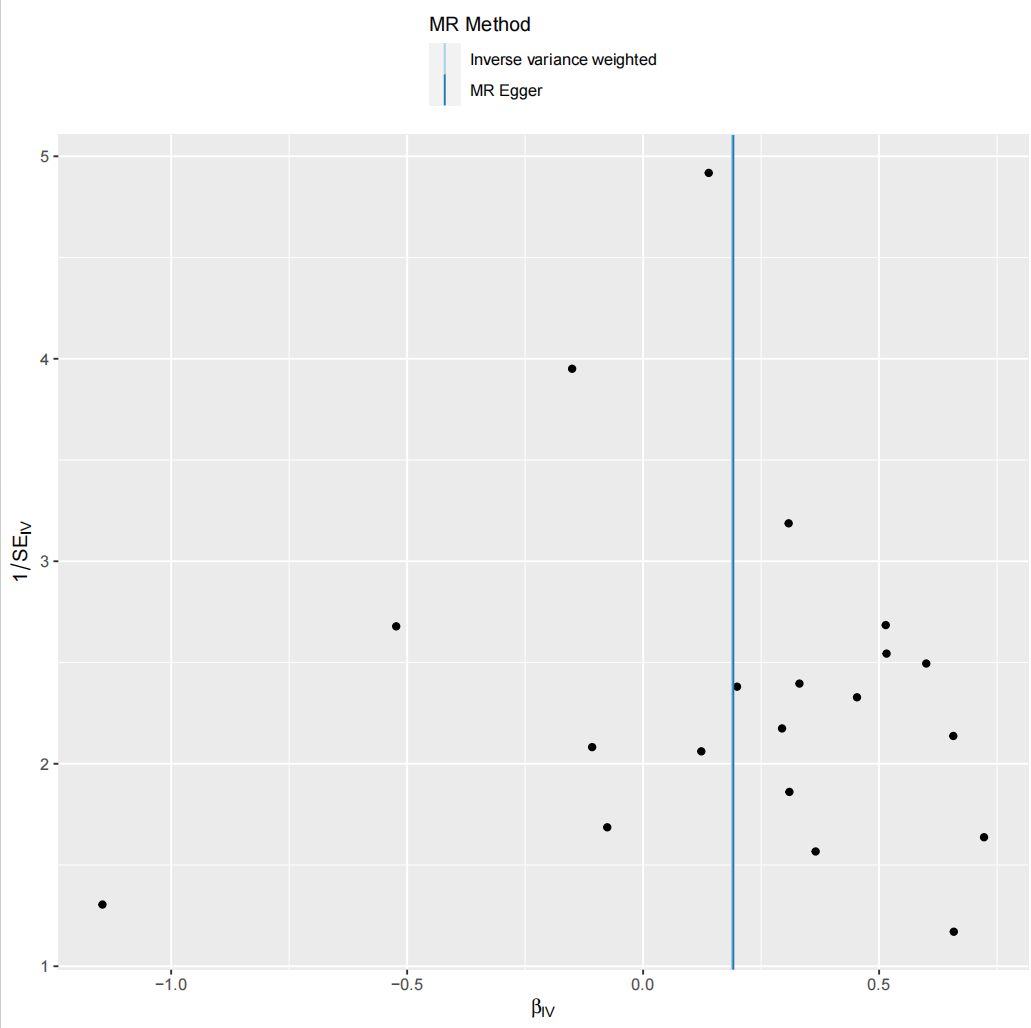

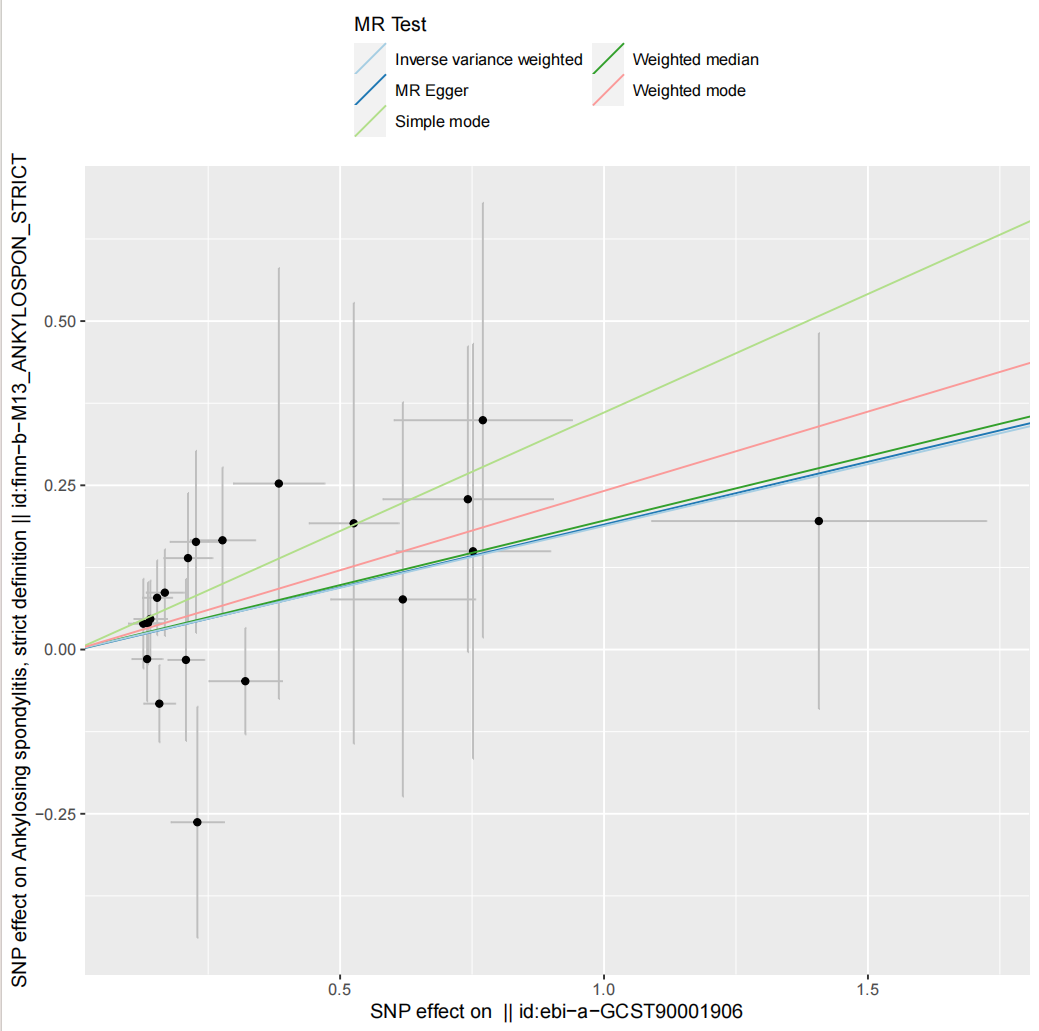

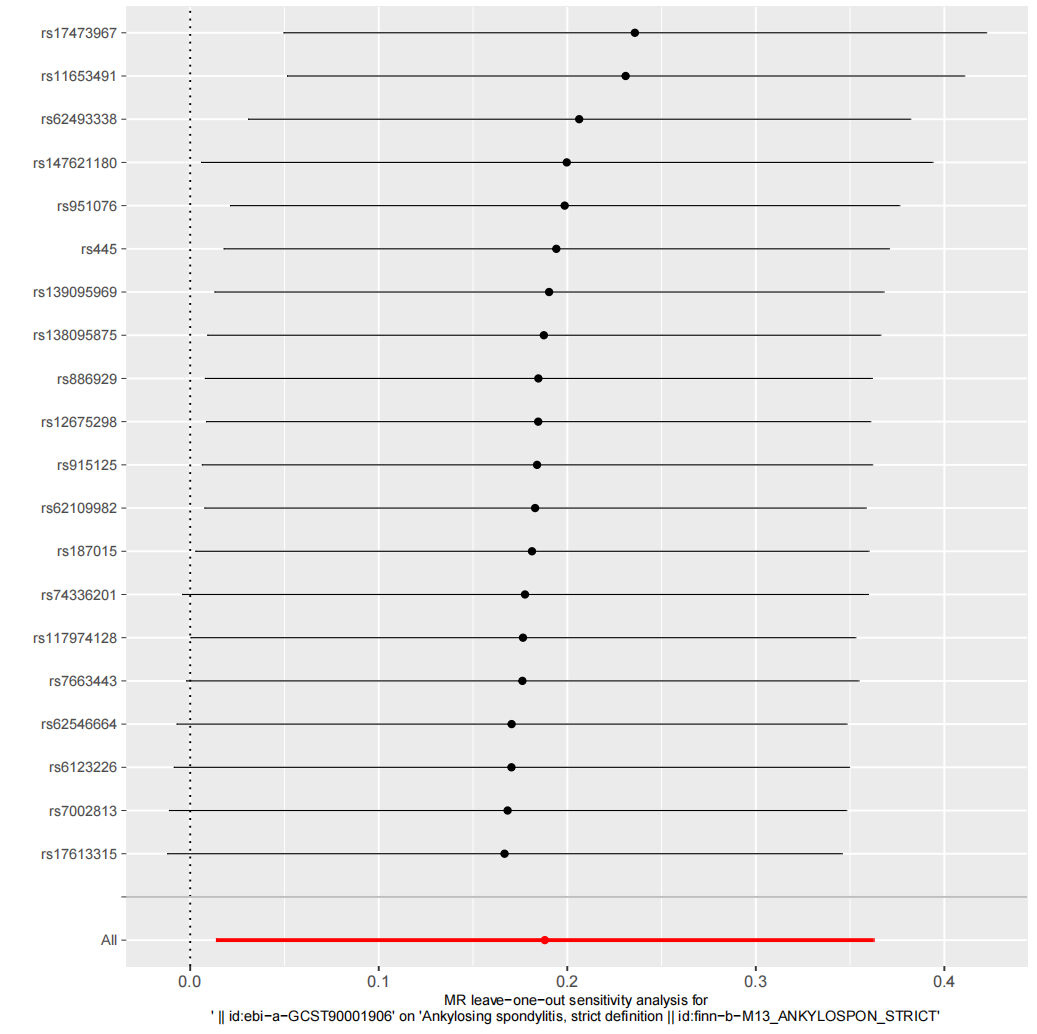


Supplementary Figure S1N Forest plot, funnel plot, scatter plot and sensitivity analysis of SNPs associated with CD86 on granulocyte on AS.


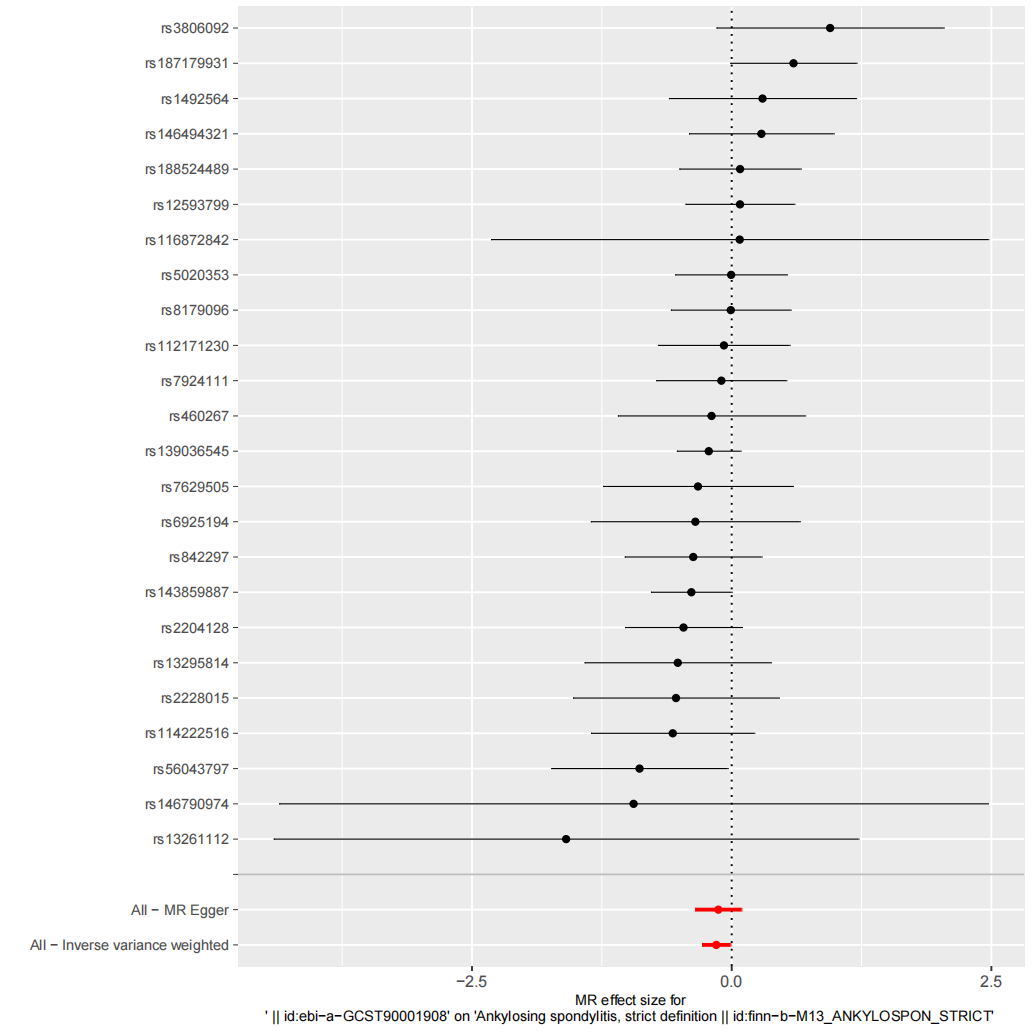

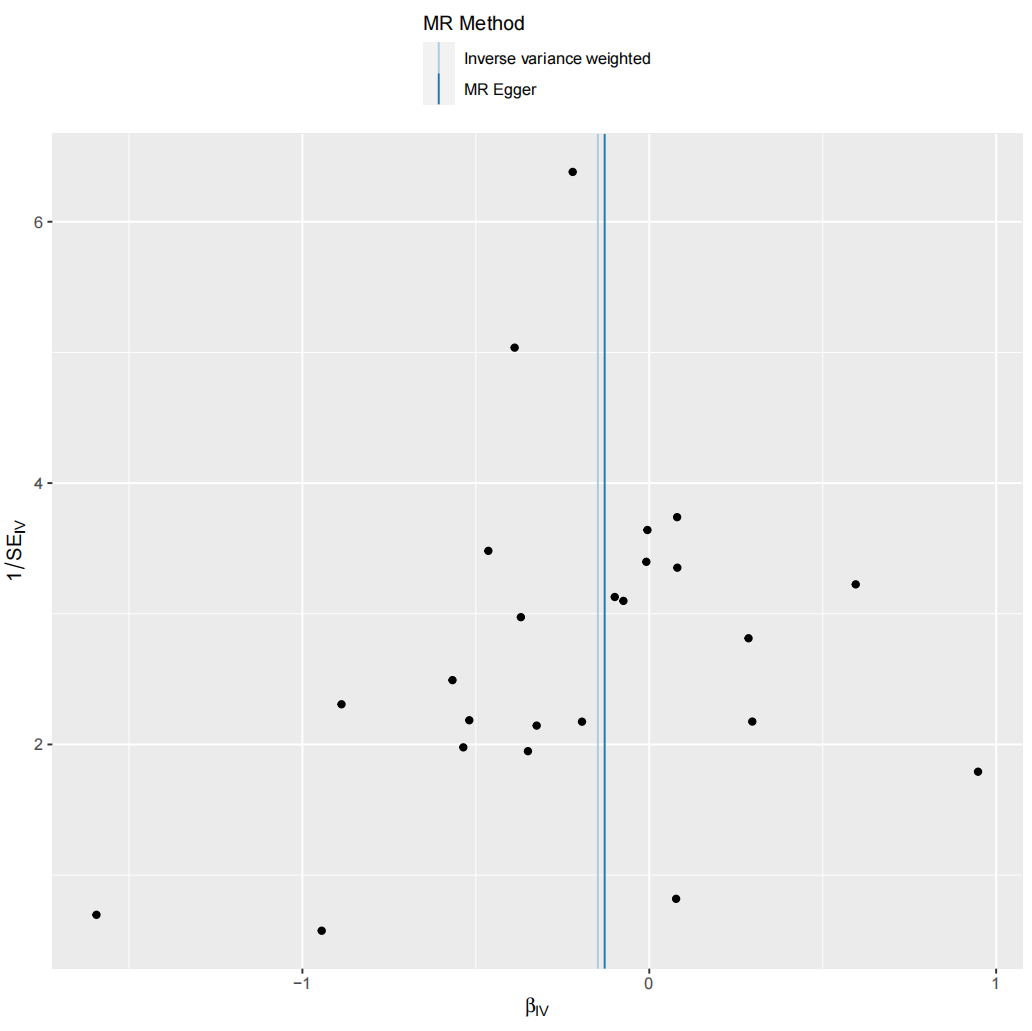

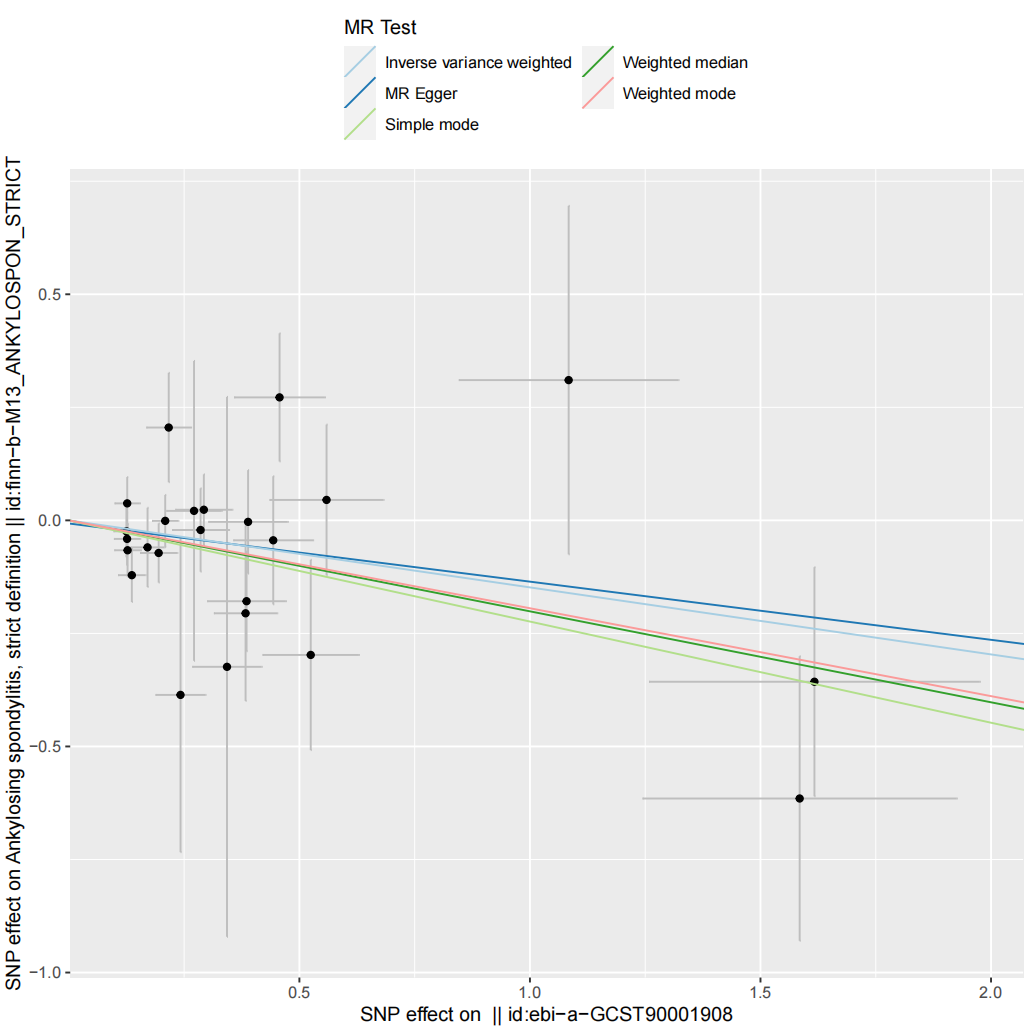

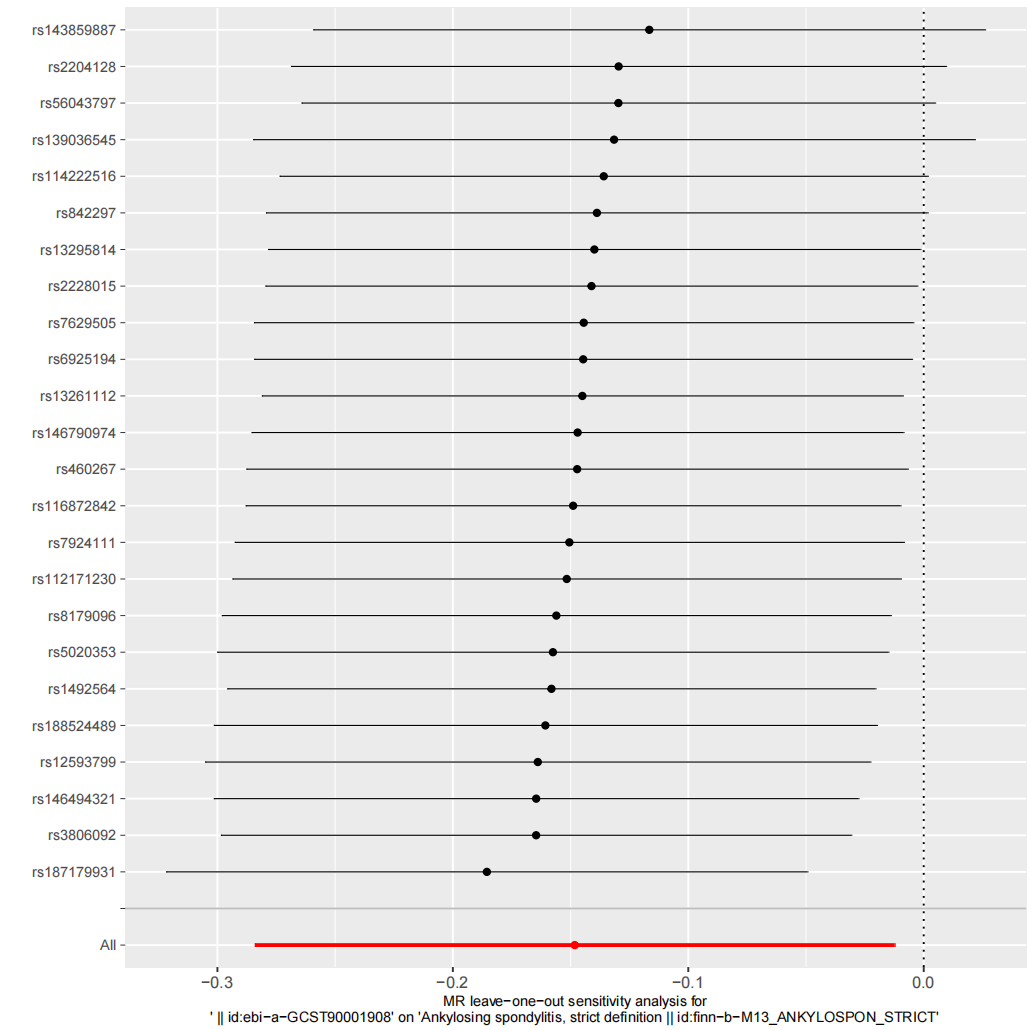


Supplementary Figure S1O Forest plot, funnel plot, scatter plot and sensitivity analysis of SNPs associated with CCR7 on naive CD8br on AS.


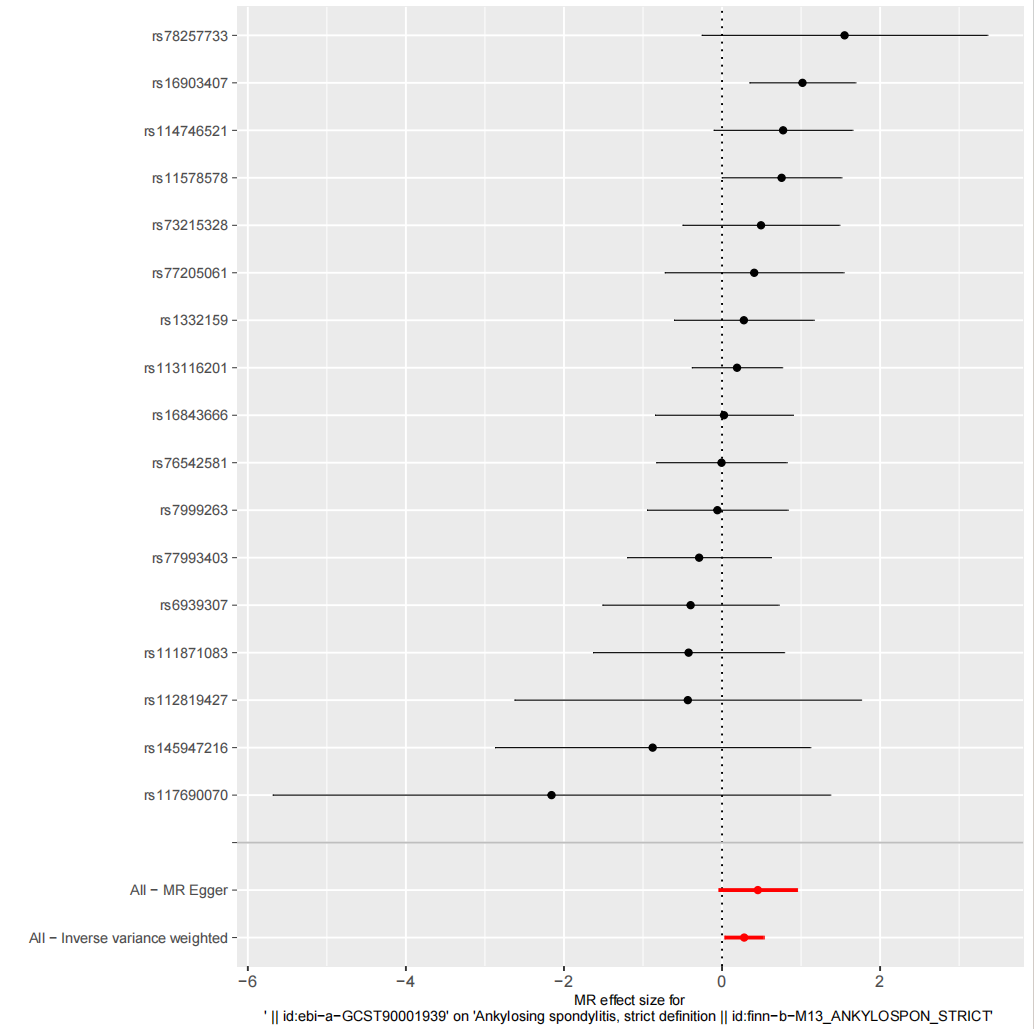

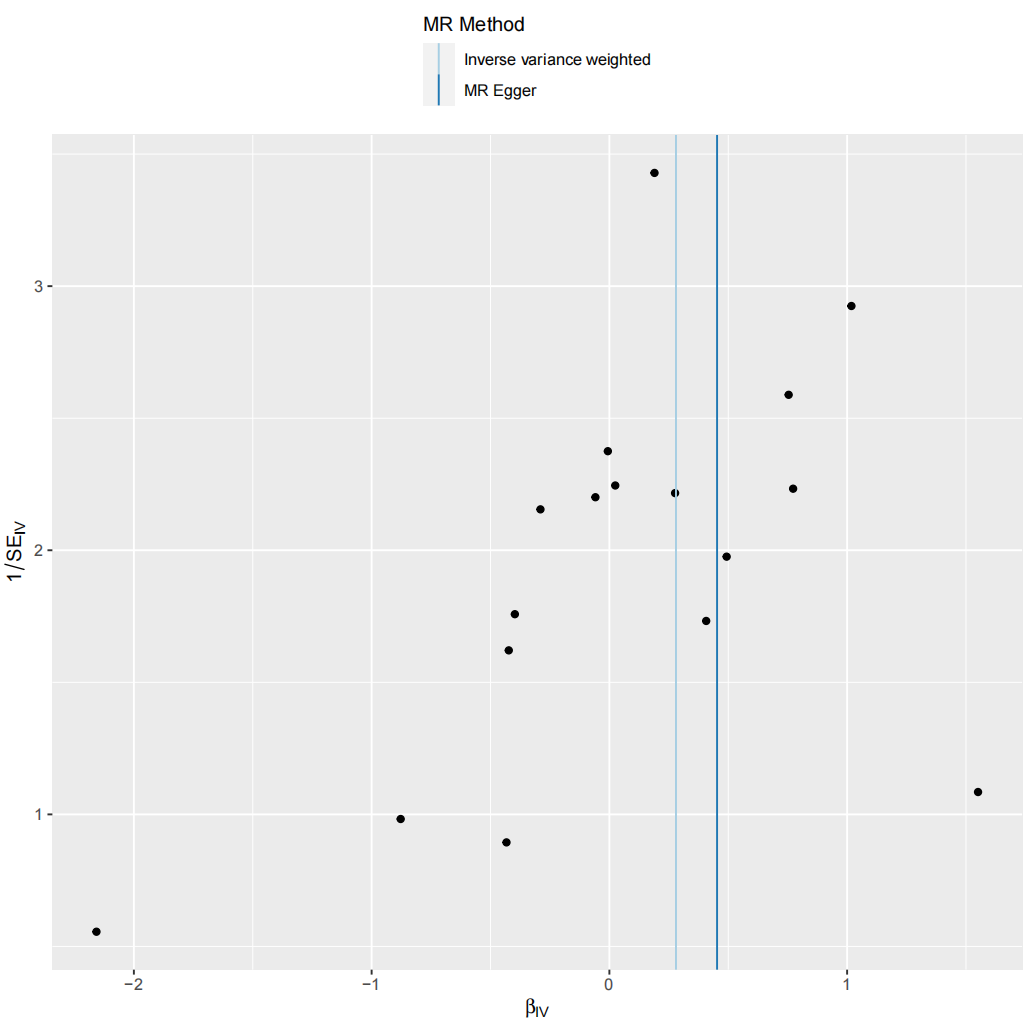

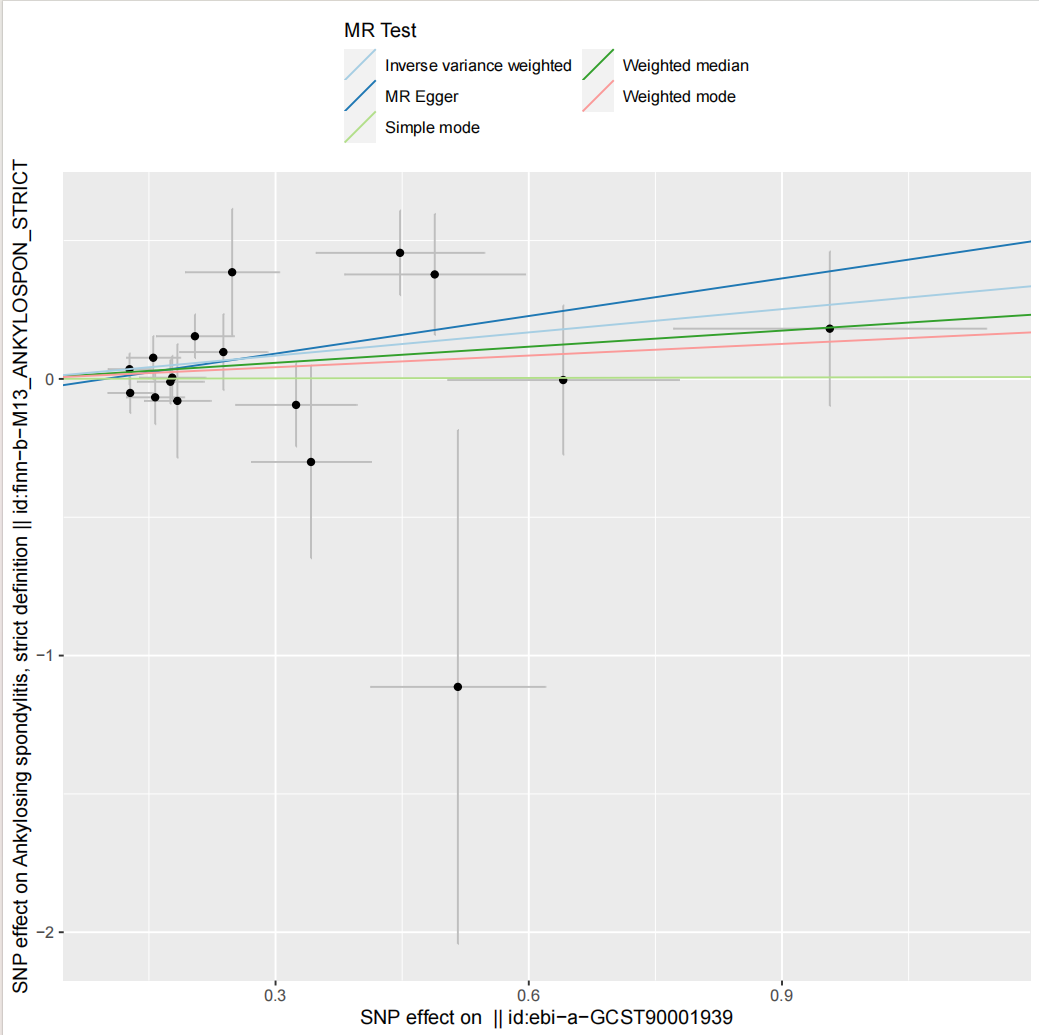

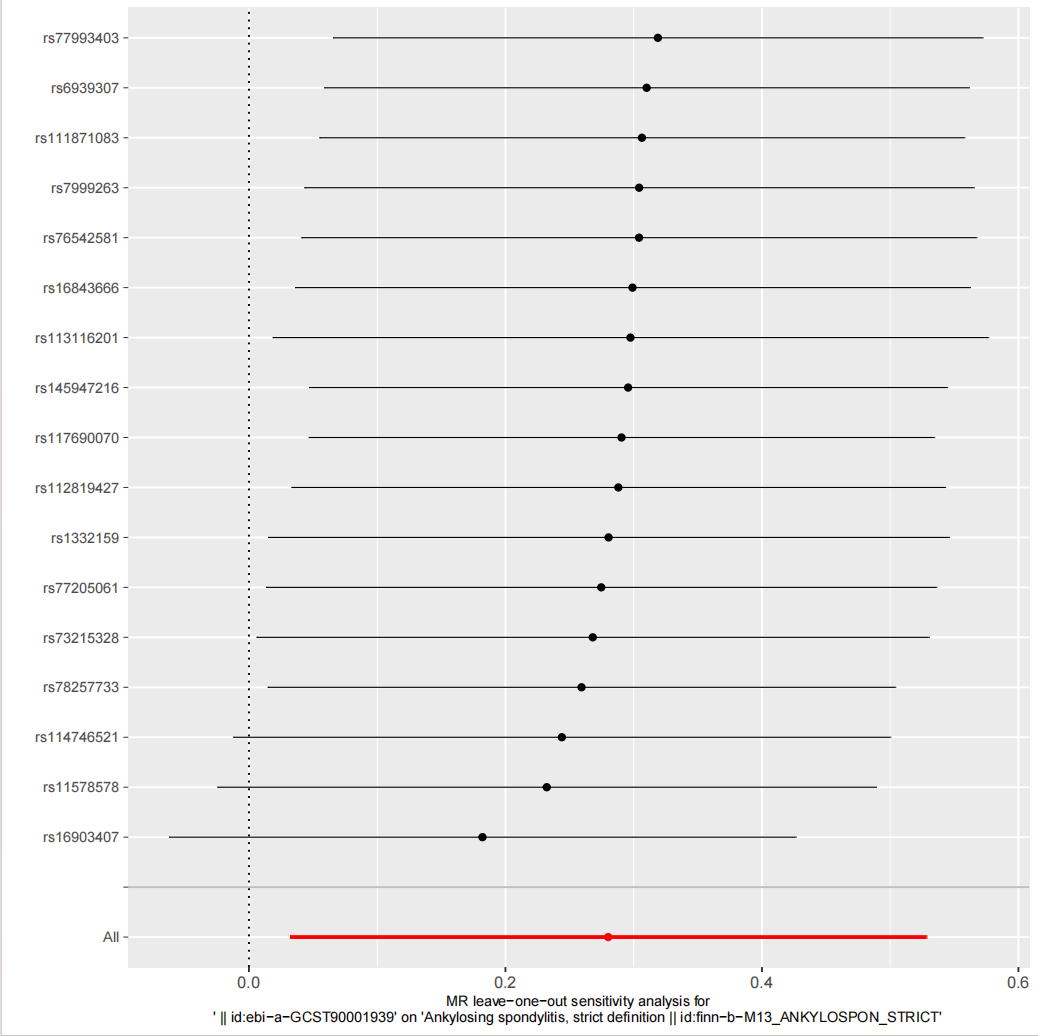


Supplementary Figure S1P Forest plot, funnel plot, scatter plot and sensitivity analysis of SNPs associated with CD25 on activated Treg on AS.


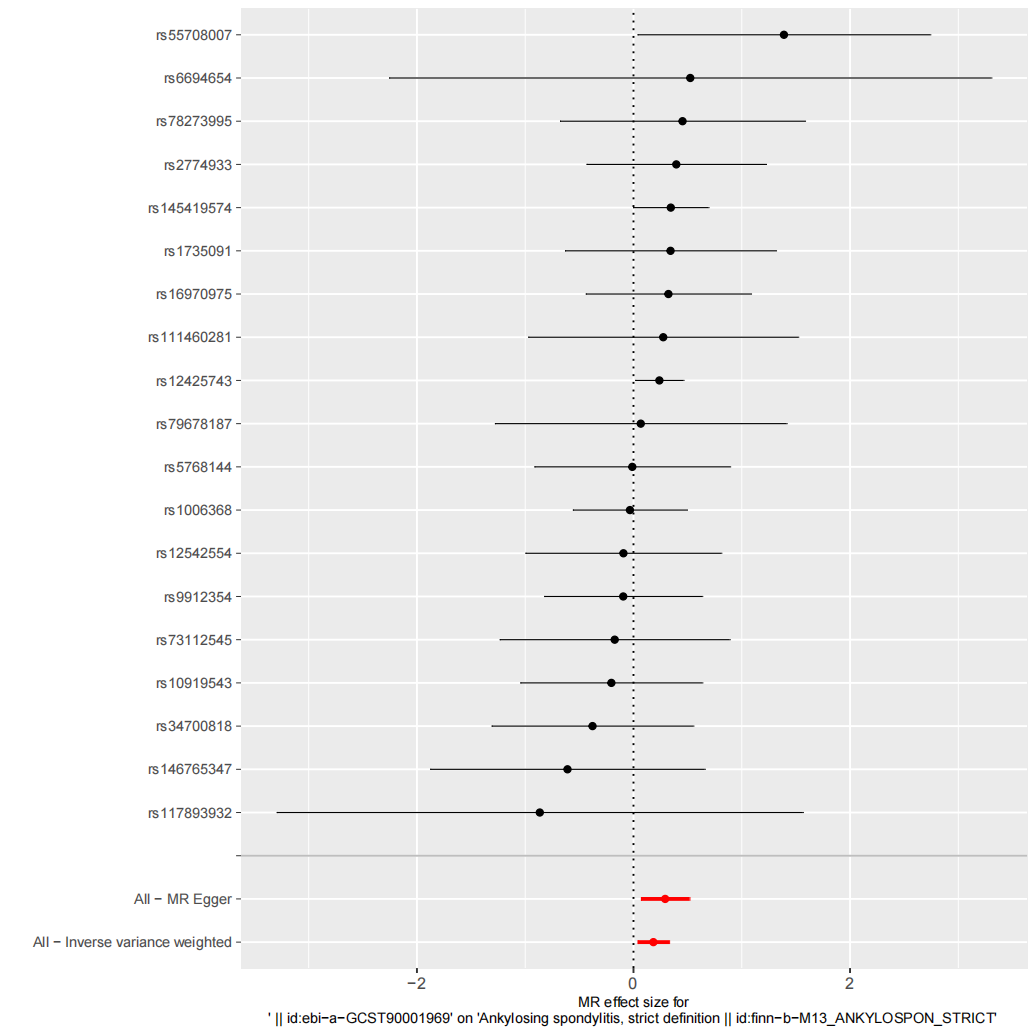

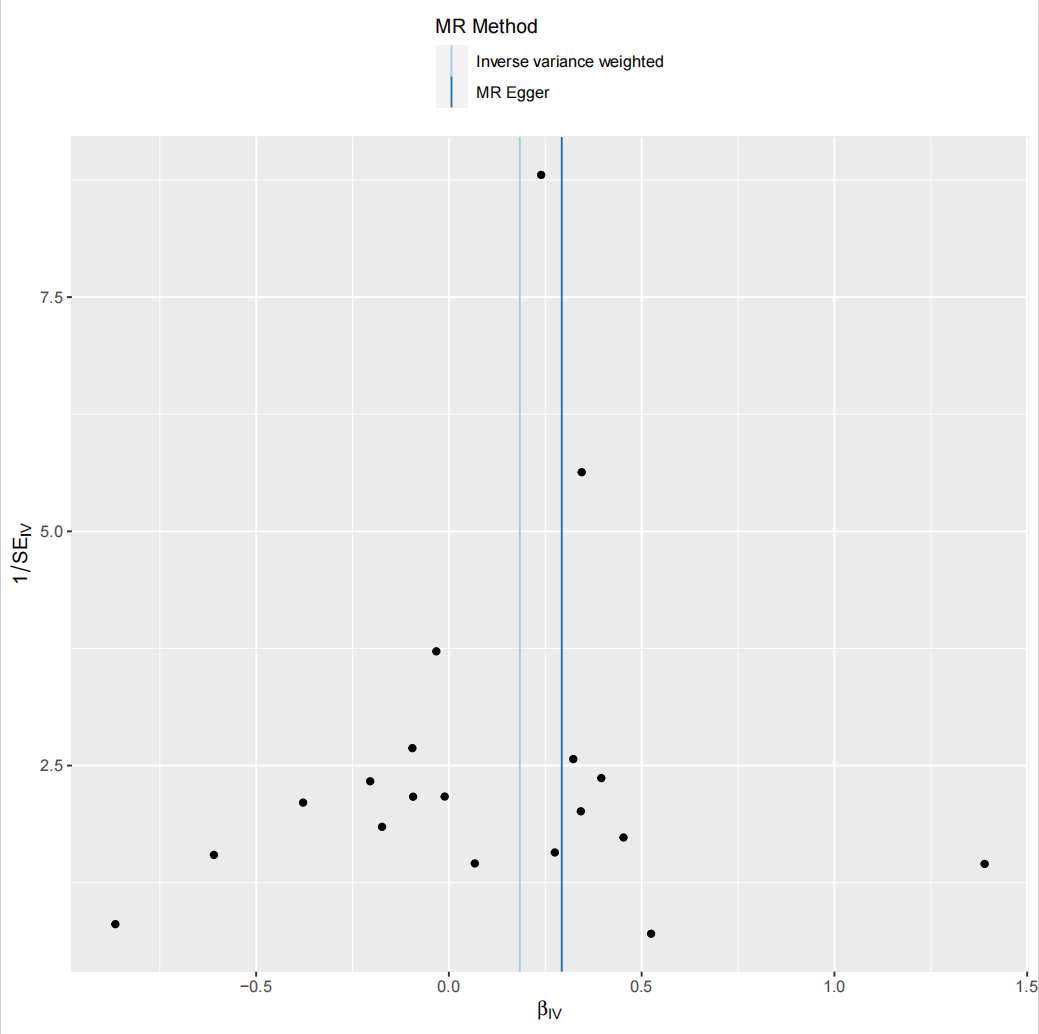

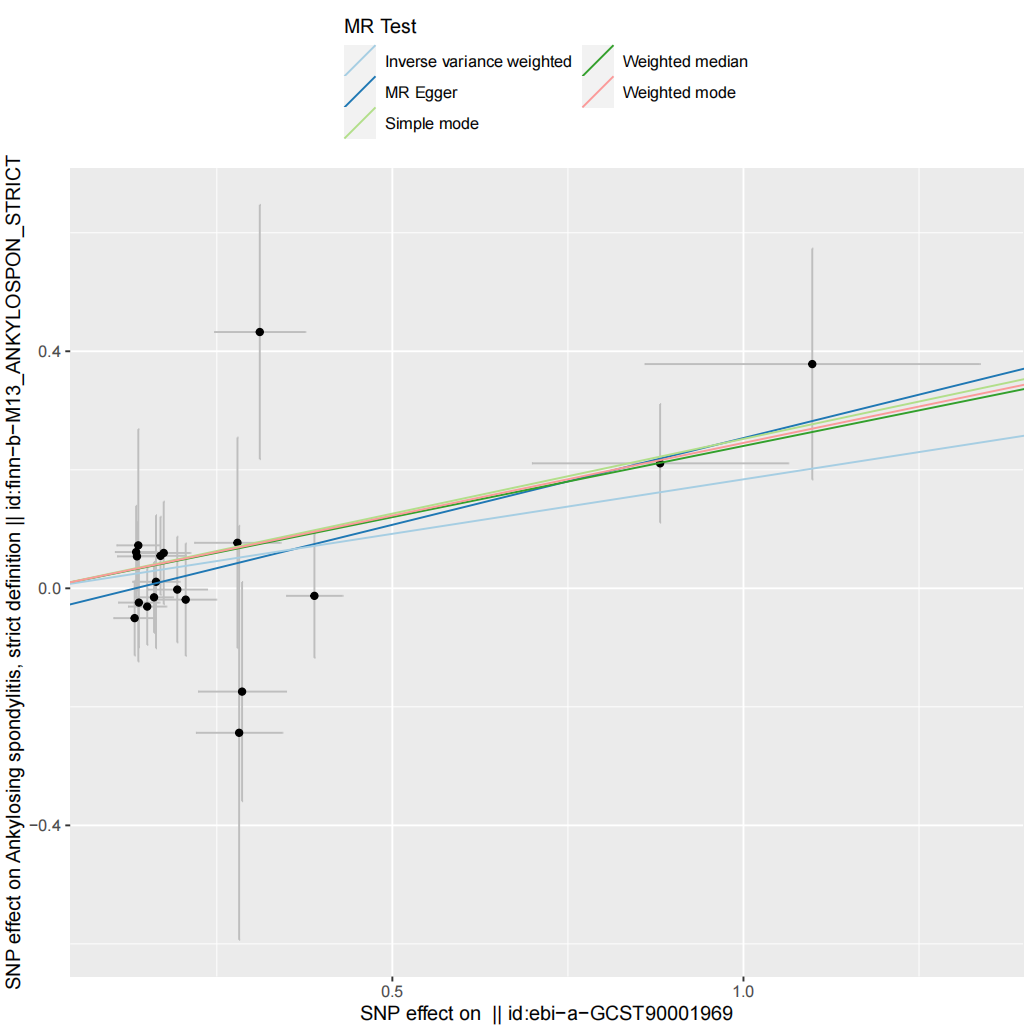

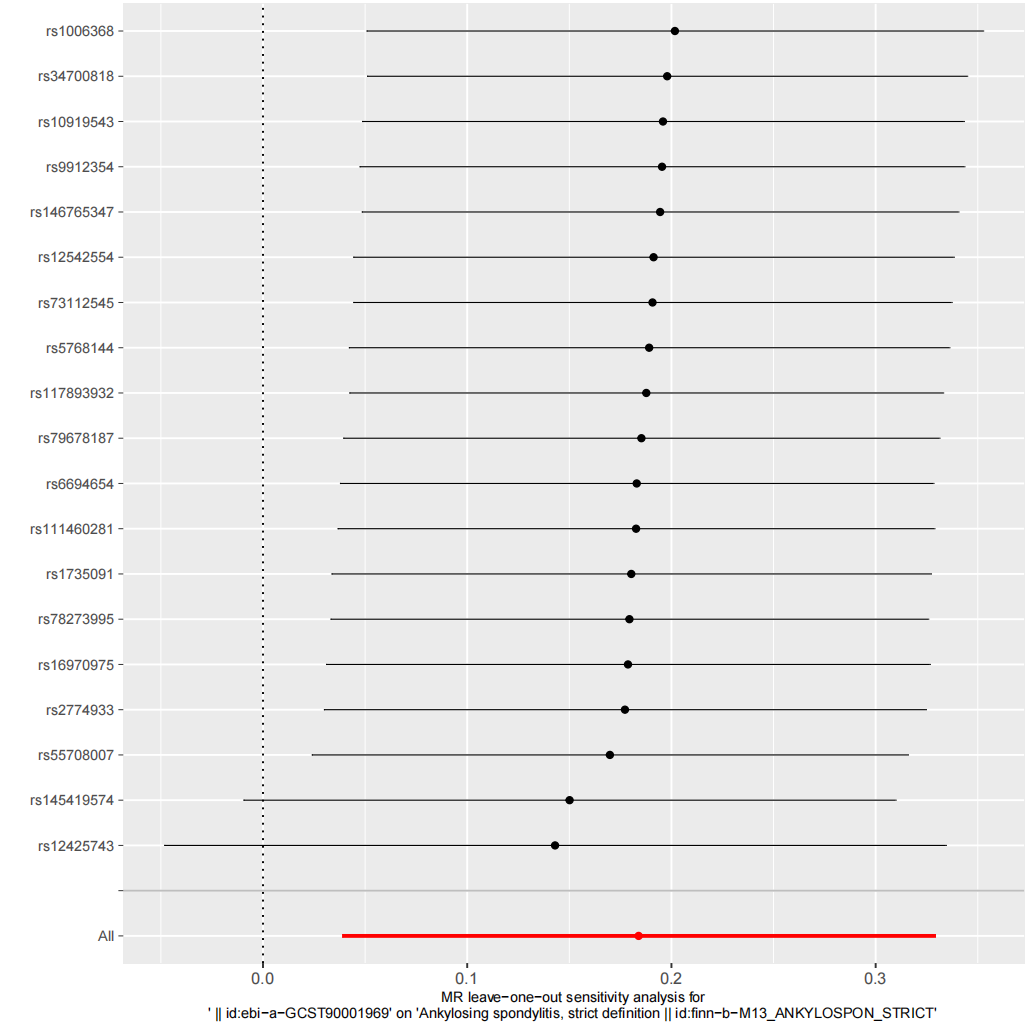


Supplementary Figure S1Q Forest plot, funnel plot, scatter plot and sensitivity analysis of SNPs associated with FSC-A on NK on AS.


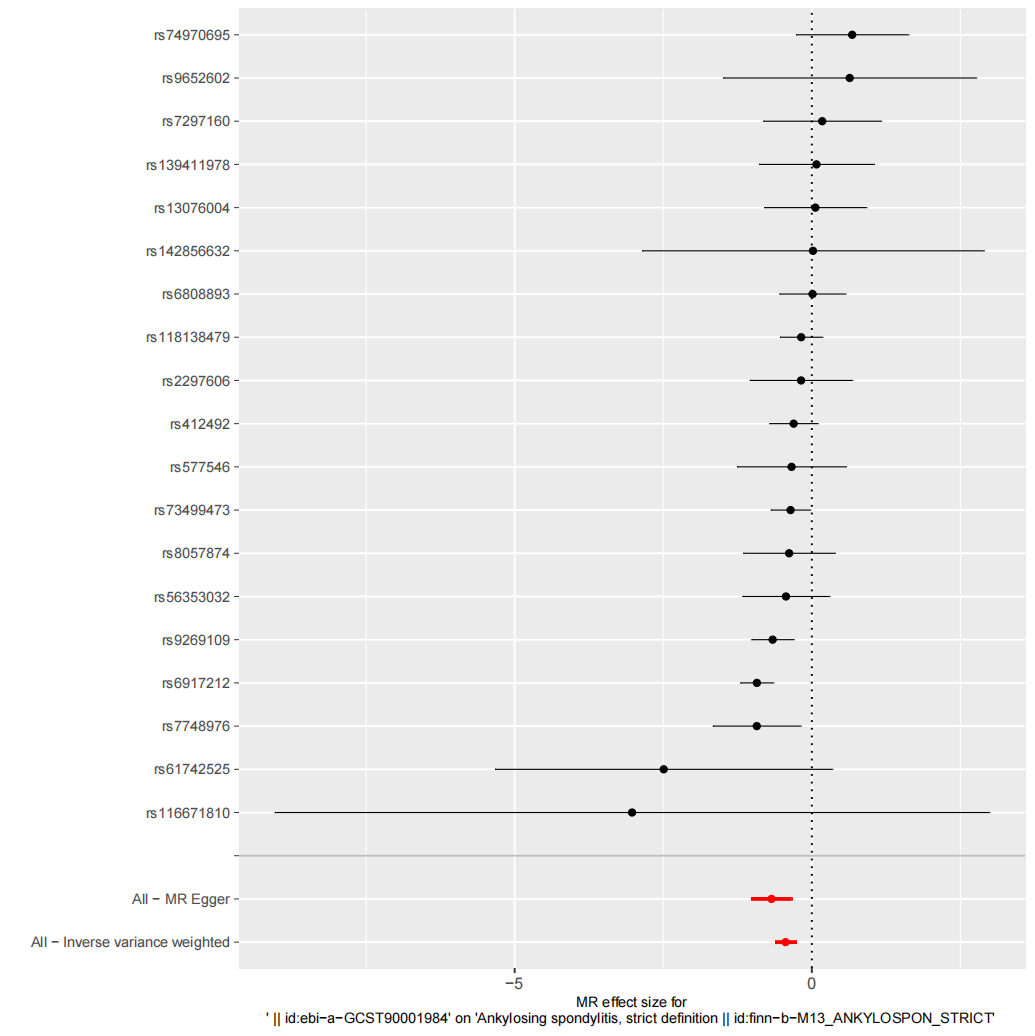

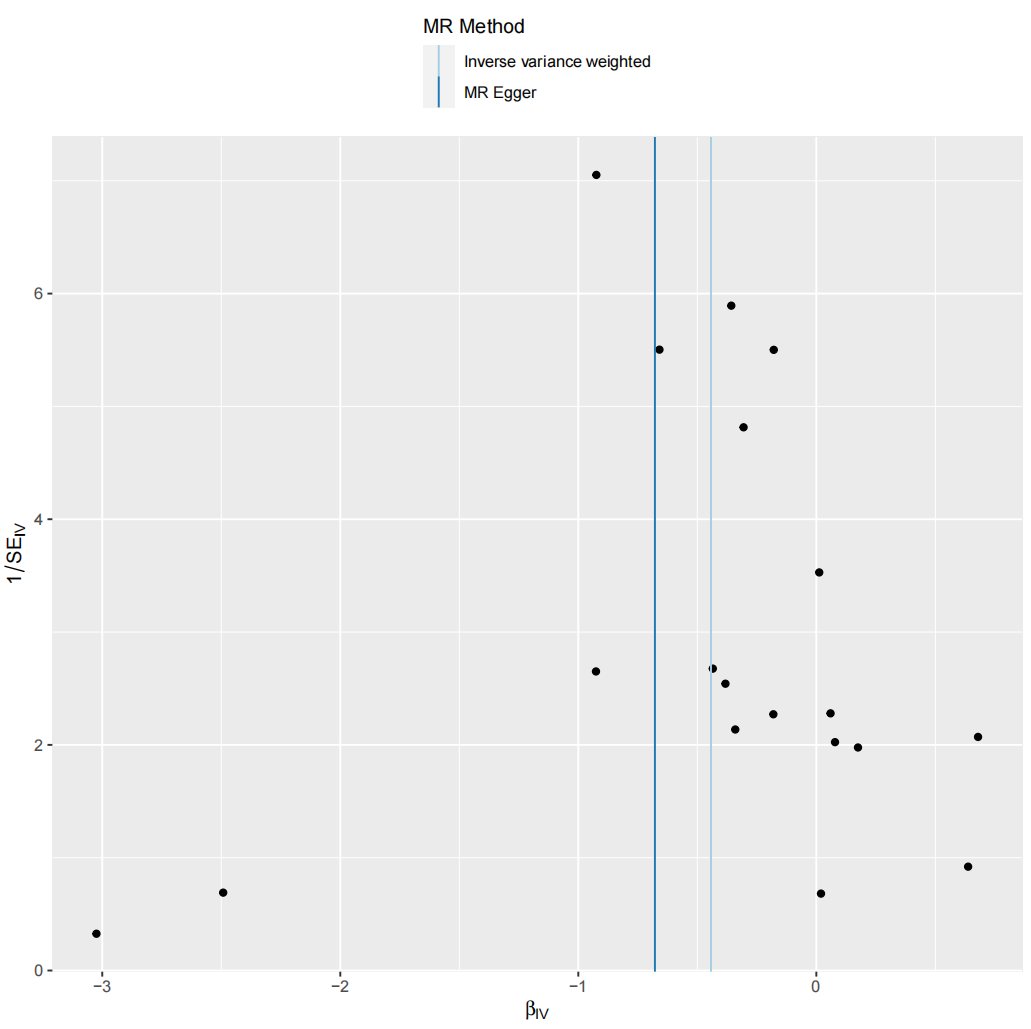

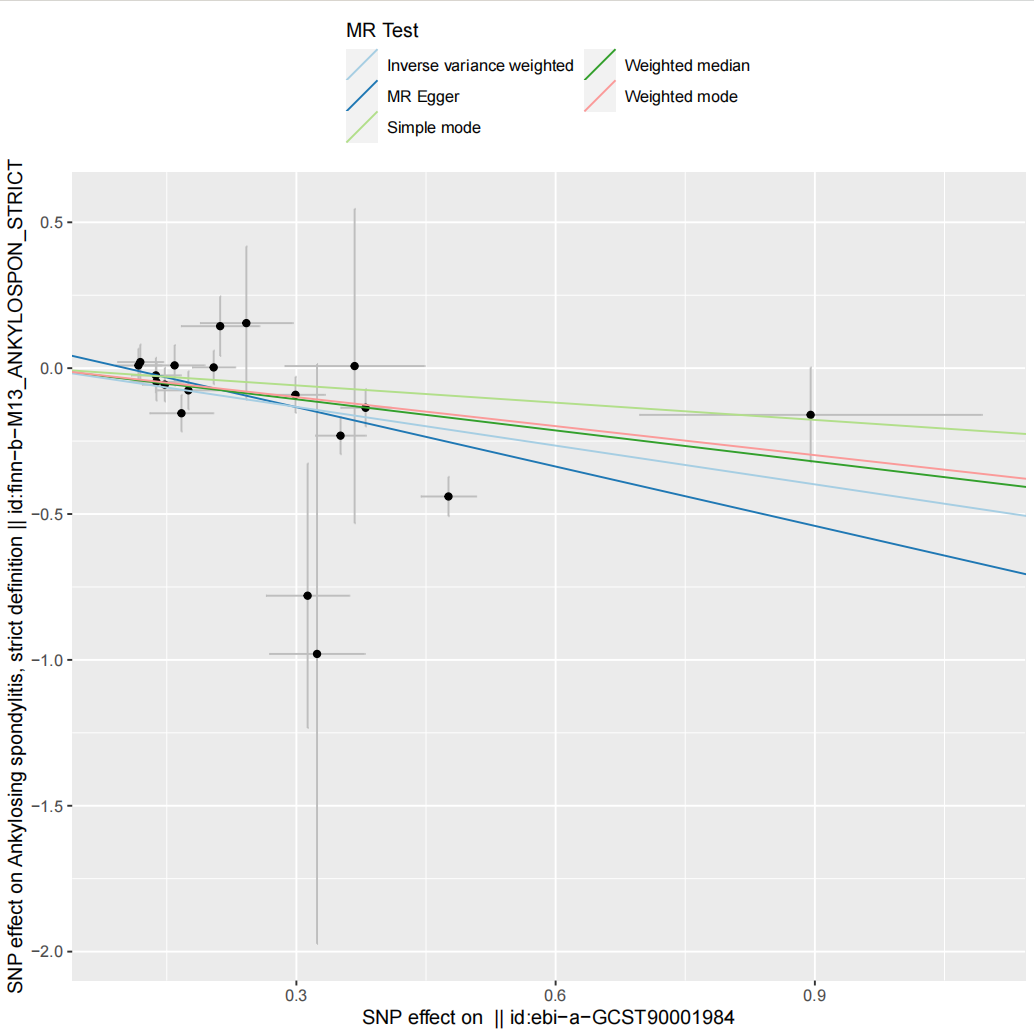

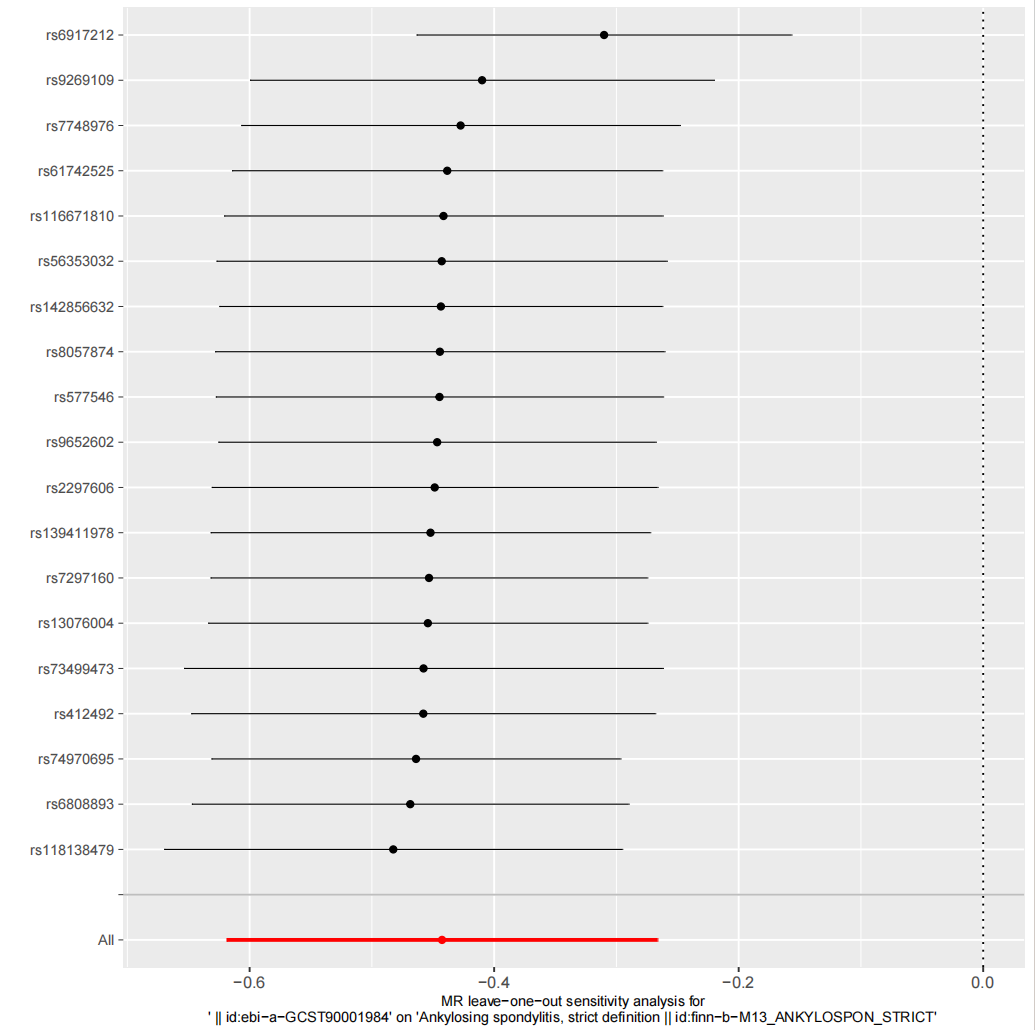
Supplementary Figure S1R Forest plot, funnel plot, scatter plot and sensitivity analysis of SNPs associated with HLA DR on CD14- CD16+ monocyte on AS.


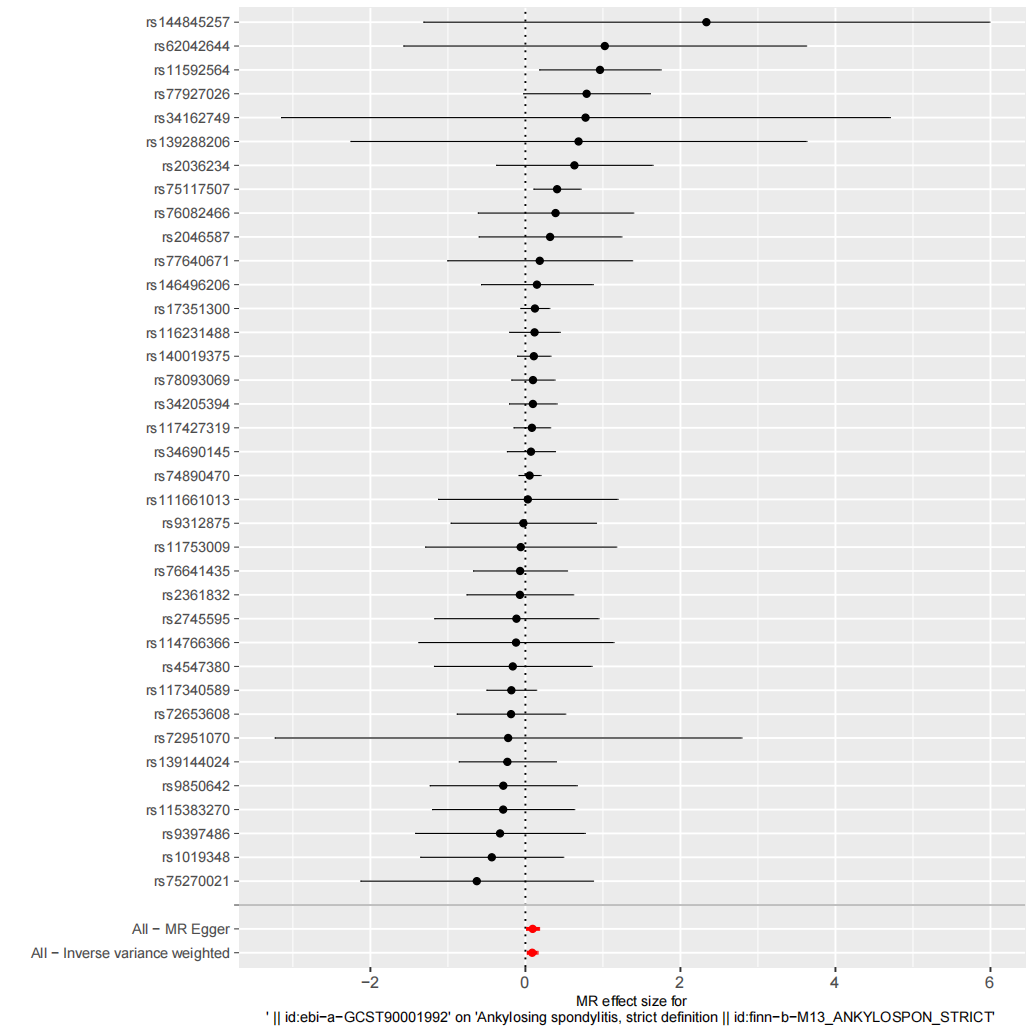

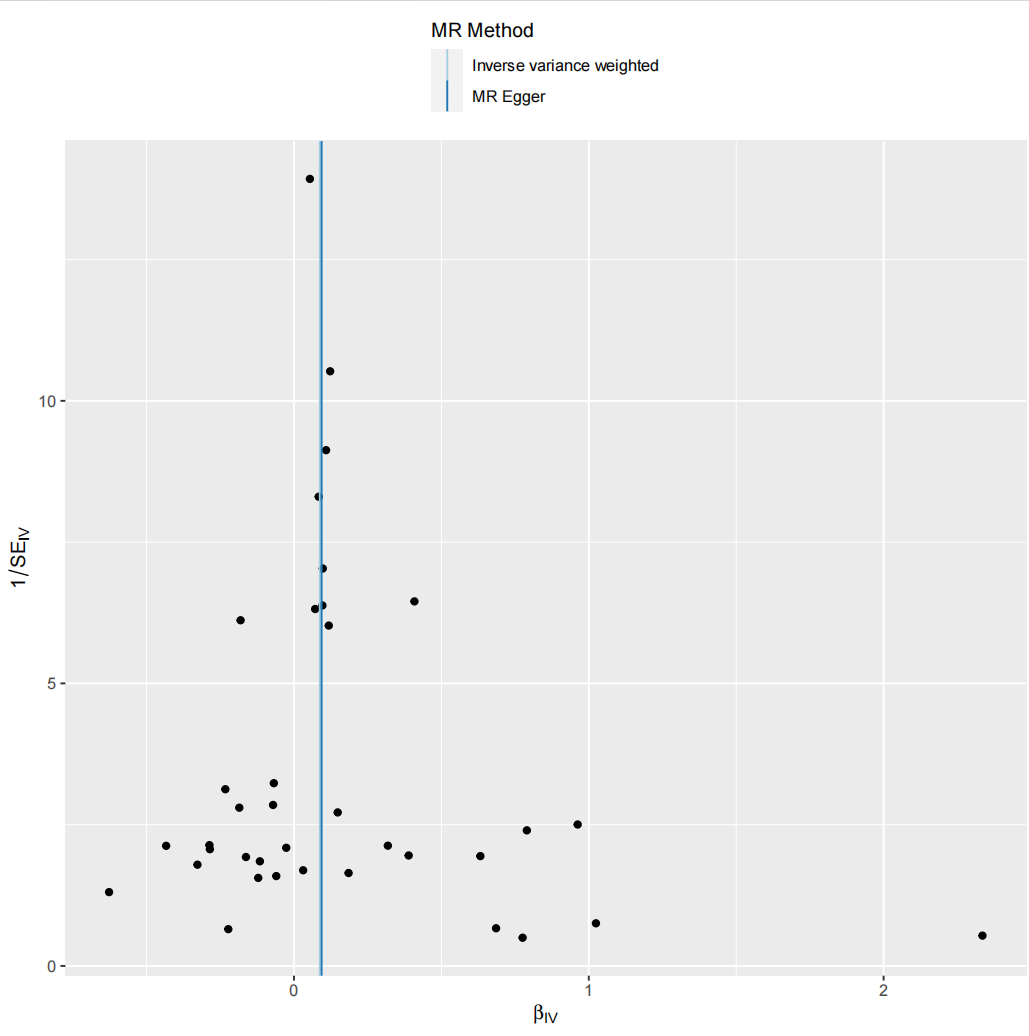

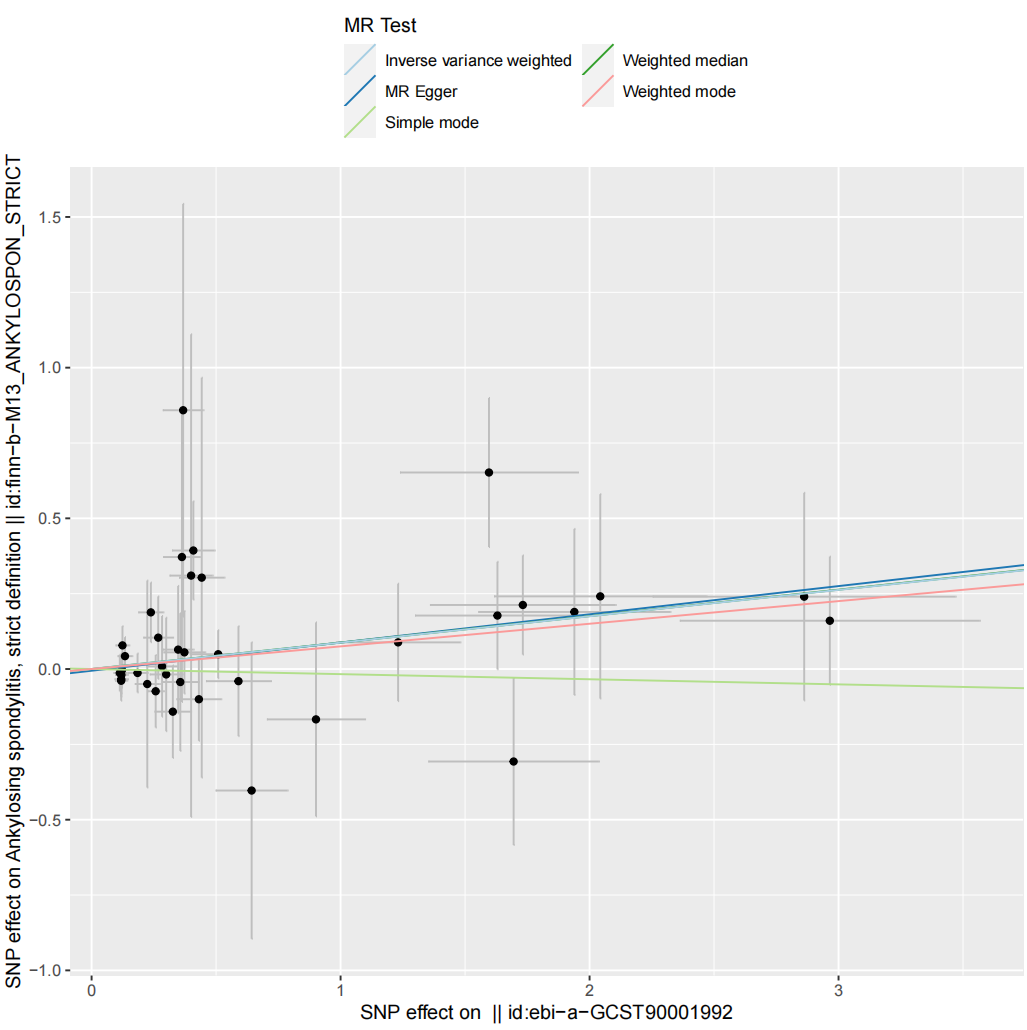

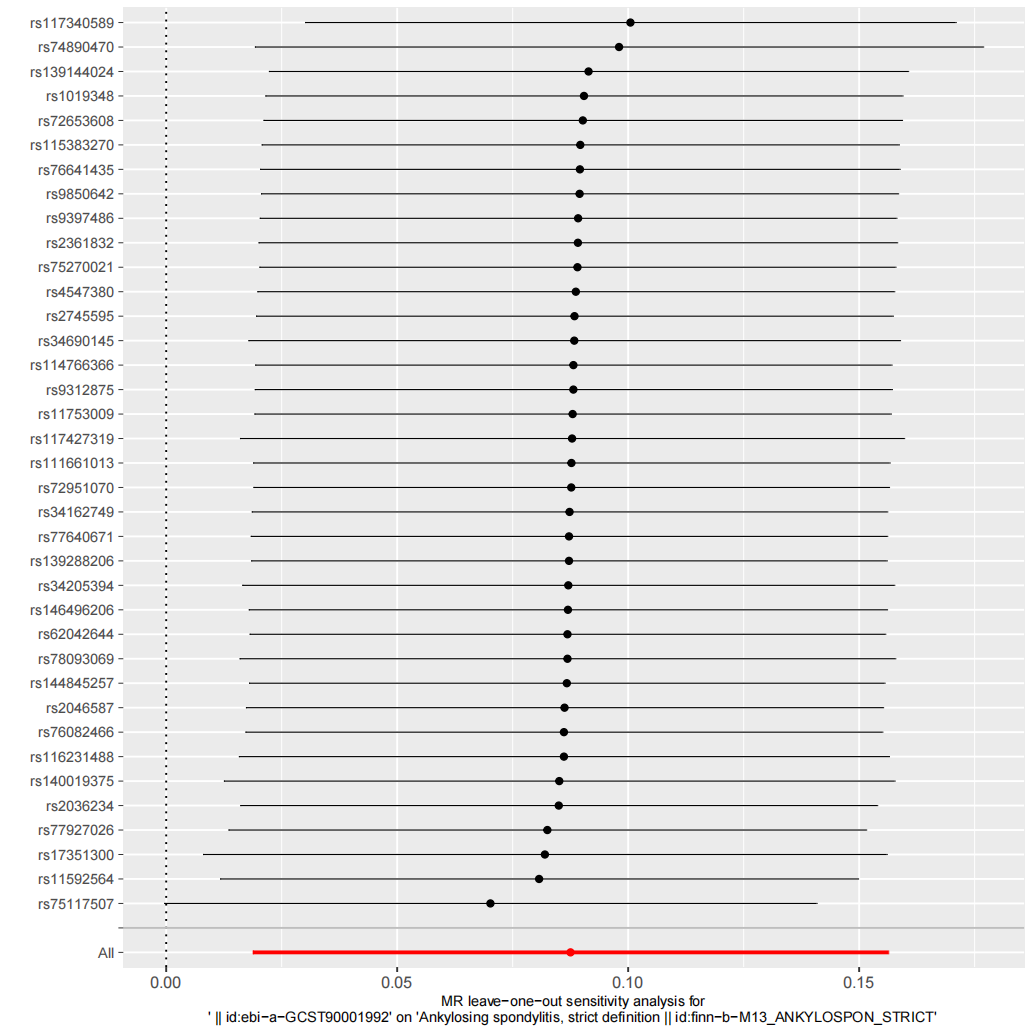


Supplementary Figure S1S Forest plot, funnel plot, scatter plot and sensitivity analysis of SNPs associated with CCR2 on CD14+ CD16+ monocyte on AS.


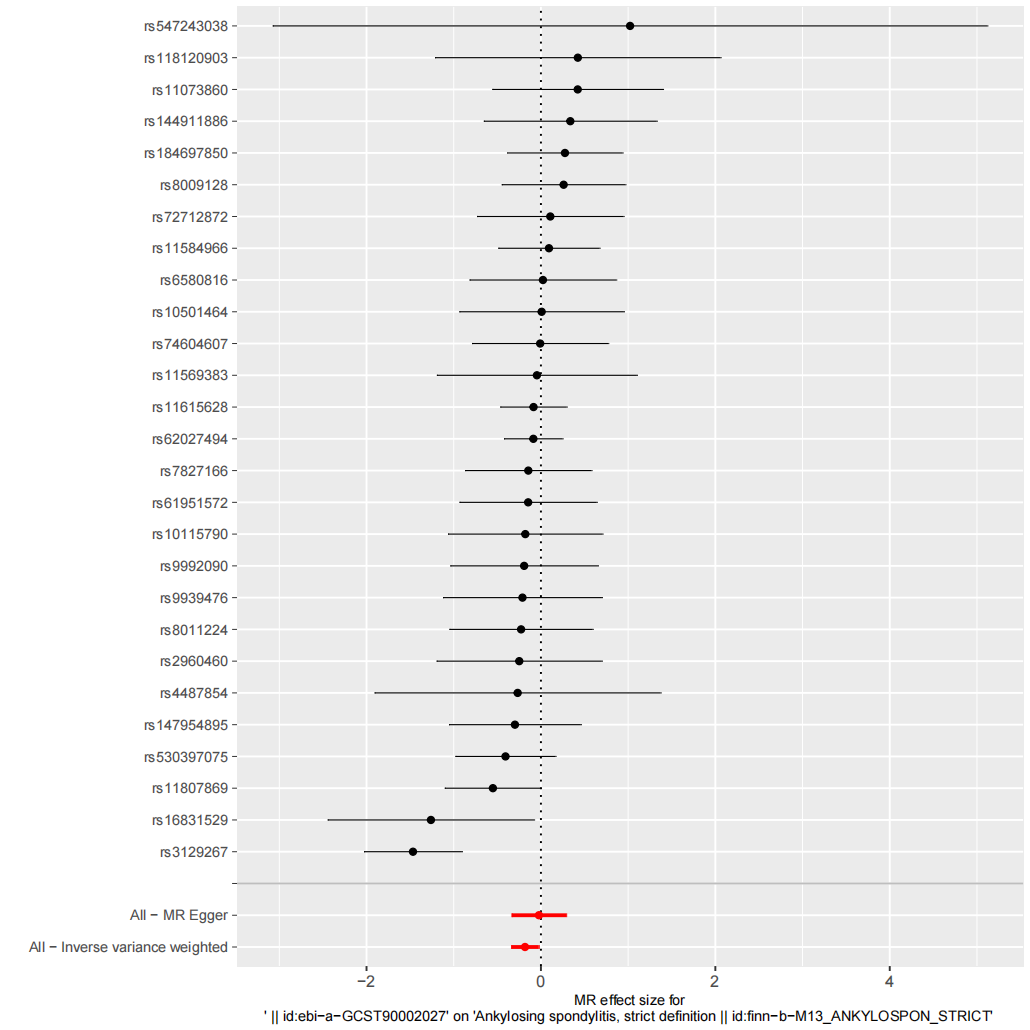

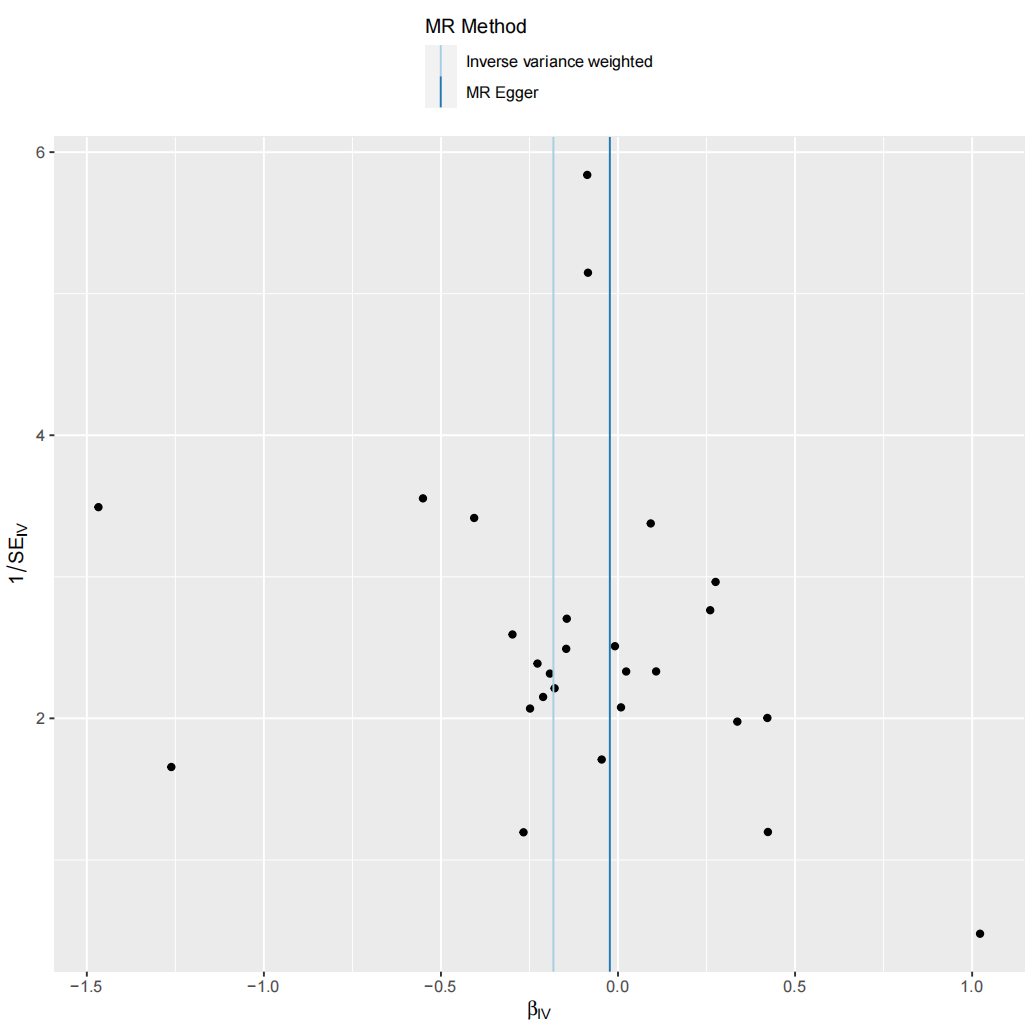

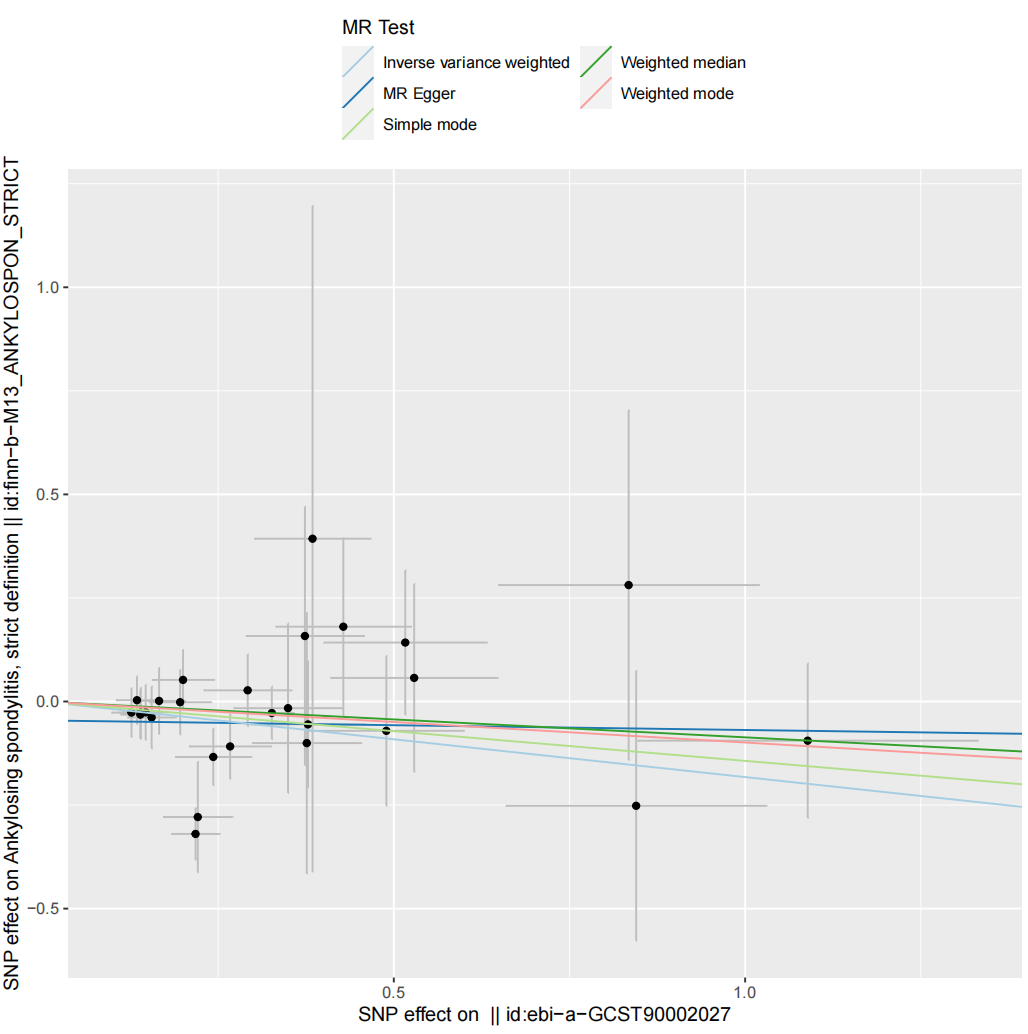

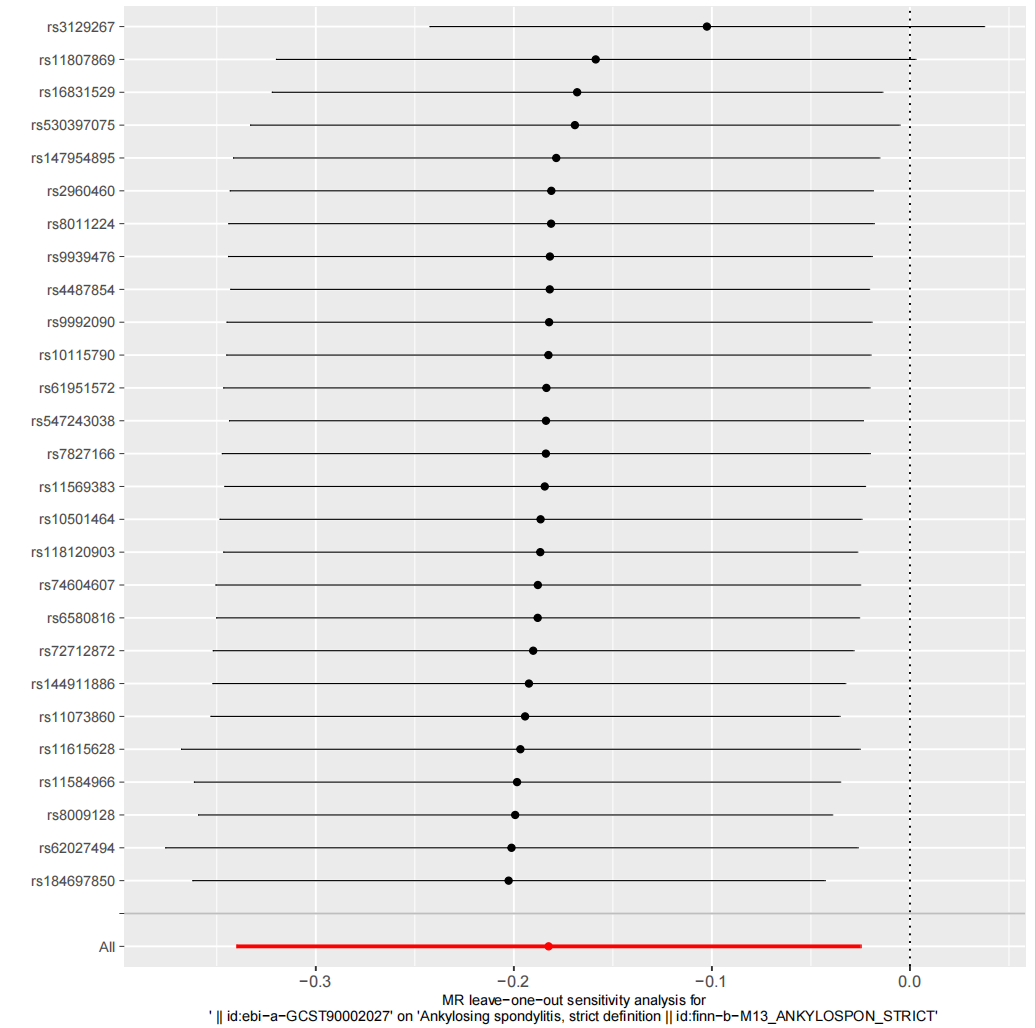


Supplementary Figure S1T Forest plot, funnel plot, scatter plot and sensitivity analysis of SNPs associated with CD4 on CD45RA+ CD4+ on AS.


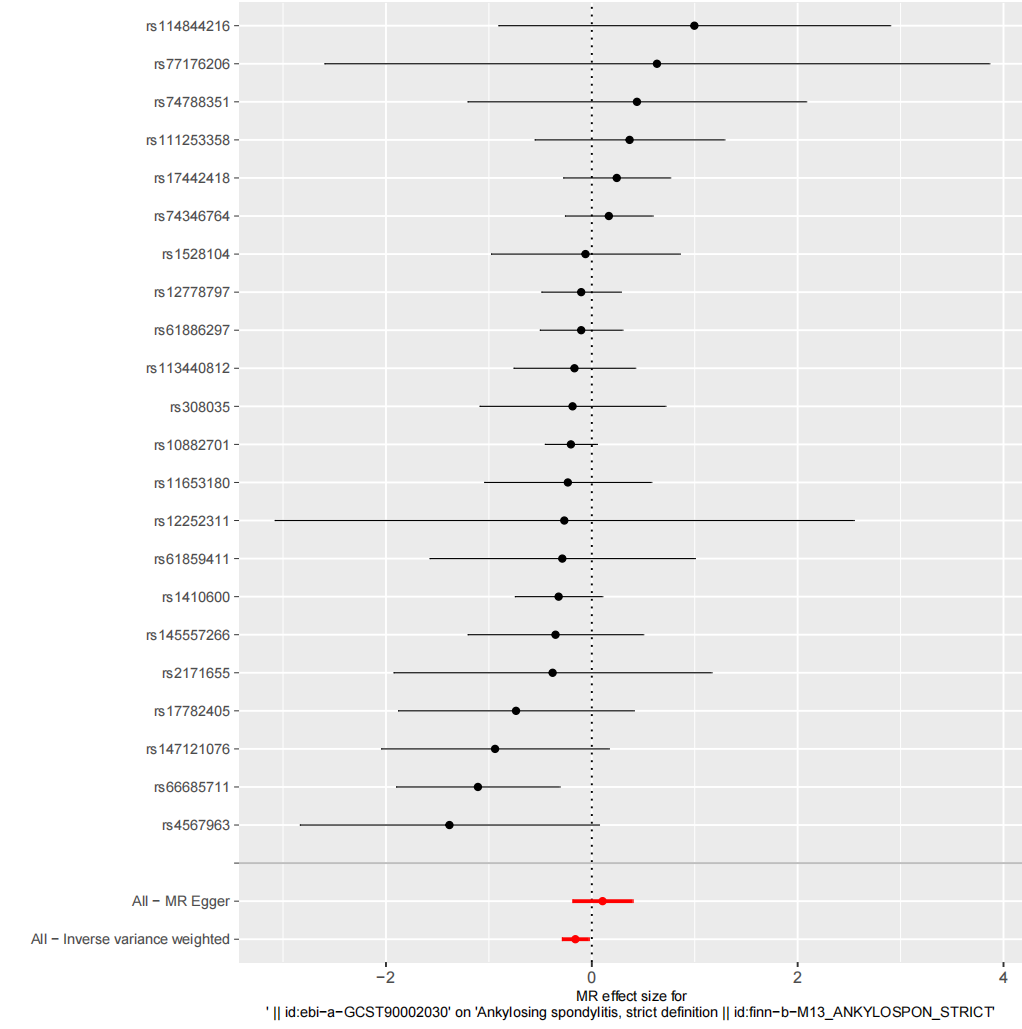

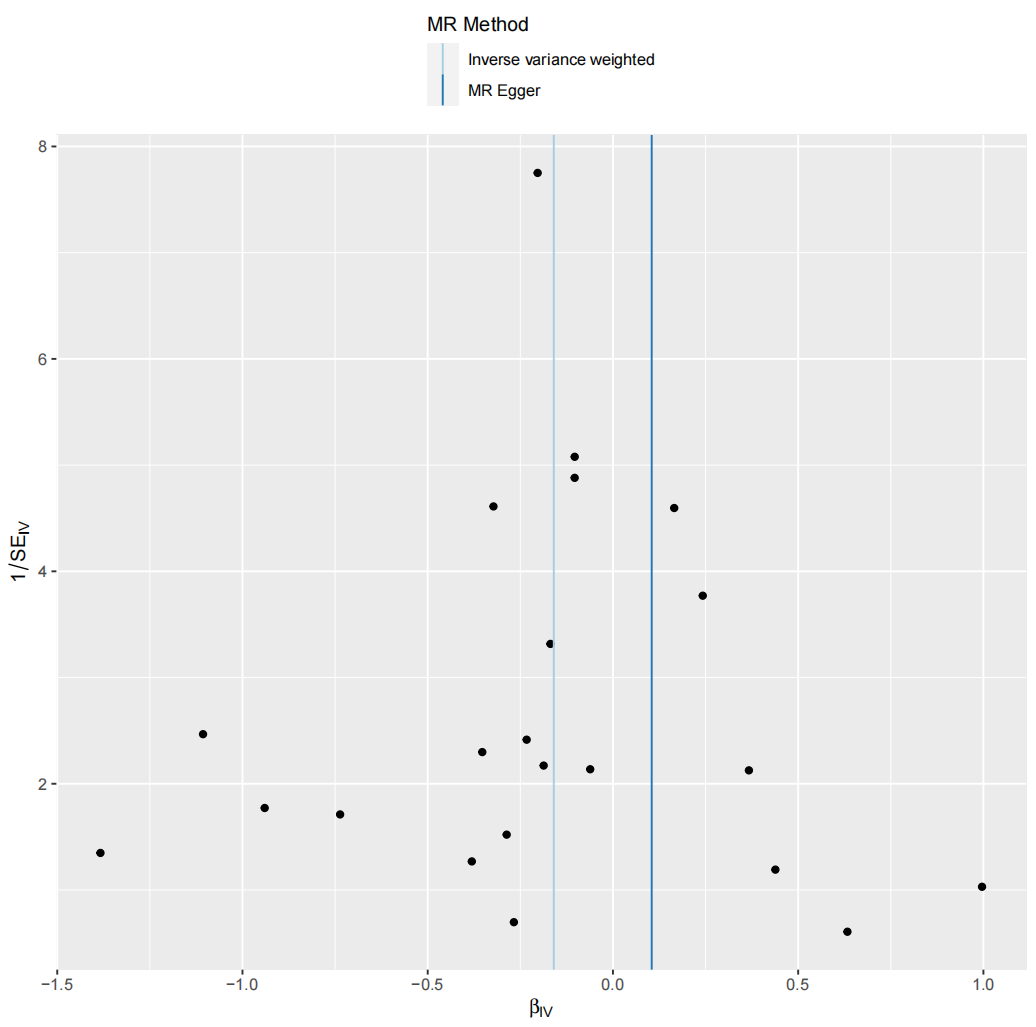

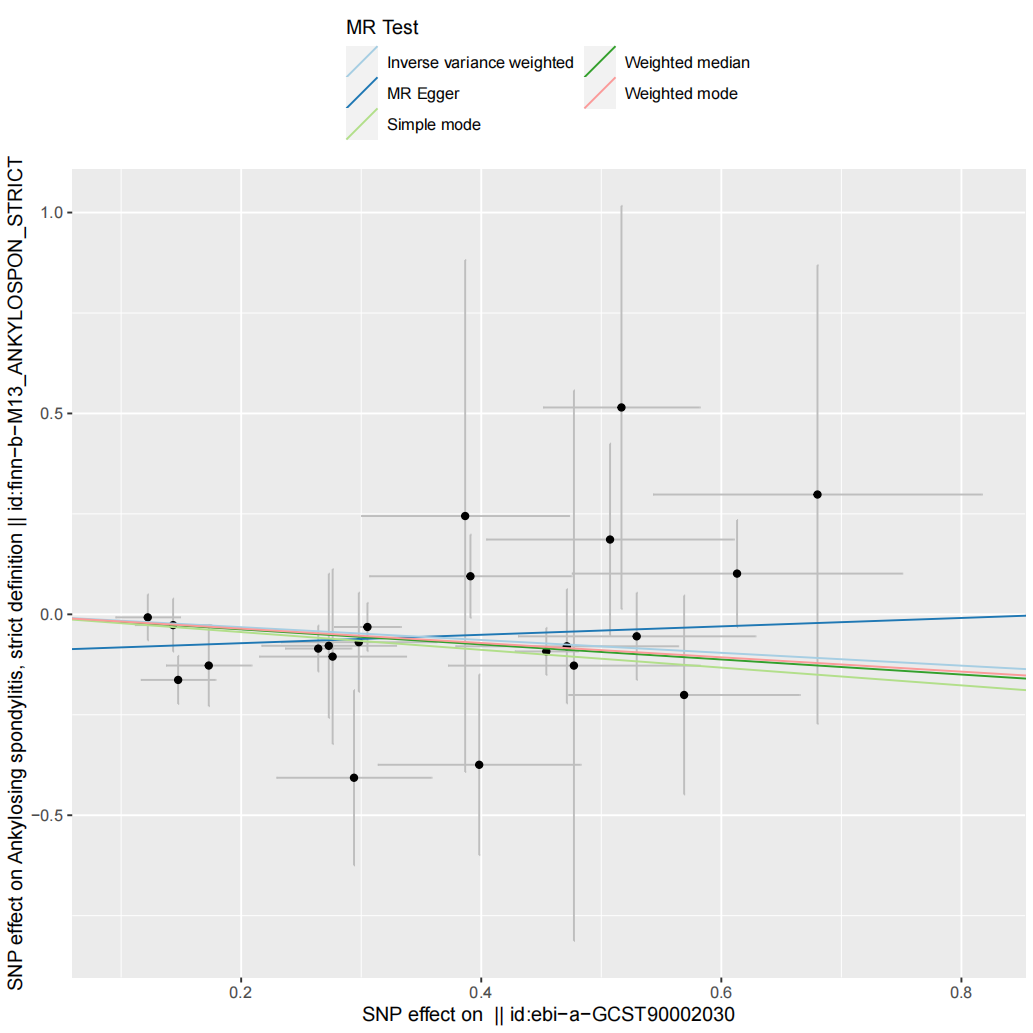

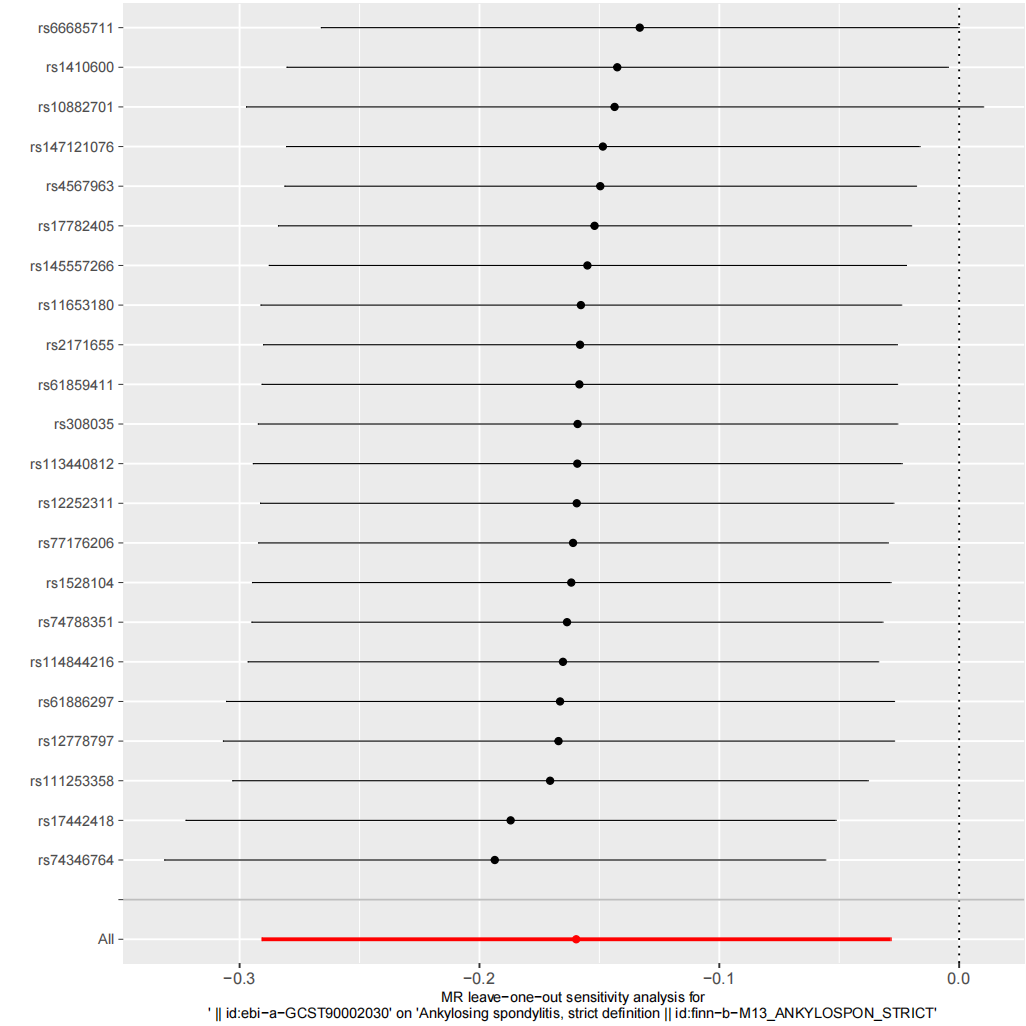


Supplementary Figure S1U Forest plot, funnel plot, scatter plot and sensitivity analysis of SNPs associated with CD39 on CD39+ activated Treg on AS.


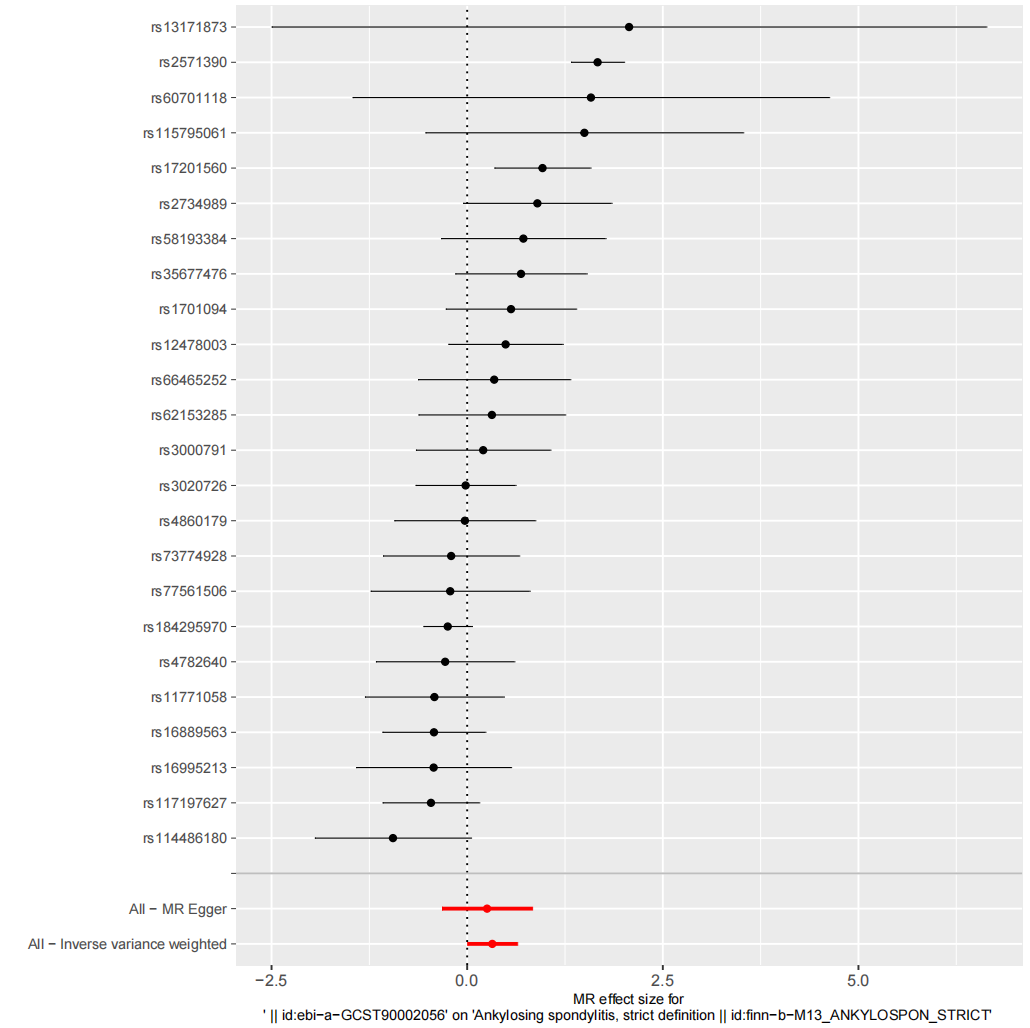

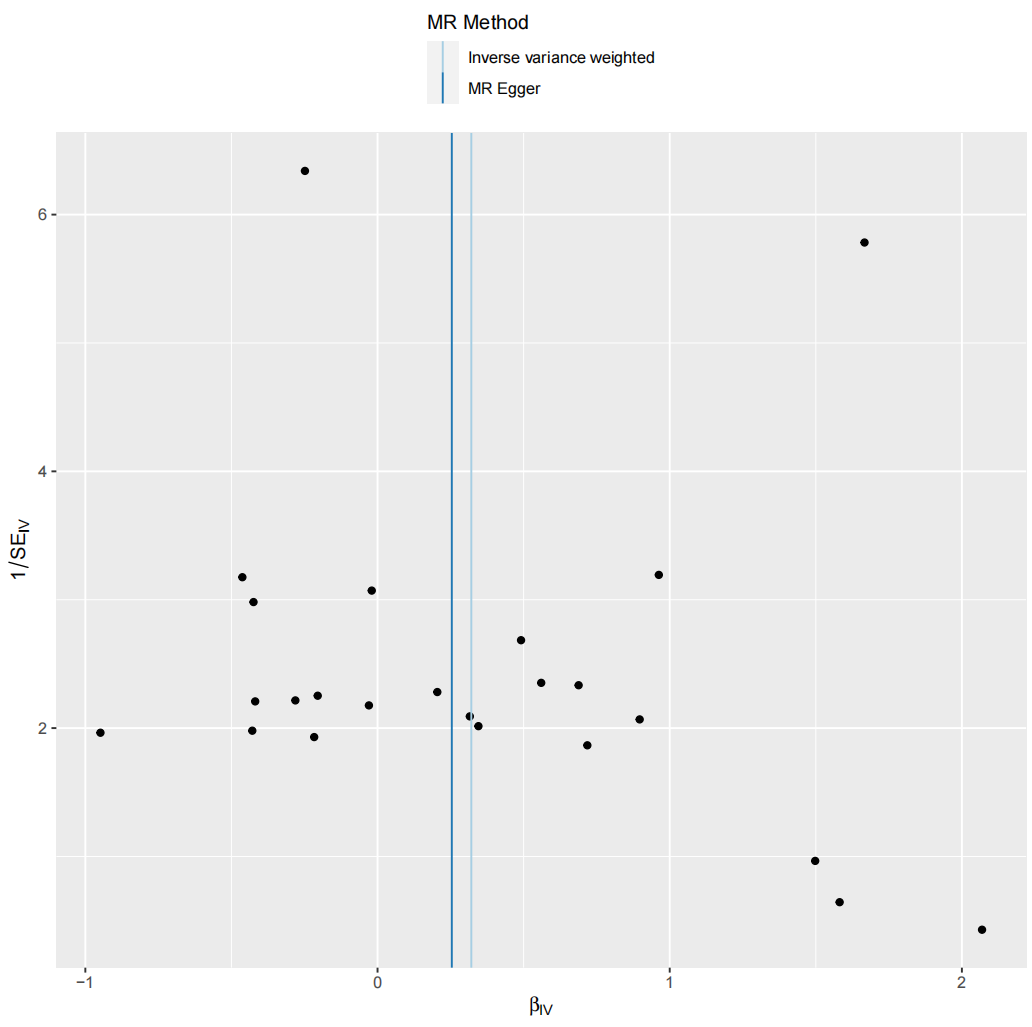

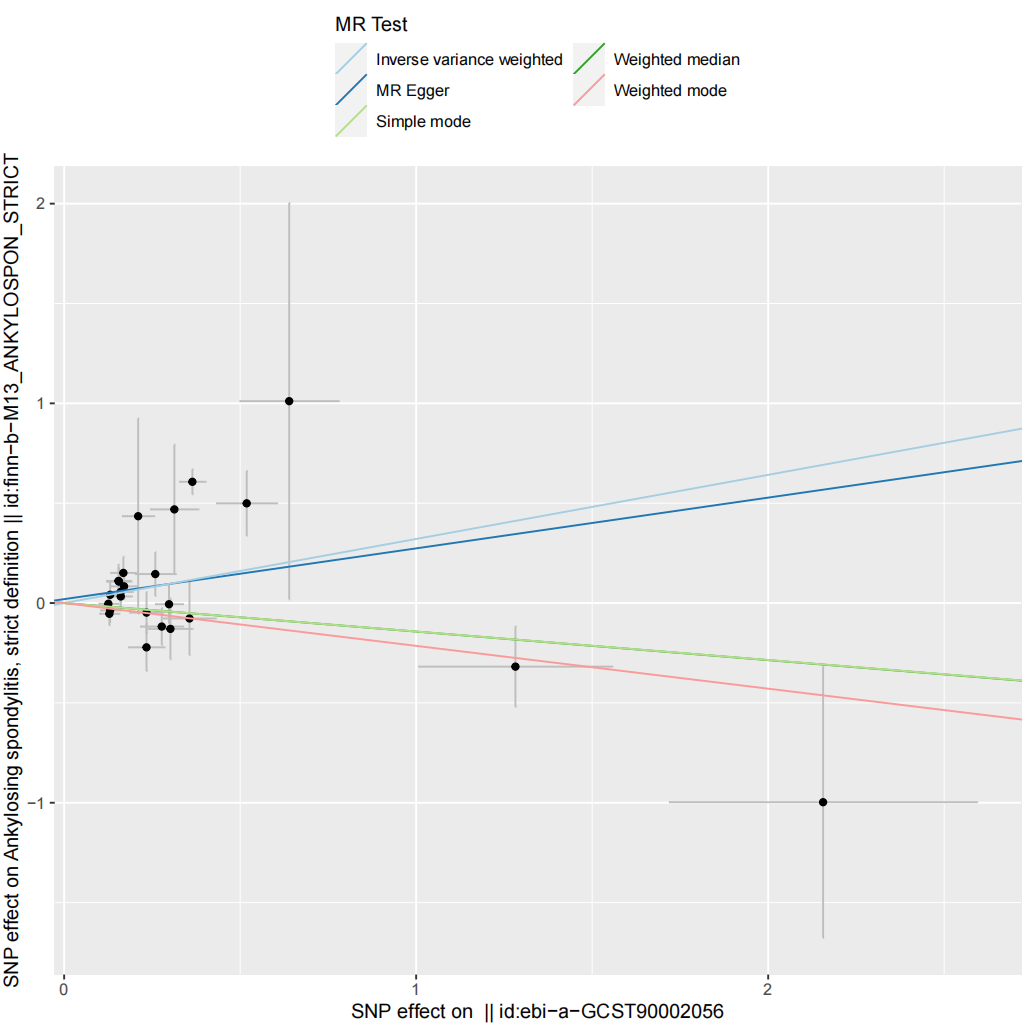

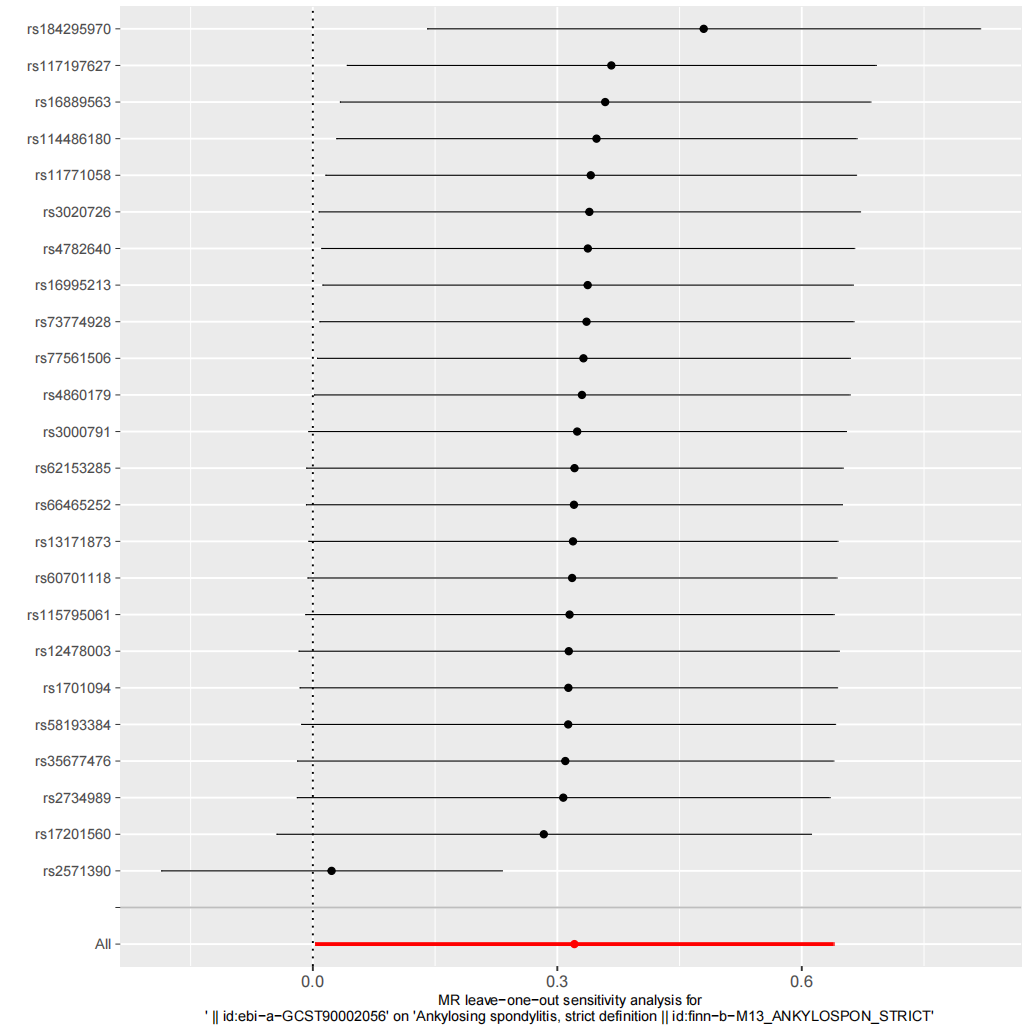


Supplementary Figure S1V Forest plot, funnel plot, scatter plot and sensitivity analysis of SNPs associated with CD8 on EM CD8br on AS.


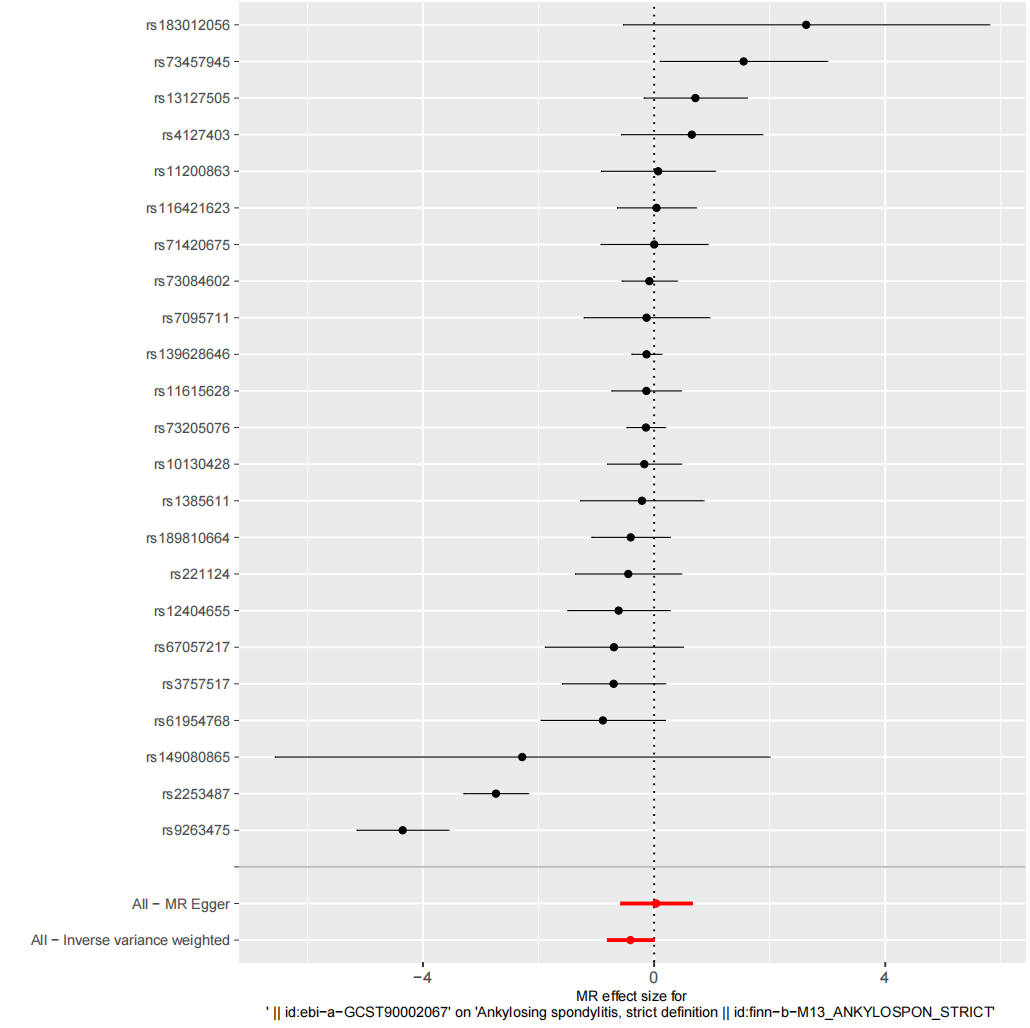

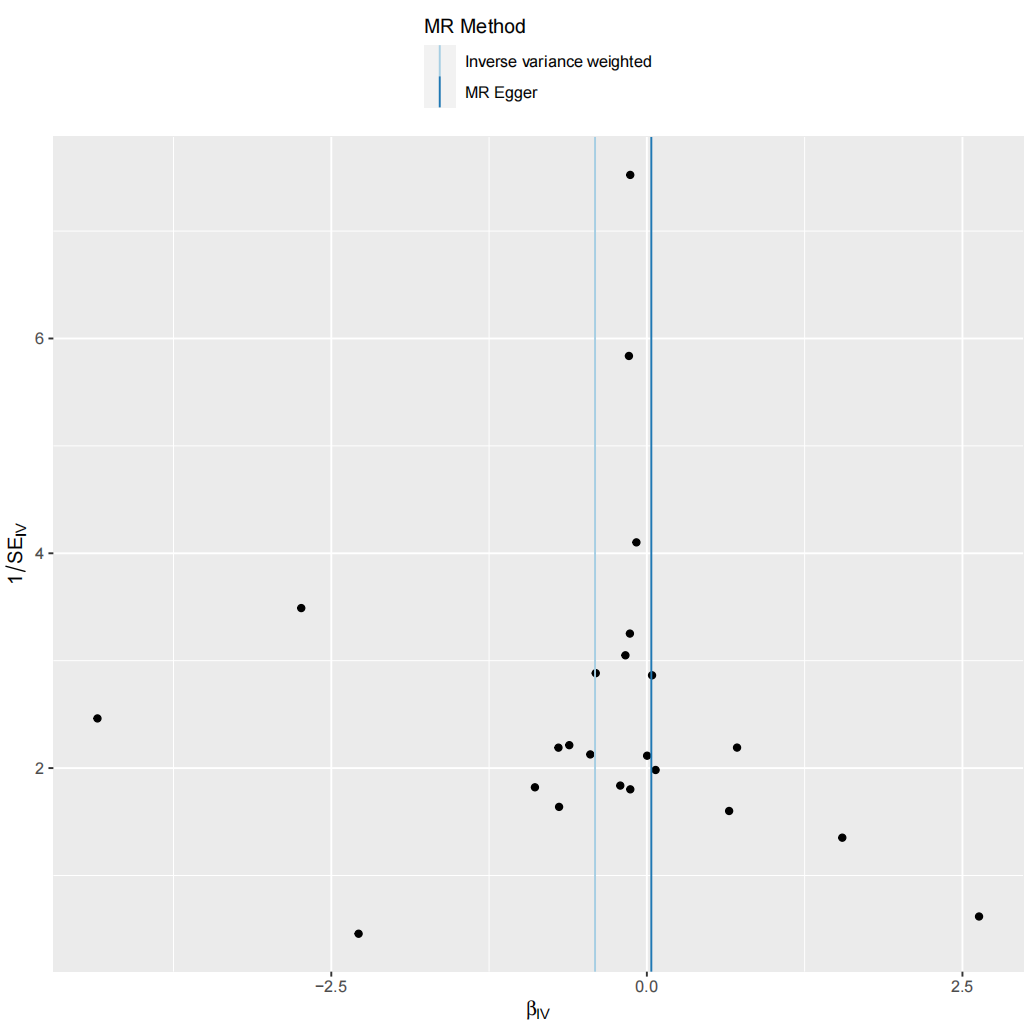

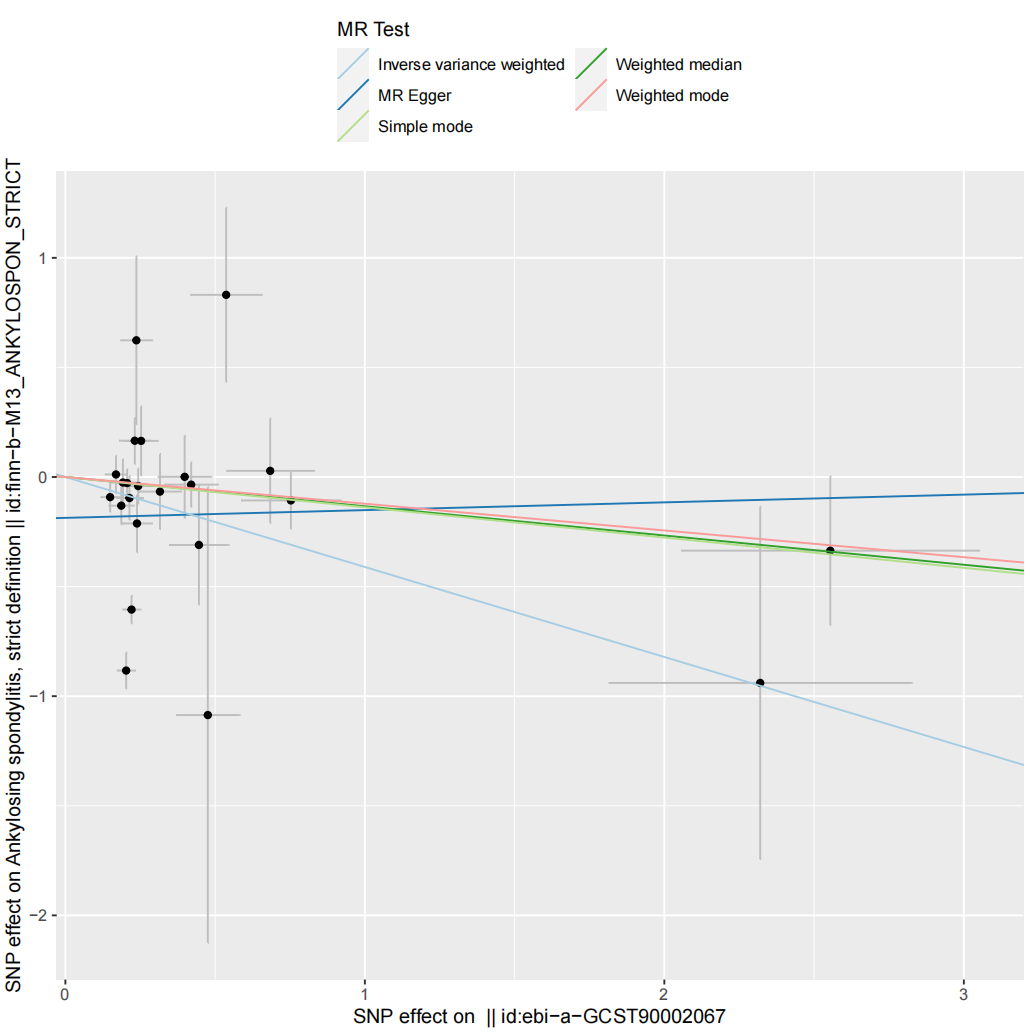

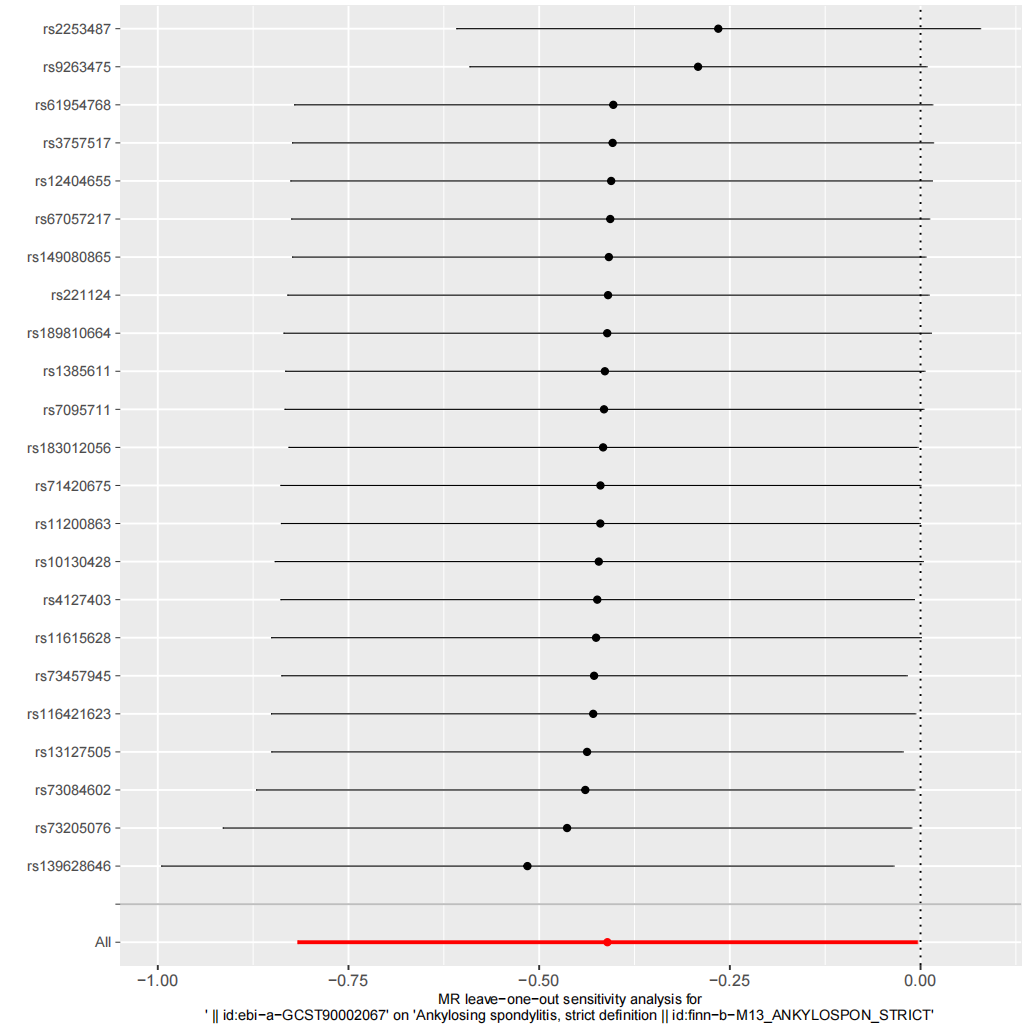


Supplementary Figure S1W Forest plot, funnel plot, scatter plot and sensitivity analysis of SNPs associated with CD4 on CD39+ activated Treg on AS.


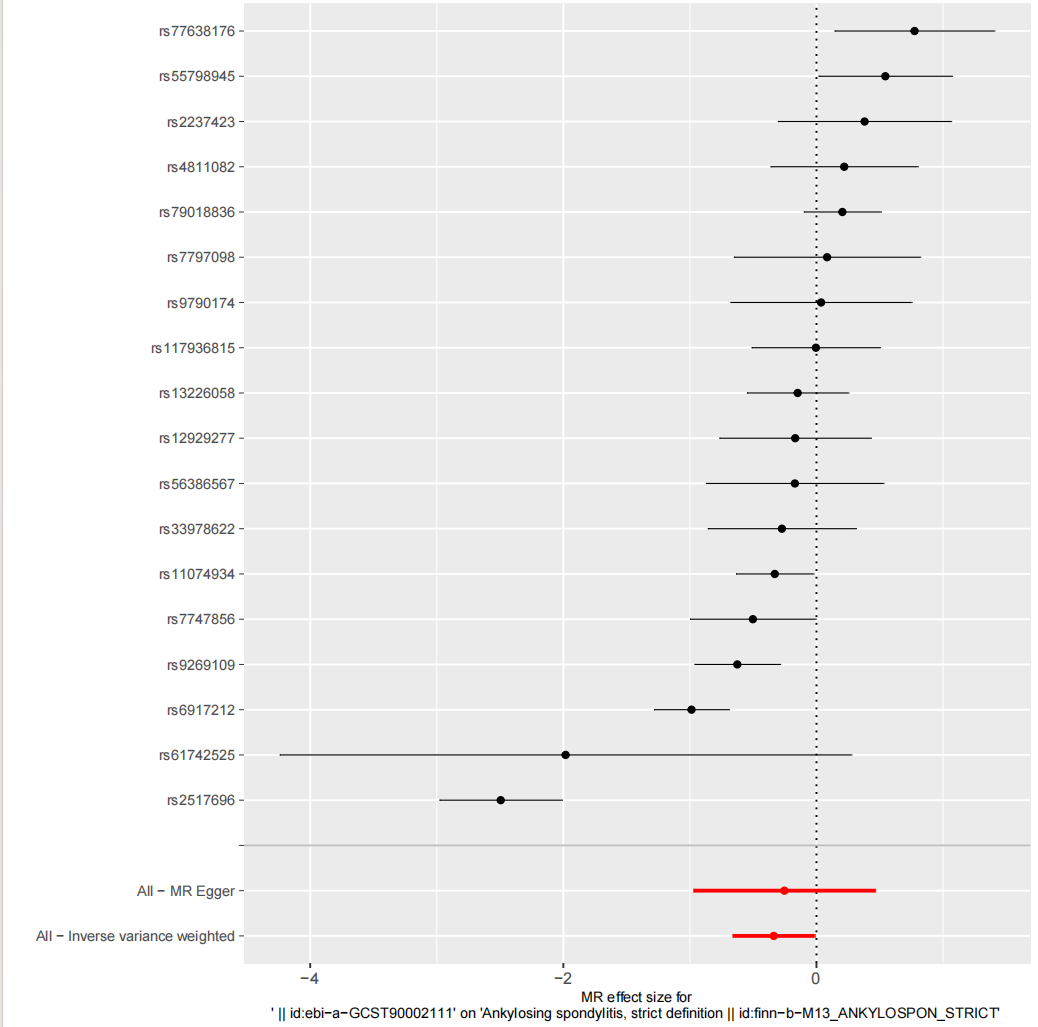

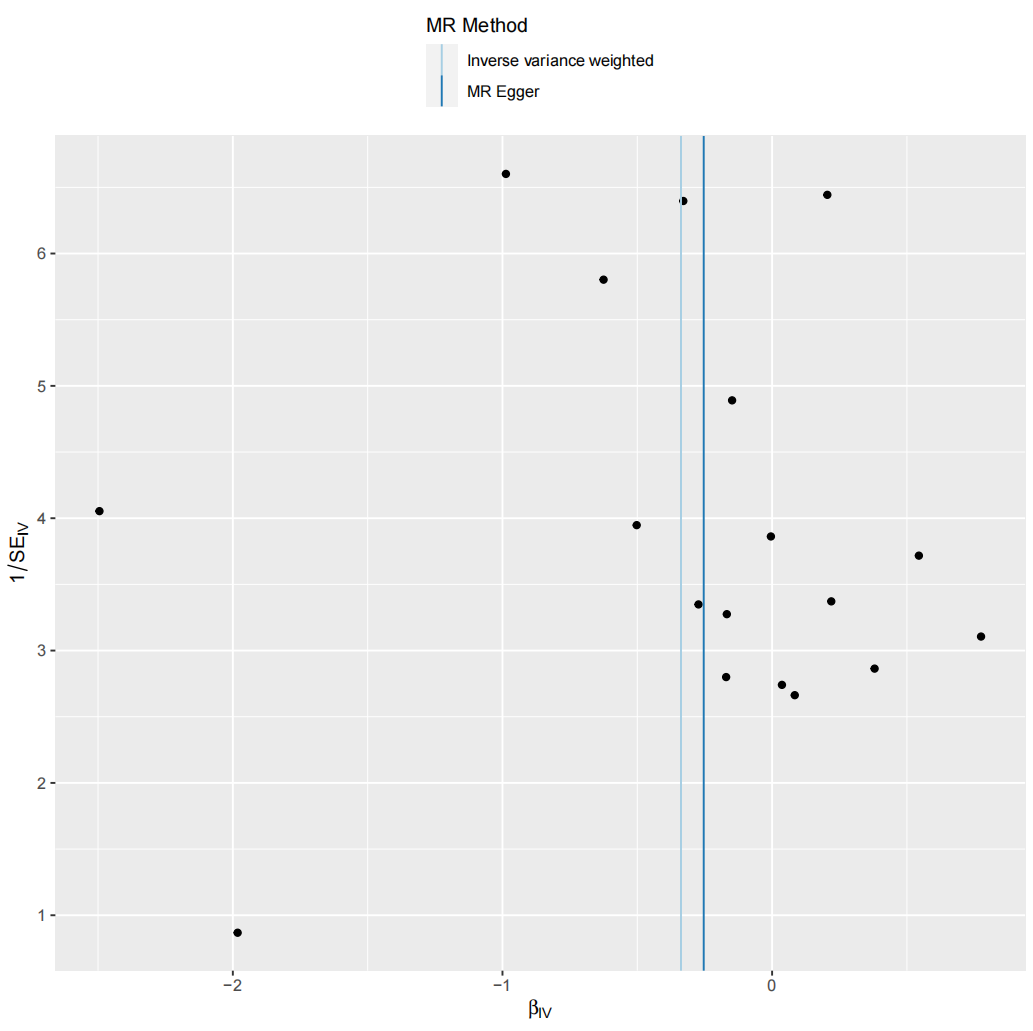


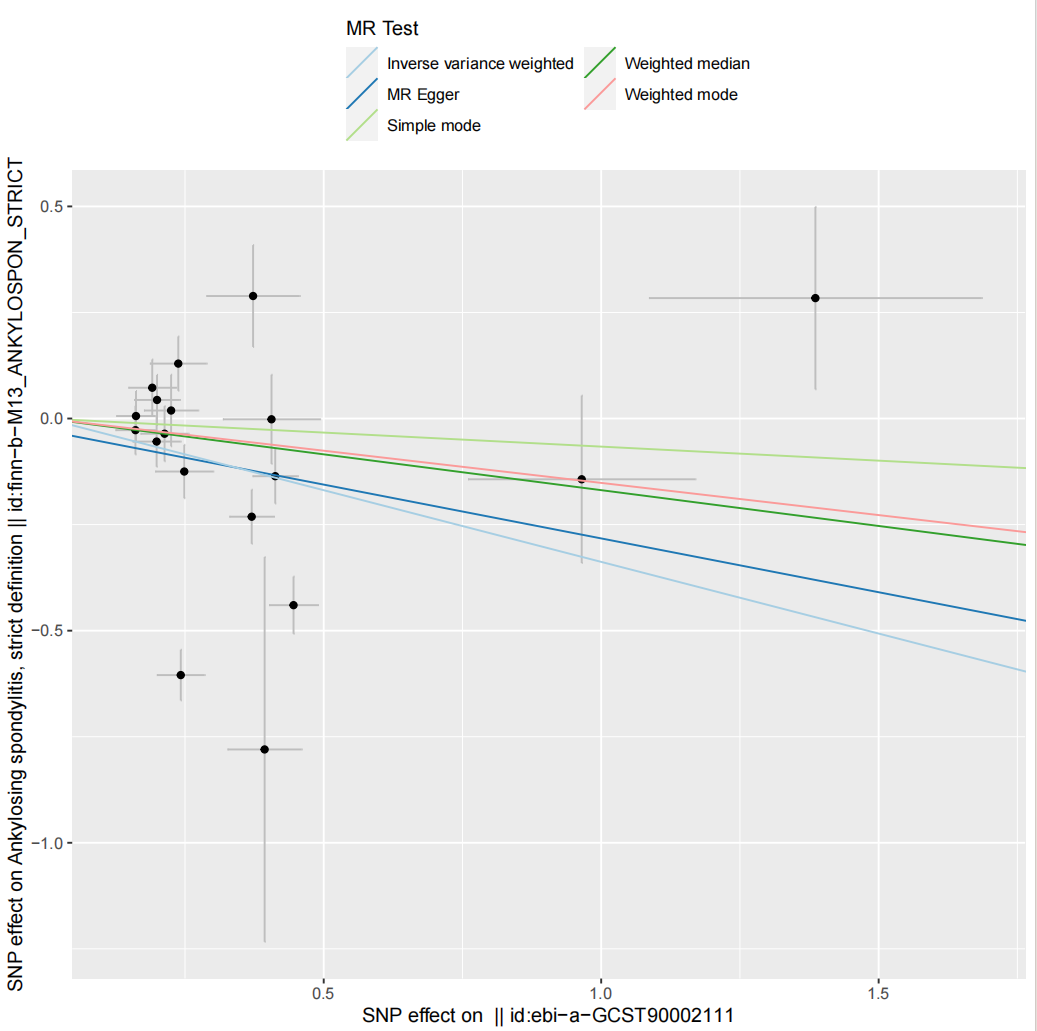

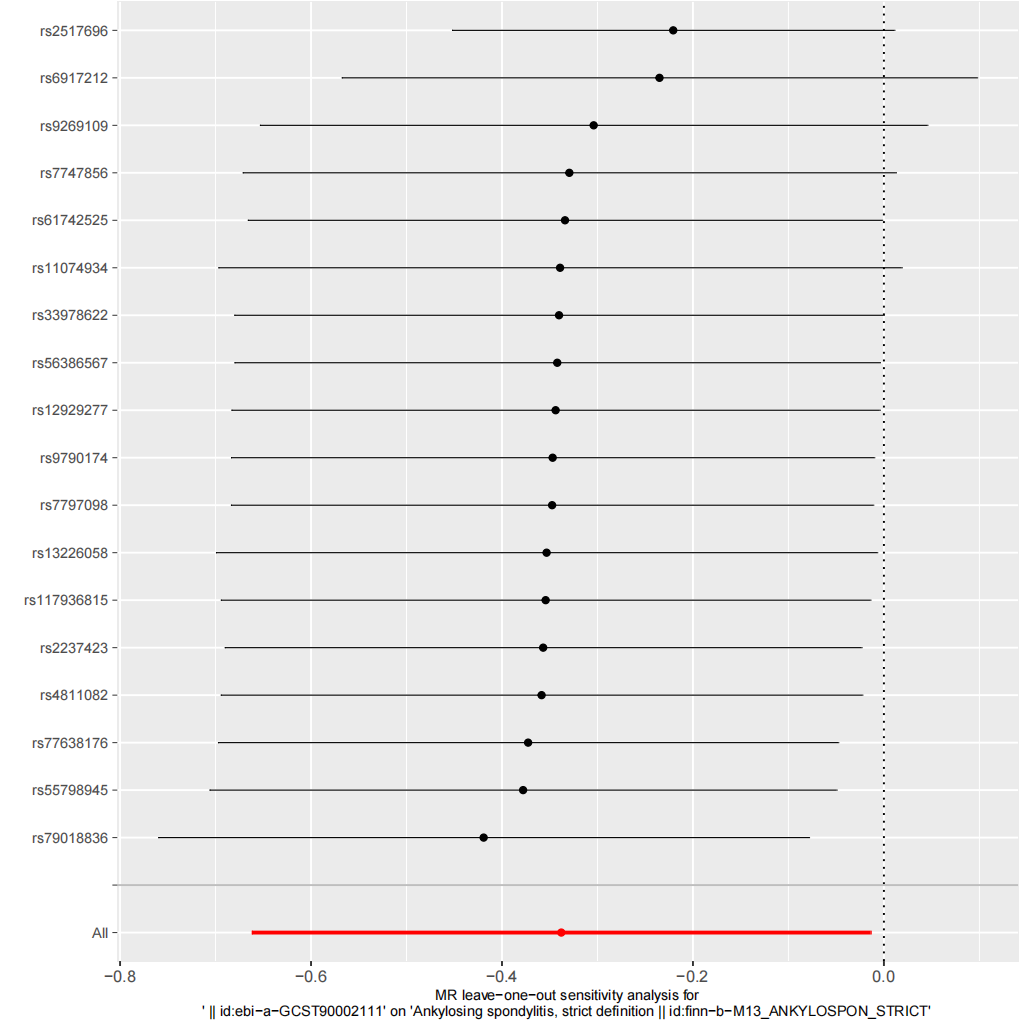


Supplementary Figure S1X Forest plot, funnel plot, scatter plot and sensitivity analysis of SNPs associated with HLA DR on CD33dim HLA DR+ CD11b- on AS.


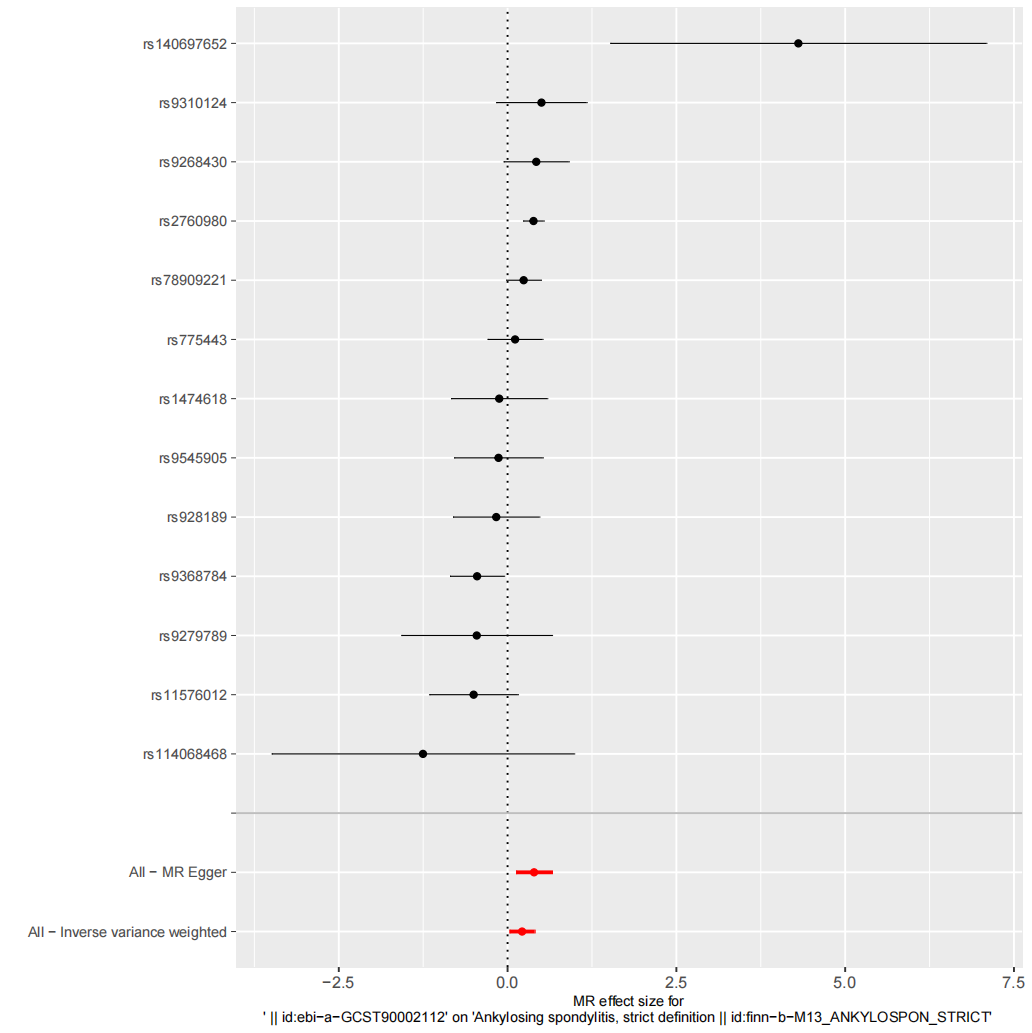

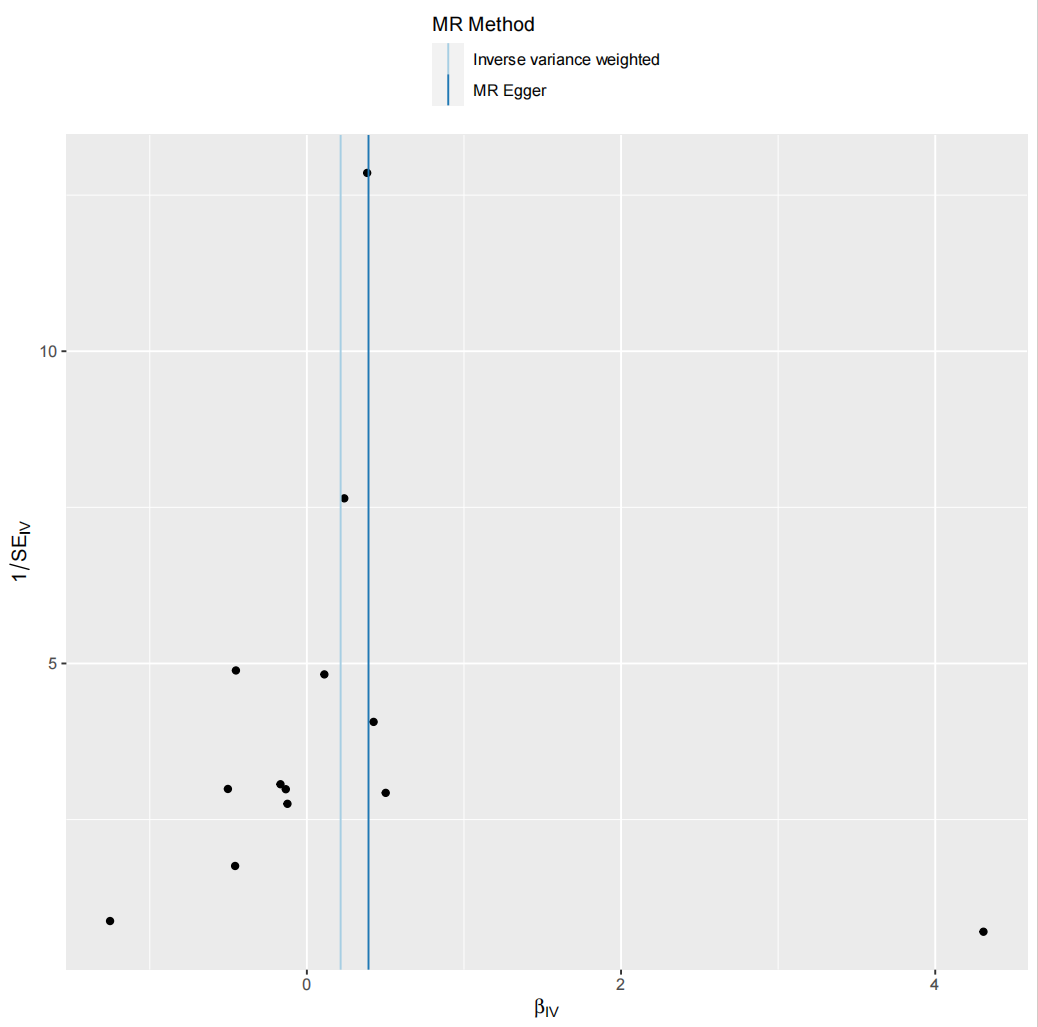

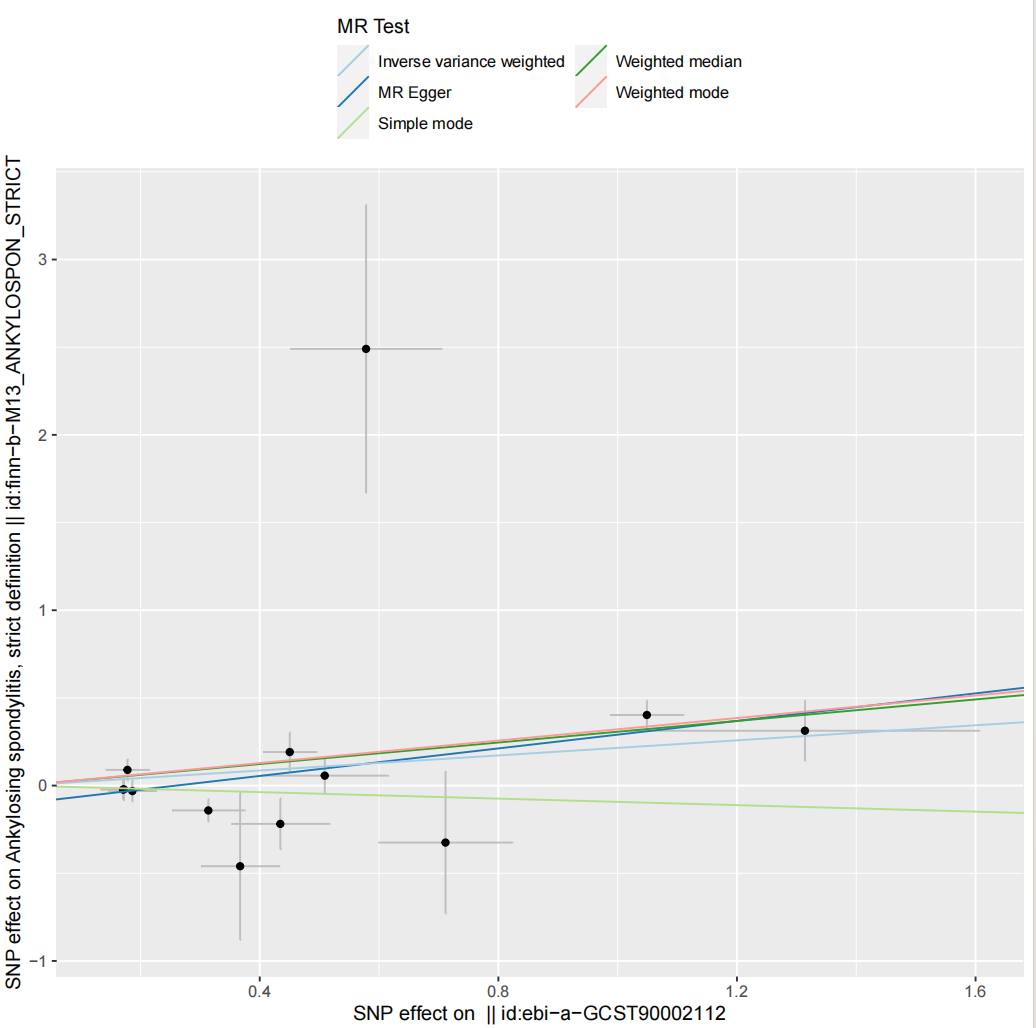

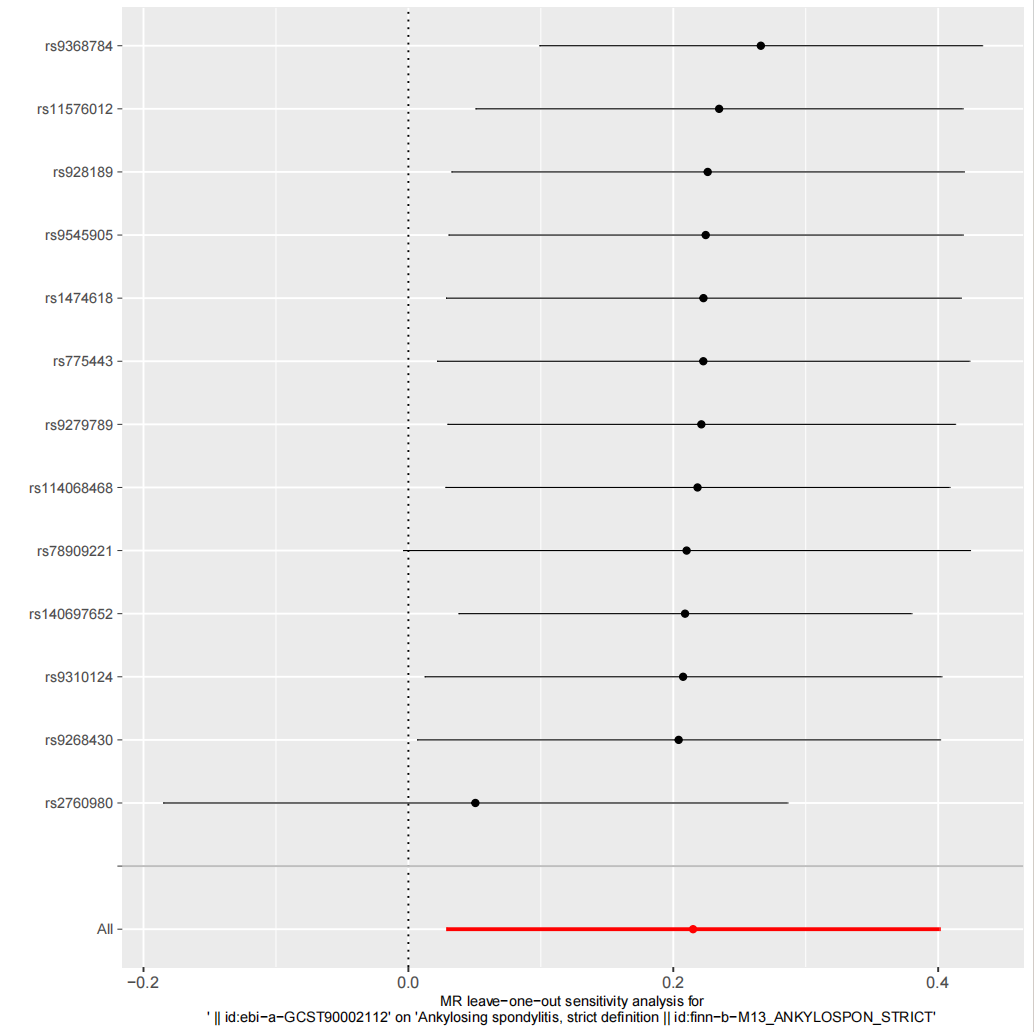


Supplementary Figure S1Y Forest plot, funnel plot, scatter plot and sensitivity analysis of SNPs associated with HLA DR on CD33- HLA DR+ on AS.

Supplementary Figure S1Z Forest plot, funnel plot, scatter plot and sensitivity analysis of SNPs associated with CD8 on CD28- CD8br on AS.
