# Supplementary material for: Mendelian randomization analysis links HLA-DR+ CD14− CD16+ monocytes to CCL19-driven ankylosing spondylitis risk
Source: Medicine (Baltimore). 2026 May 8;105(19):e48687. doi: 10.1097/MD.0000000000048687 (PMC13166580; doi:10.1097/MD.0000000000048687)
Supplement: Supplementary file 7 [file medi-105-e48687-s007.doc]

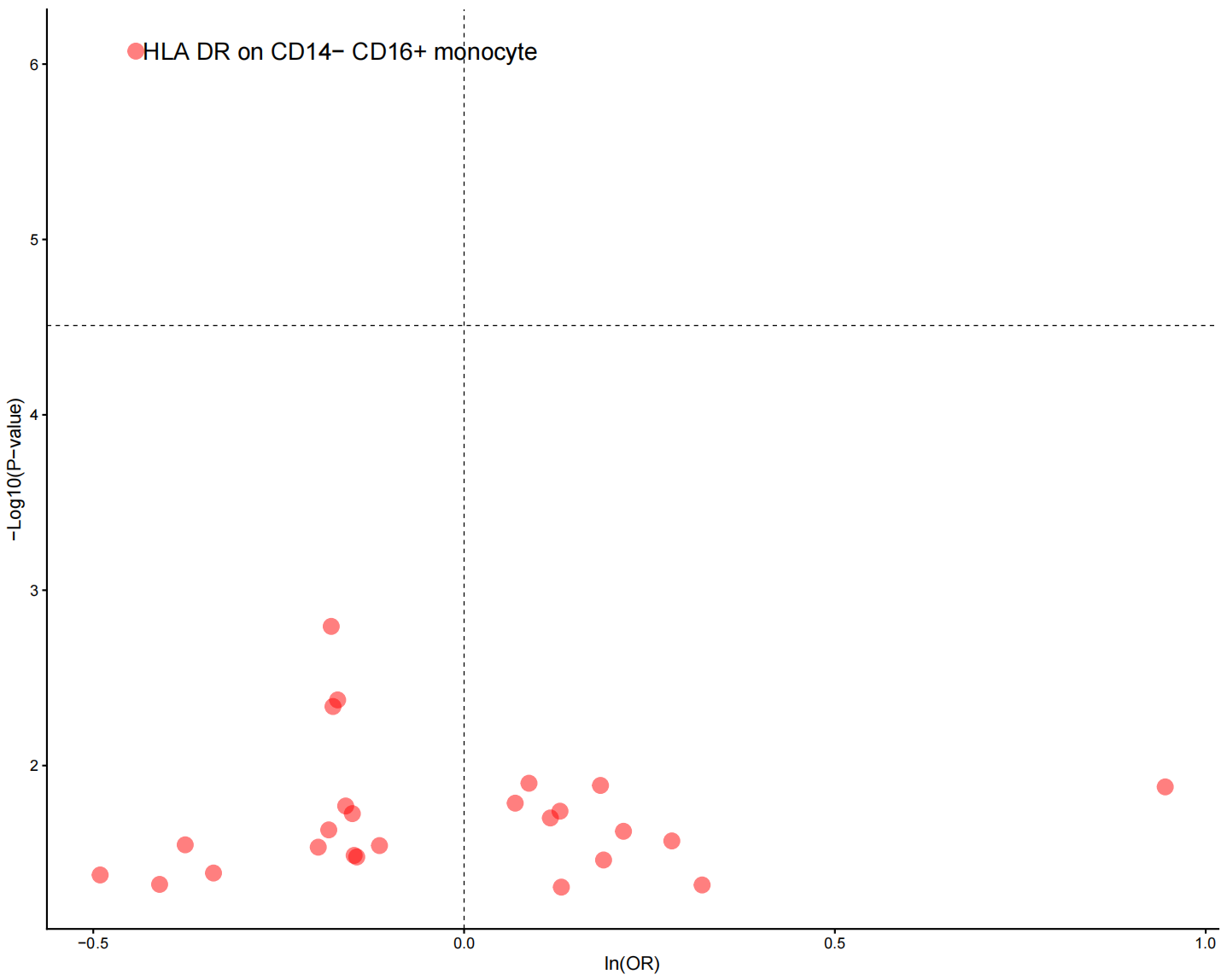


**Supplementary Figure S4. Volcano plot of causal effects of all 731 immune cell phenotypes on AS risk.**

Each point represents one immune cell phenotype. The x-axis shows the causal effect size (β coefficient from IVW method), and the y-axis shows the statistical significance (-log₁₀ P-value). The horizontal dashed line indicates the nominal significance threshold (P = 0.05). The vertical dashed line indicates the null effect (β = 0). Phenotypes to the left of the vertical line (negative β) are protective, while those to the right (positive β) are risk factors. The red point highlights "HLA DR on CD14- CD16+ monocyte," which shows a strong protective effect with high statistical significance.
