# Supplementary material for: Mendelian randomization analysis links HLA-DR+ CD14− CD16+ monocytes to CCL19-driven ankylosing spondylitis risk
Source: Medicine (Baltimore). 2026 May 8;105(19):e48687. doi: 10.1097/MD.0000000000048687 (PMC13166580; doi:10.1097/MD.0000000000048687)
Supplement: Supplementary file 10 [file medi-105-e48687-s010.doc]

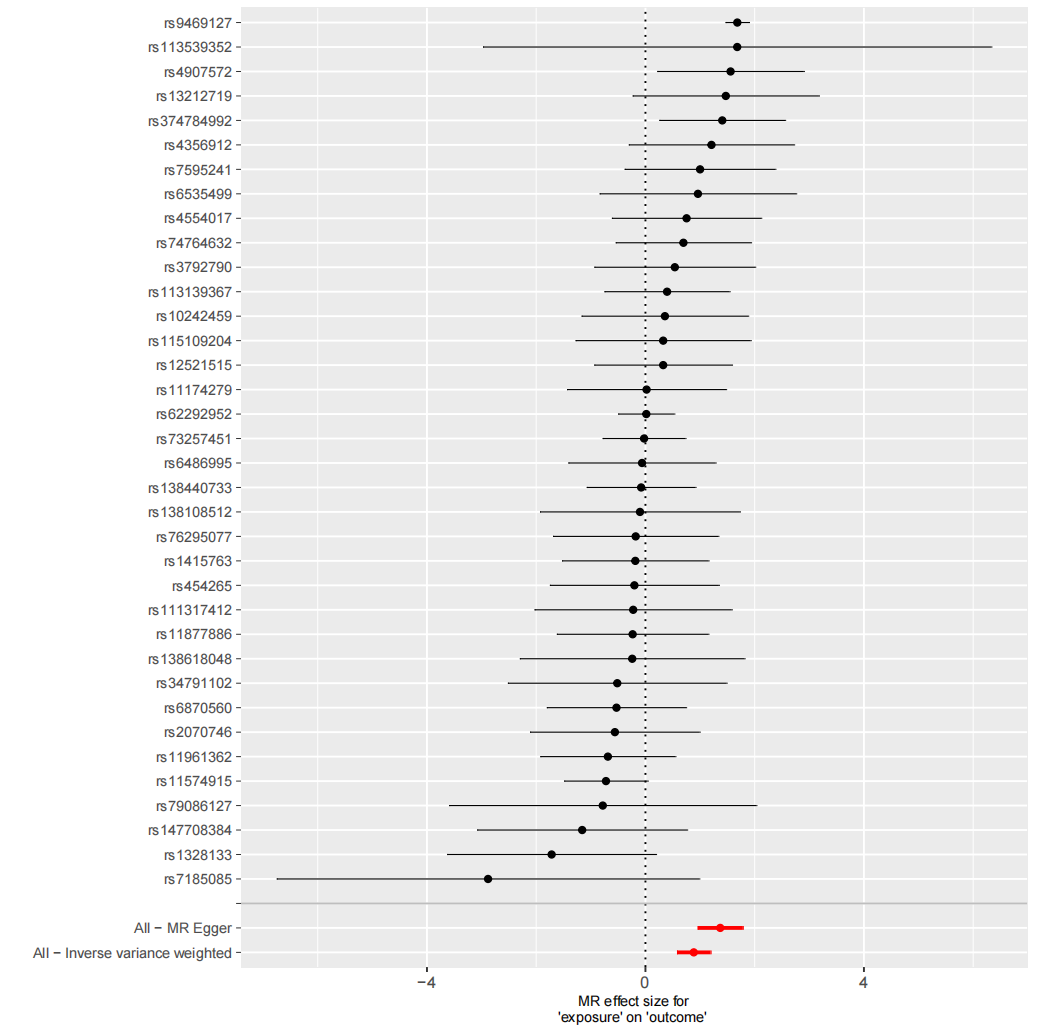

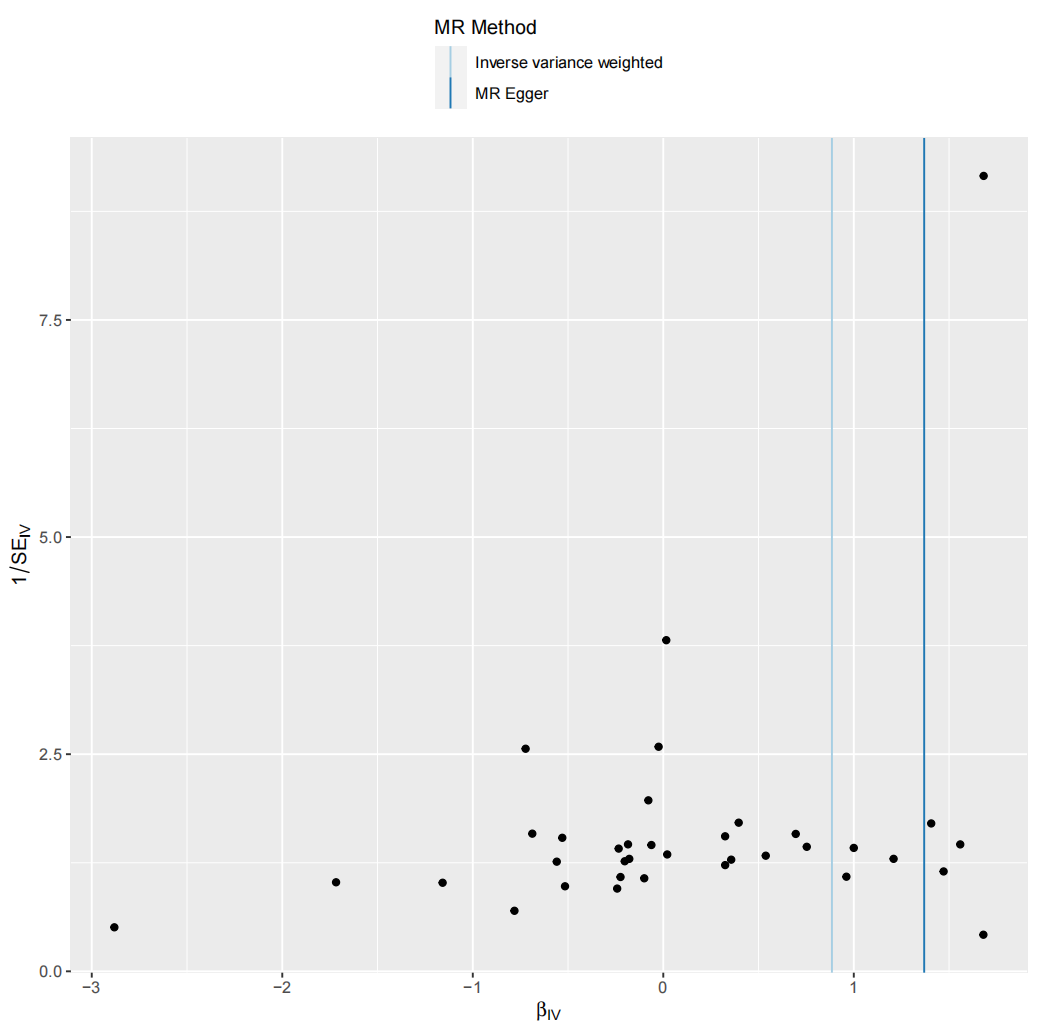

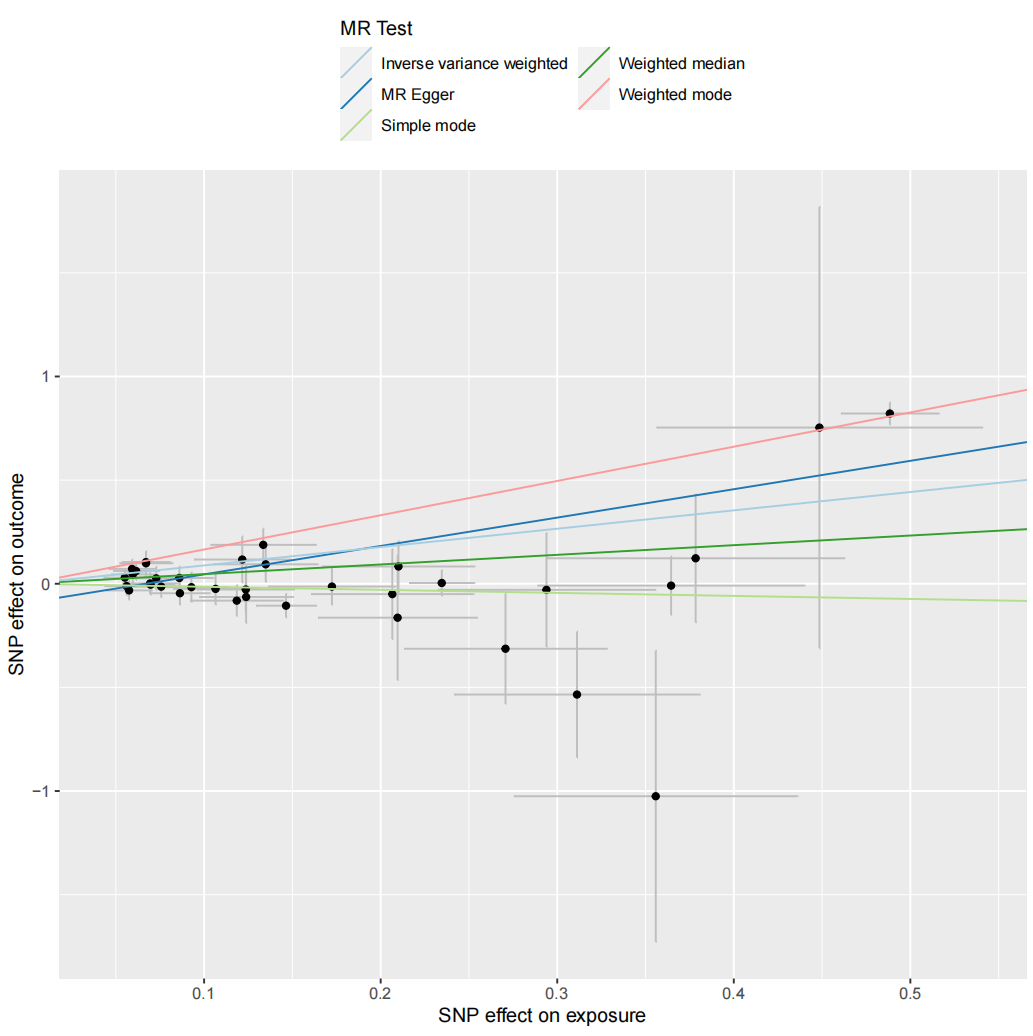

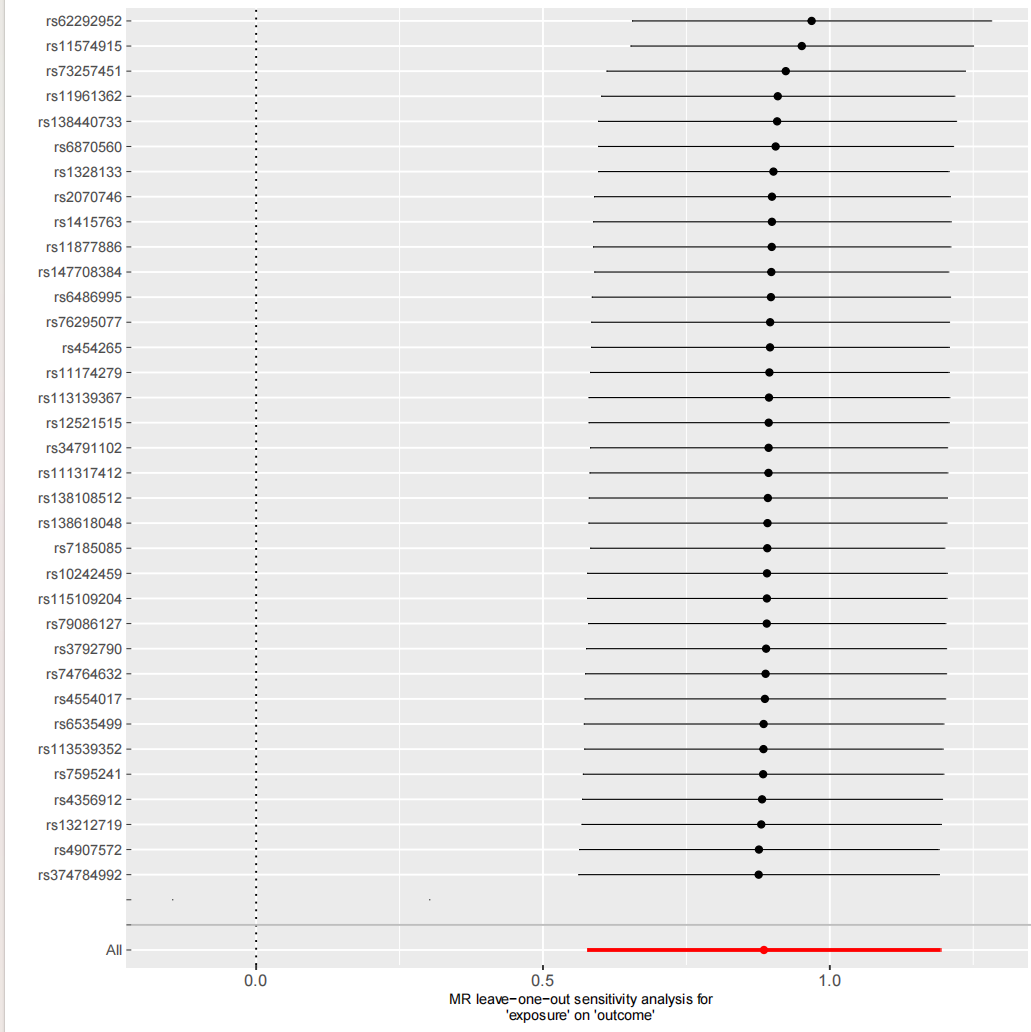


Supplementary Figure S2 Forest plot, funnel plot, scatter plot and sensitivity analysis of SNPs associated with C-C motif chemokine 19 levels on AS.


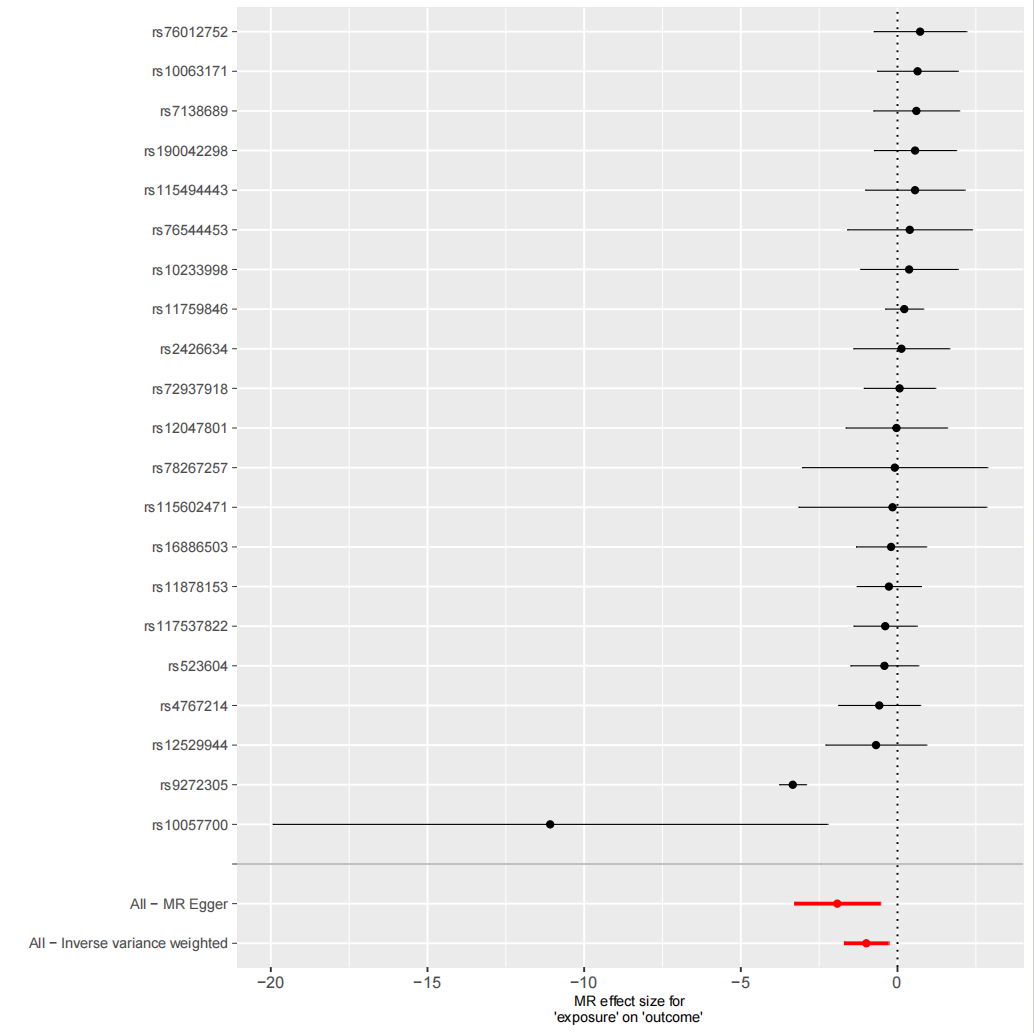

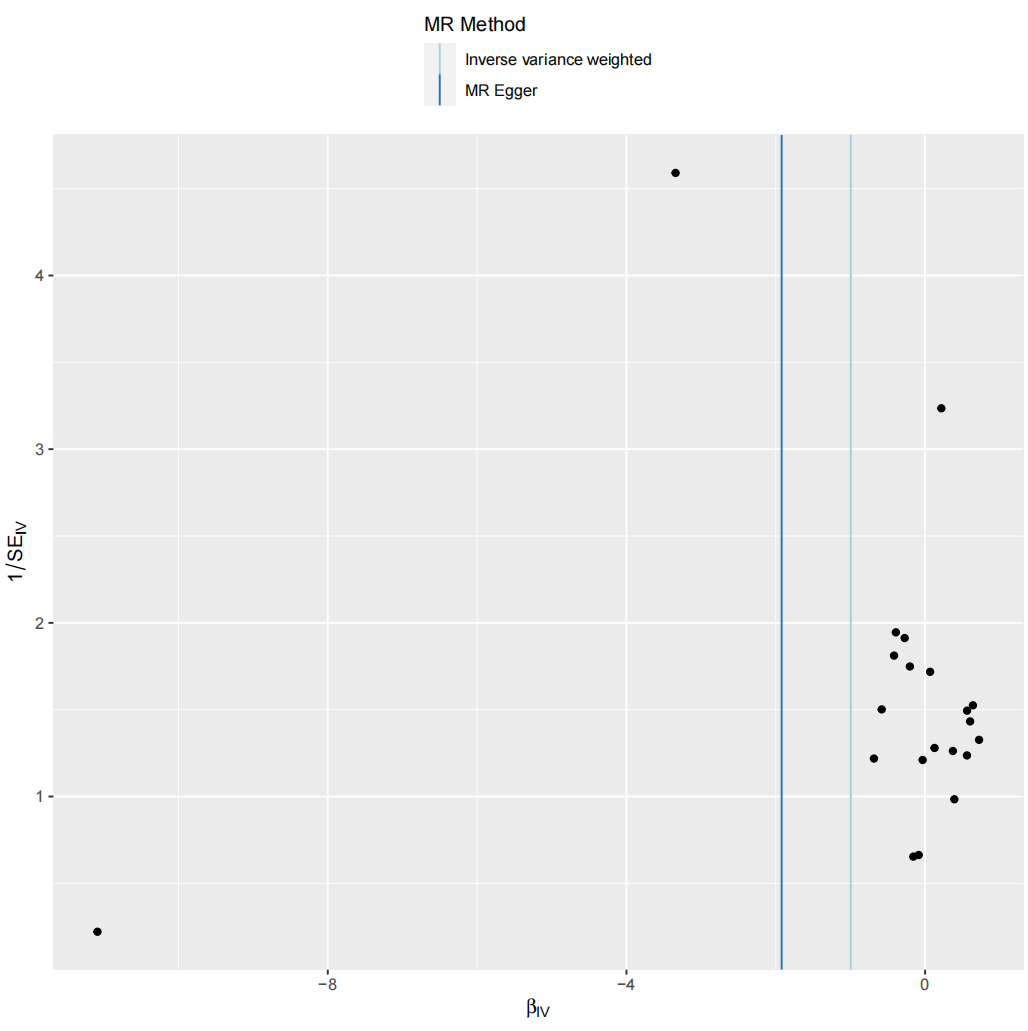

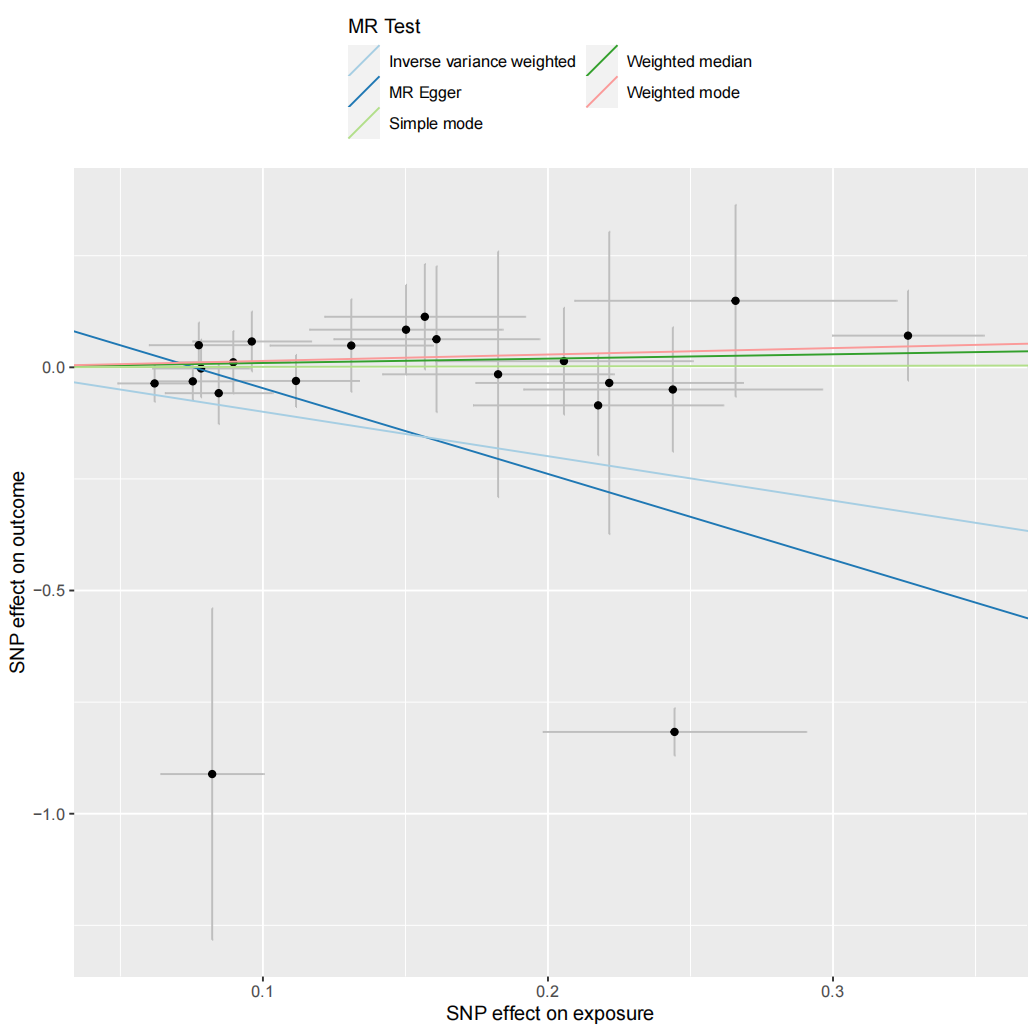

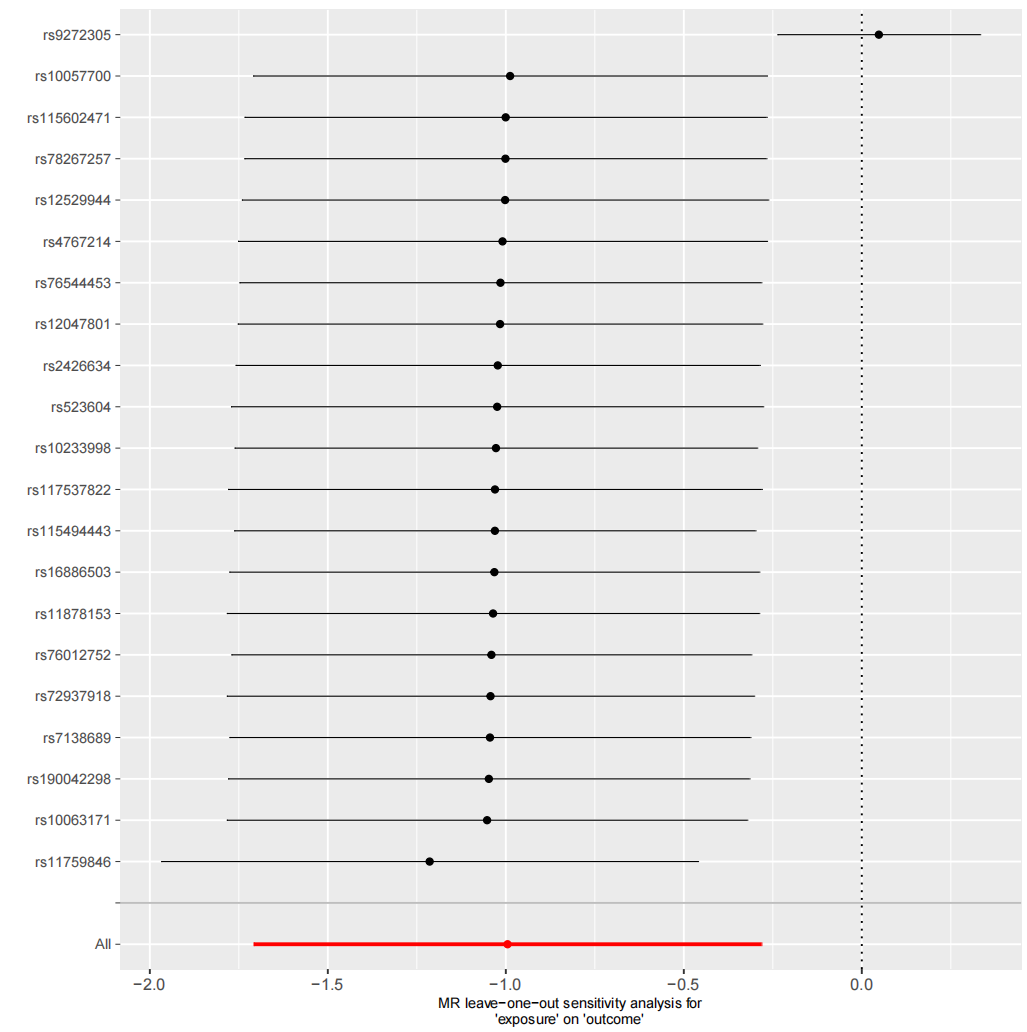


Supplementary Figure S2B Forest plot, funnel plot, scatter plot and sensitivity analysis of SNPs associated with Interleukin-1-alpha levels on AS.


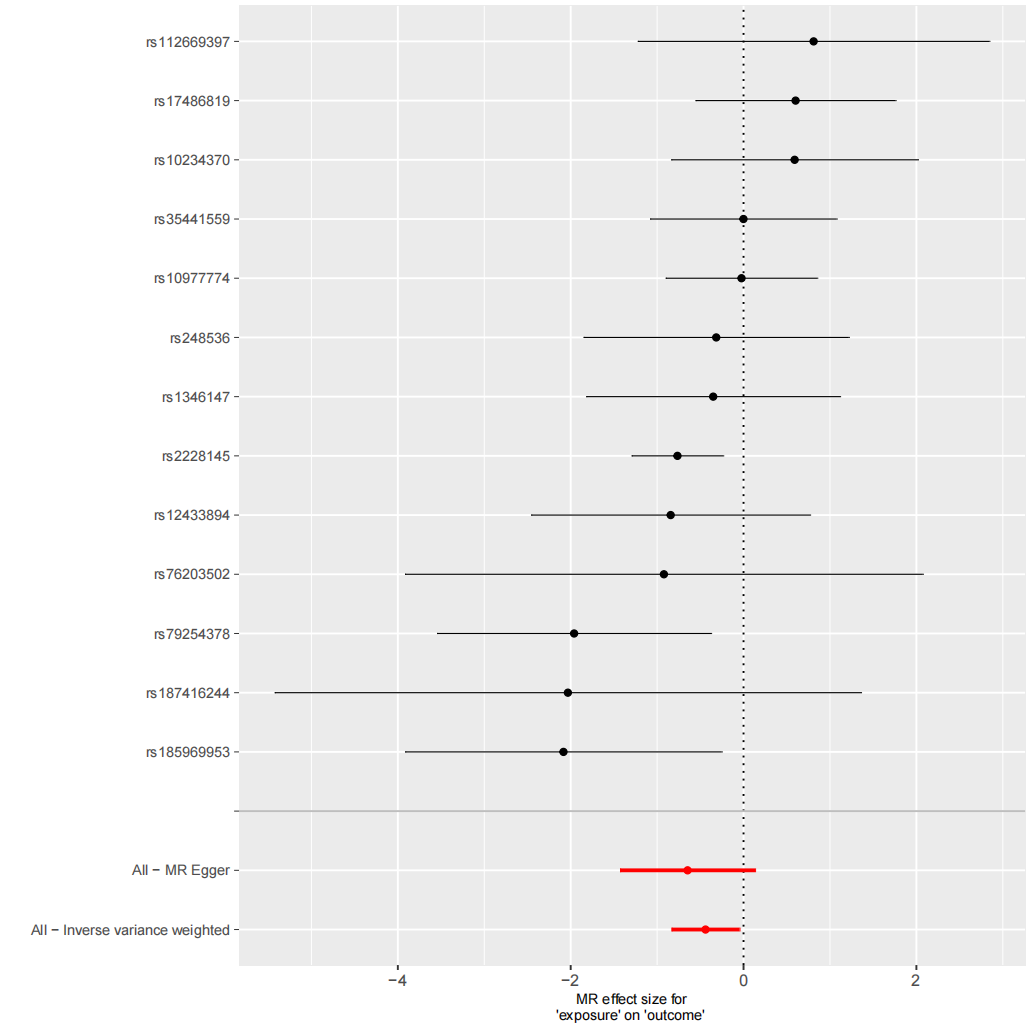

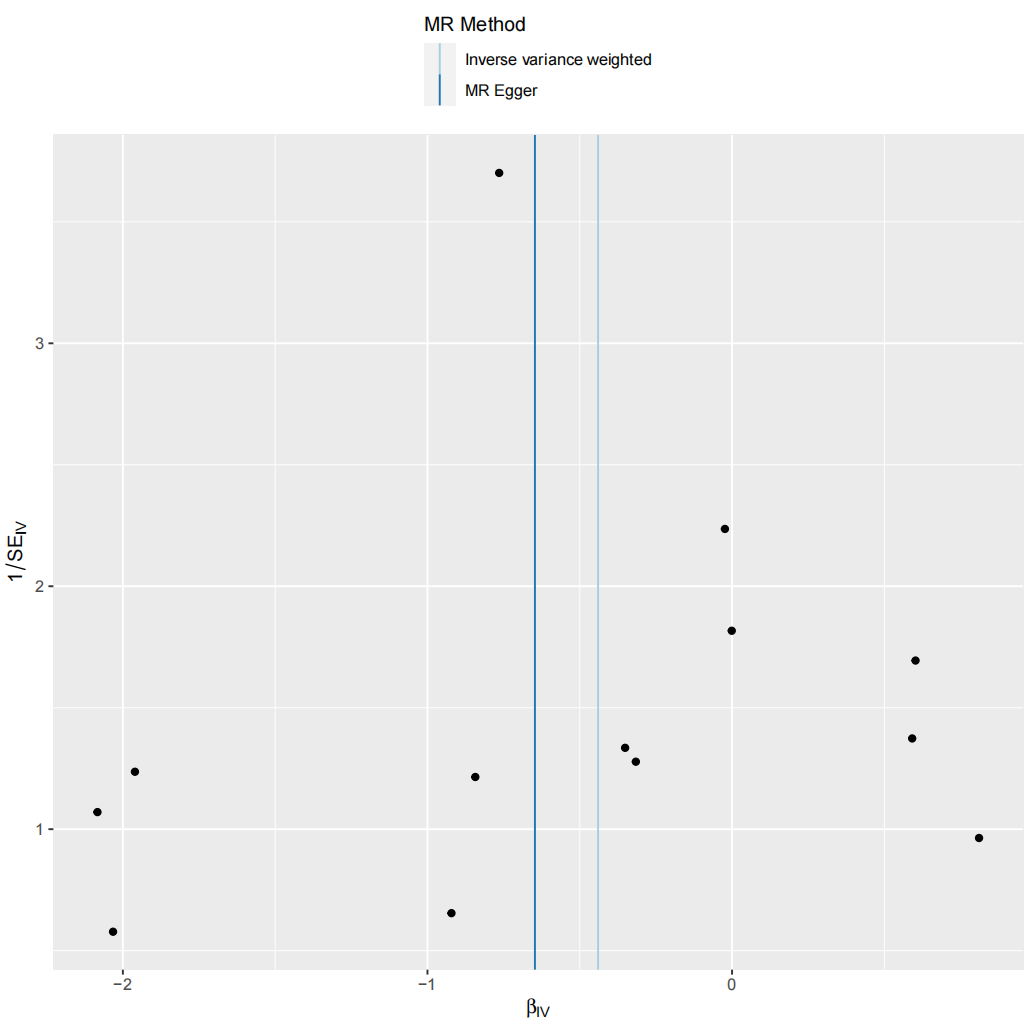


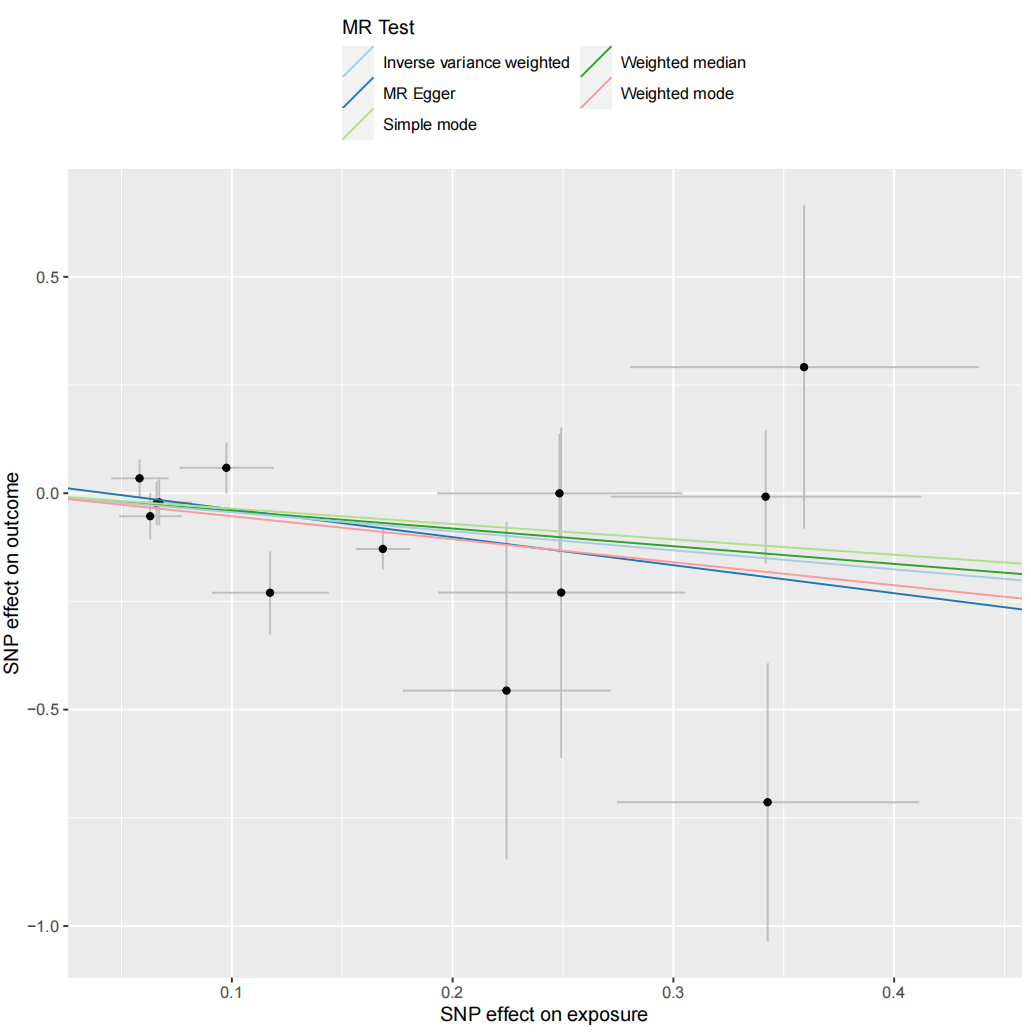

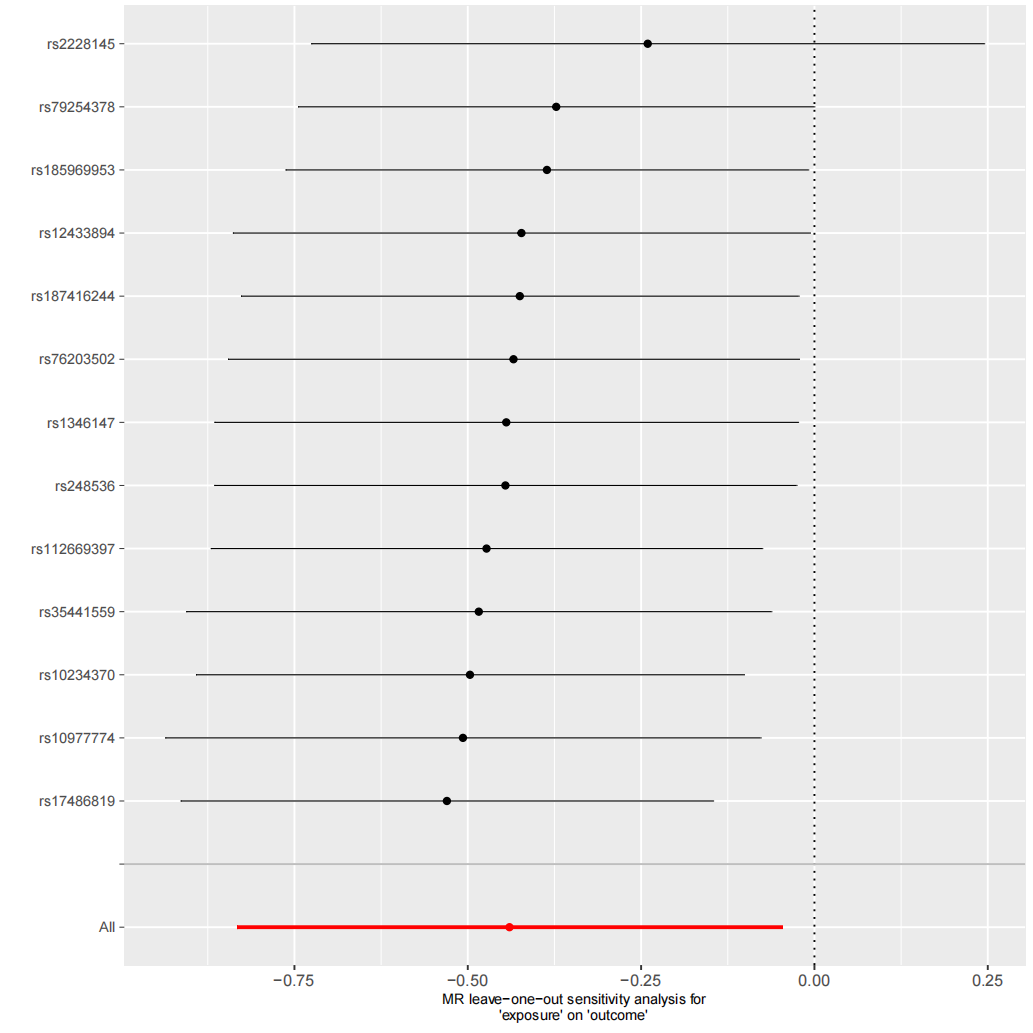


Supplementary Figure S2C Forest plot, funnel plot, scatter plot and sensitivity analysis of SNPs associated with Interleukin-6 levels on AS.


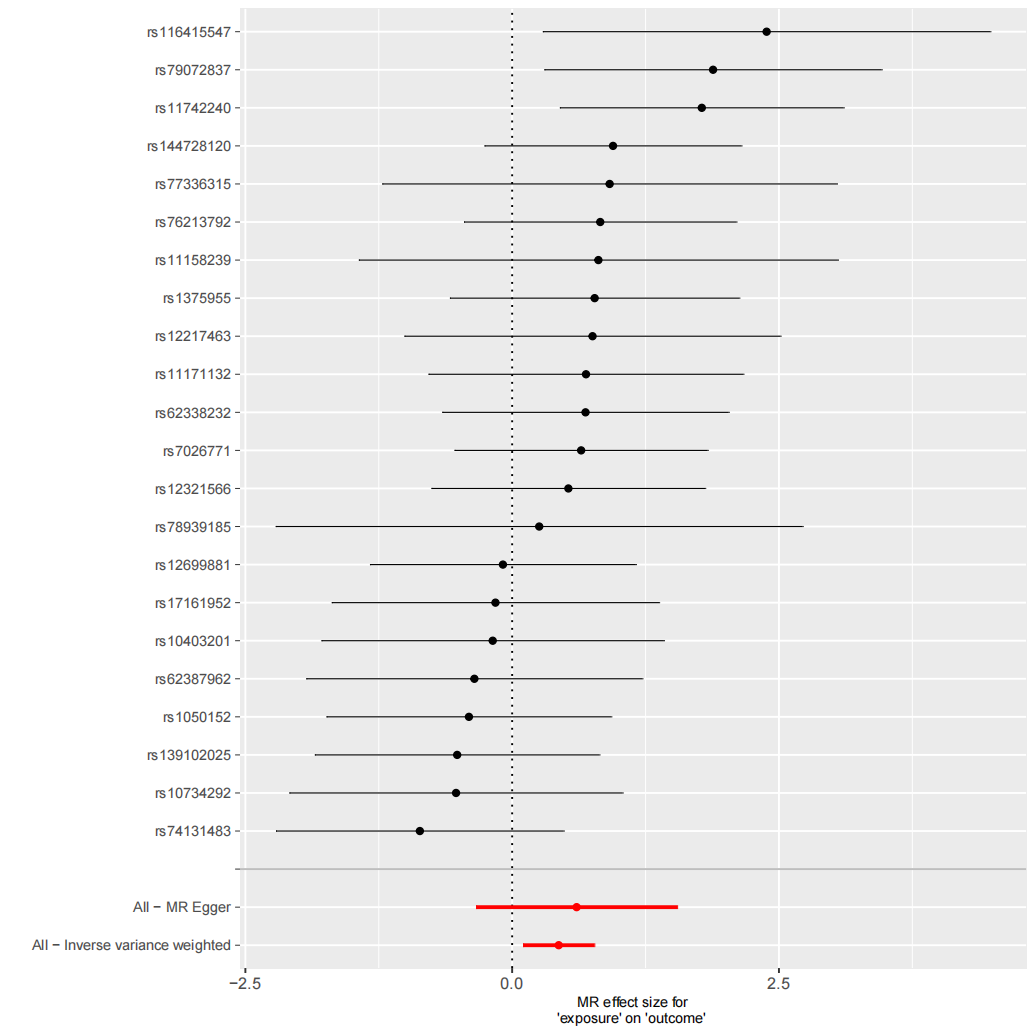

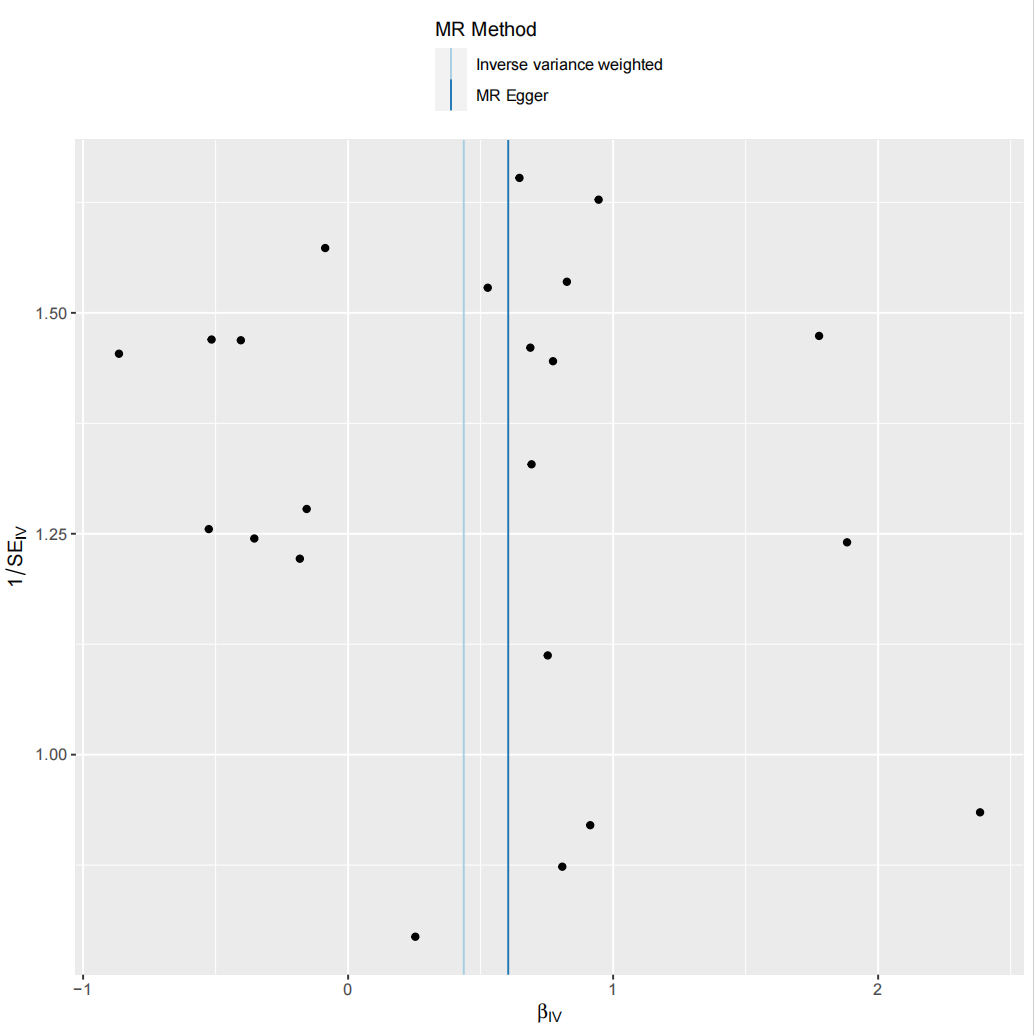

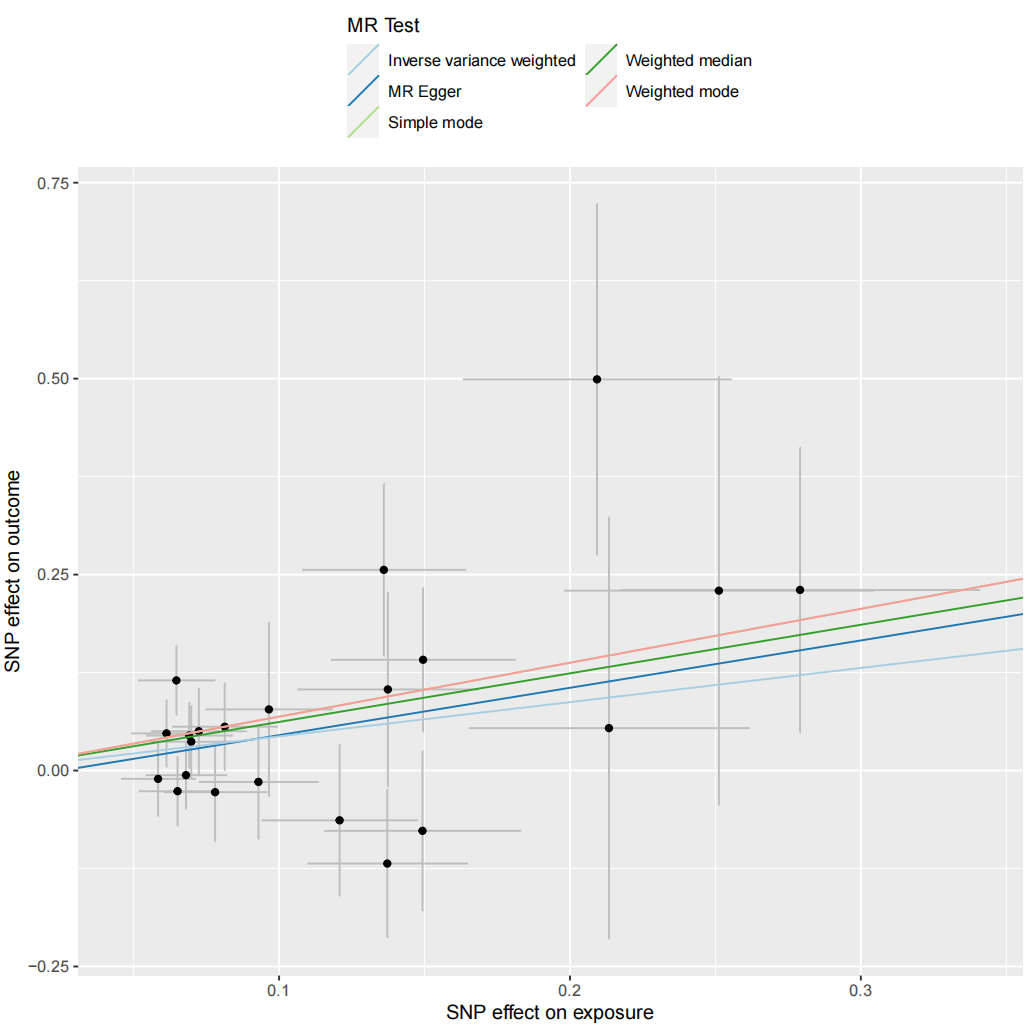

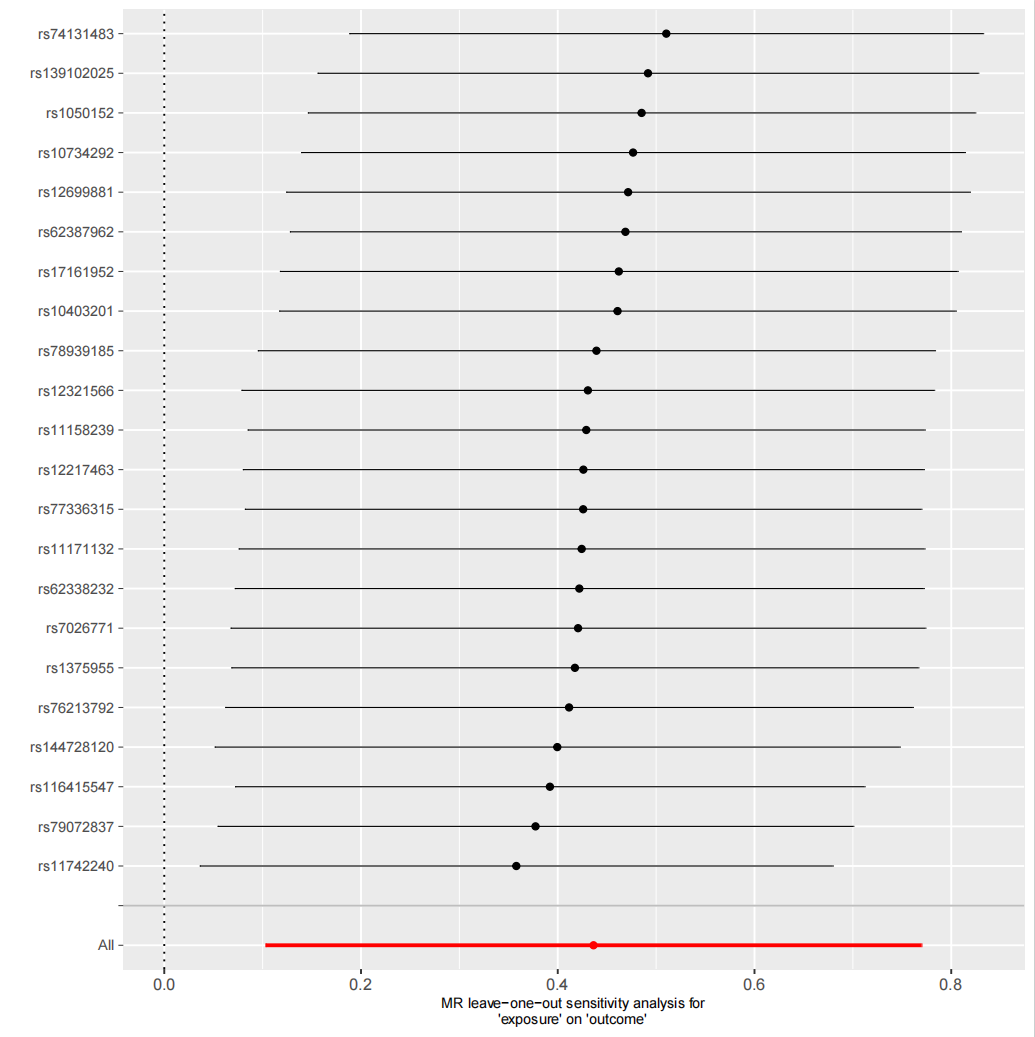


Supplementary Figure S2D Forest plot, funnel plot, scatter plot and sensitivity analysis of SNPs associated with Interleukin-7 levels on AS.


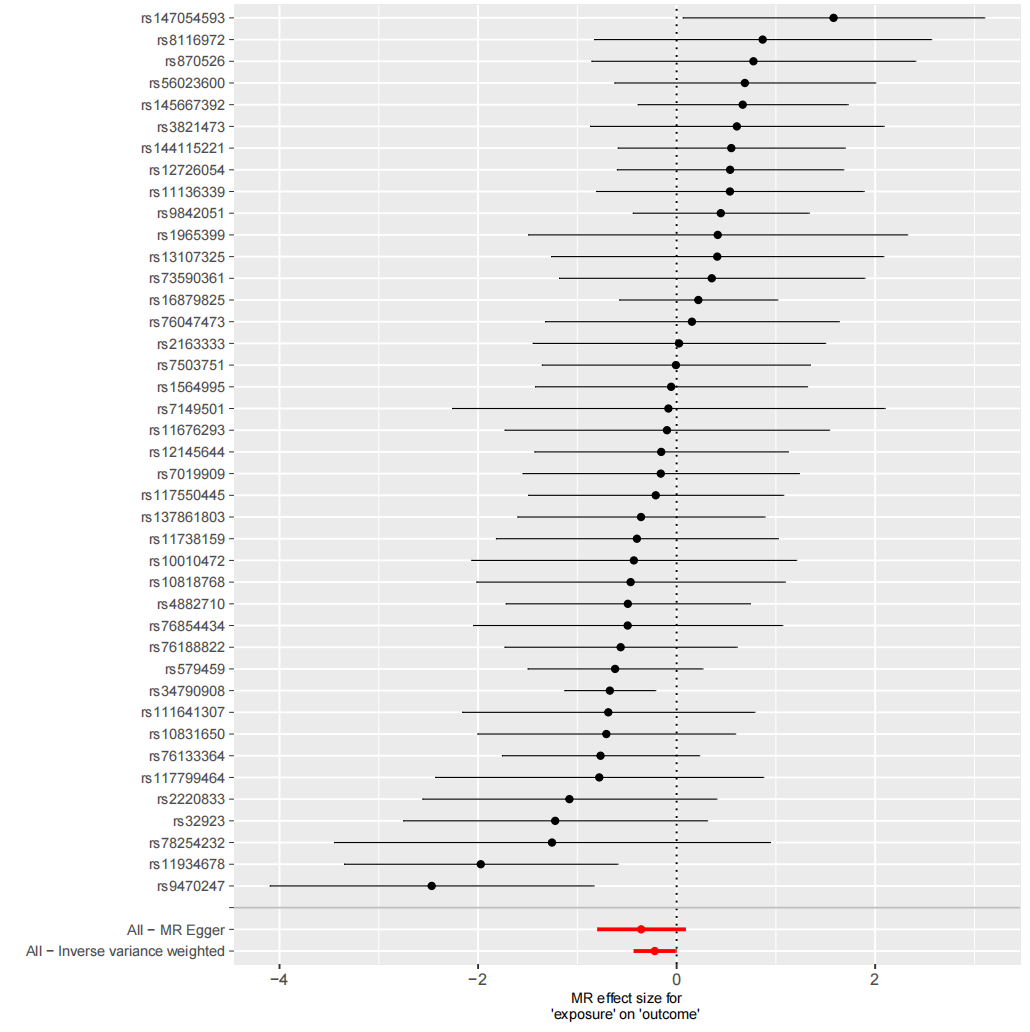

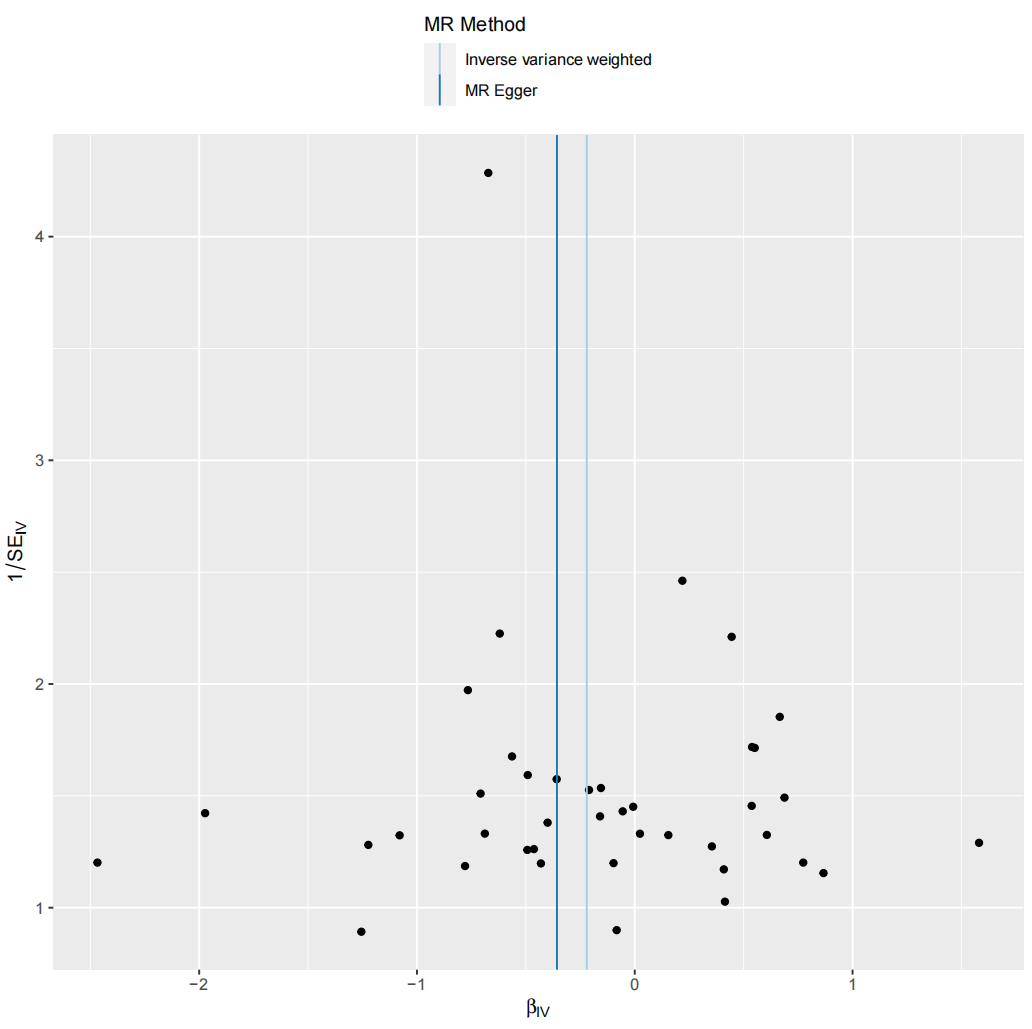


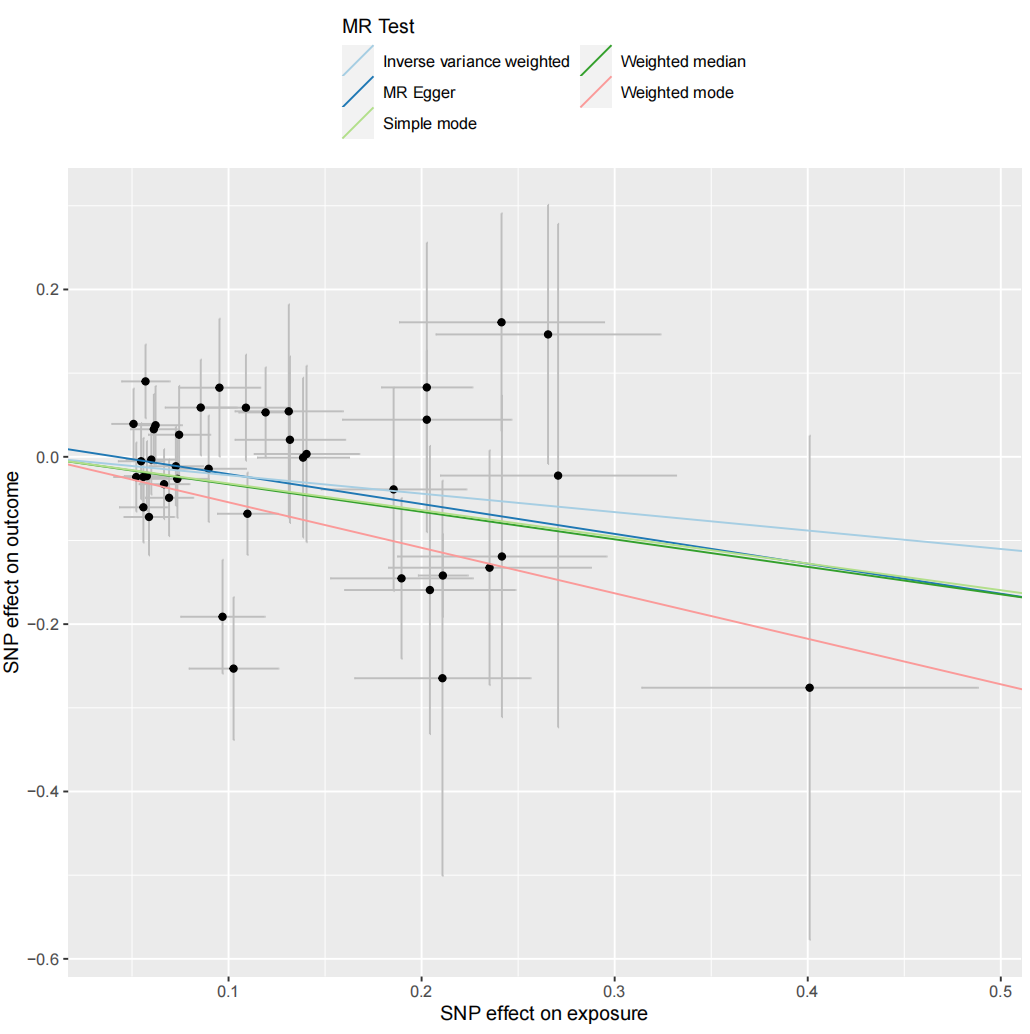

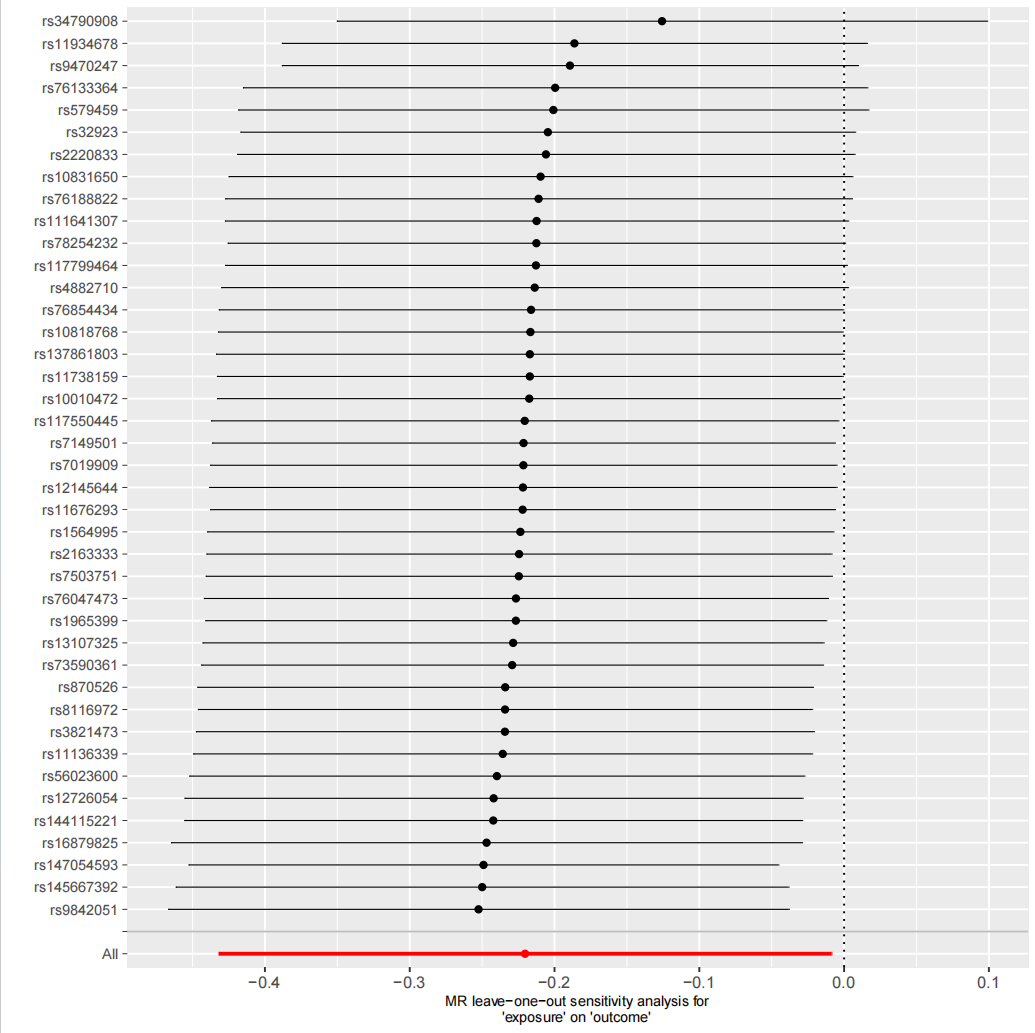


Supplementary Figure S2E Forest plot, funnel plot, scatter plot and sensitivity analysis of SNPs associated with Tumor necrosis factor ligand superfamily member 12 levels on AS.
